# Revising the ECRIN standard requirements for information technology and data management in clinical trials

**DOI:** 10.1186/1745-6215-14-97

**Published:** 2013-04-05

**Authors:** Christian Ohmann, Steve Canham, Catherine Cornu, Jochen Dreß, François Gueyffier, Wolfgang Kuchinke, Enrico B Nicolis, Michael Wittenberg

**Affiliations:** 1 Coordination Centre for Clinical Trials, Heinrich Heine University, Moorenstrasse 5, 40225, Düsseldorf, Germany; 2 Independent consultant, c/o Coordination Centre for Clinical Trials, Heinrich Heine University, Moorenstrasse 5, 40225, Düsseldorf, Germany; 3 INSERM, CIC201; CHU Lyon, Service de Pharmacologie Clinique, Université de Lyon, UMR 5558, 69000, Lyon, France; 4 Centre for Clinical Studies (ZKS), University of Cologne, Gleueler Strasse 269, 50935, Köln, Germany; 5 Service de Pharmacologie Clinique et Essais Thérapeutiques, Hospices Civils de Lyon, Faculté de Médecine Laennec, 7 rue Guillaume Paradin, 69376, Lyon France; 6 Department of Cardiovascular Research, Mario Negri Institute- IRCCS, via la Masa 19, 20156, Milan, Italy; 7 Coordination Centre for Clinical Trials, Philipps University Marburg, Biegenstrasse 10, 35032, Marburg, Germany

**Keywords:** Standards, Information technology, Data management, ECRIN, European, Certification

## Abstract

The pilot phase of the ECRIN (European Clinical Research Infrastructure Network) certification programme for European data centres, in late 2011, led to a substantial revision of the original ECRIN standards, completed by June 2012. The pilot phase, the conclusions drawn from it and the revised set of standards are described. Issues concerning the further development of standards and related material are discussed, as are the methods available to best support that development. A strategy is outlined based on short-lived specific task groups, established as necessary by a steering group drawn from ECRIN-ERIC. A final section discusses possible future developments.

## Update

In 2011, ECRIN (the European Clinical Research Infrastructure Network, funded by the EU's Seventh Framework Programme (FP7)) published a list of standard requirements for data and information technology (IT) management in trials units [1]. Their purpose is two-fold:

• to provide the basis of an ECRIN certification programme, that is, with applicant units audited against the standards to confirm their ability to provide compliant and effective data management services for multinational randomised controlled trials (RCTs), and for ECRIN-supported trials in particular,

• to provide a clear interpretation of regulatory and good practice requirements, in the context of the resources available to non-commercial trials units in Europe, and so act as a general guide to establishing and managing high-quality data management services.

The standards were constructed by ECRIN Preparatory Phase for Infrastructure (PPI) Working Party (WP)10, the ECRIN working group on data centres, based on an initial evaluation of a variety of international, European and national regulations and guidelines relevant to Good Clinical Practice (GCP), data security and IT infrastructures, as well as ECRIN documents produced previously. The group then employed a structured and standardised approach to generate the new standards, with several rounds of dissemination, feedback, face-to-face meetings and telephone conferences used to gradually iterate towards a consensus on data centre requirements. The processes of standard construction are described and discussed in detail in the paper accompanying the list of original requirements [2].

In 2012, after two pilot audits and extensive discussion, the ECRIN standards have been substantially revised. The original set had 230 requirements (146 considered 'minimal' and a further 84 classified as 'best practice') organised in 29 distinct lists. The revised set has now only 139 requirements (all classified as ‘essential’) organised into 21 lists. (Please note 'standard(s)', 'requirement(s)' and 'standard requirement(s)' are equivalent and used interchangeably).

The standards are now also supplemented by ‘Explanation and Elaboration’ (E&E) material (a term borrowed from the CONSORT initiative [3]) to provide clarification and justification, example scenarios, discussion of related practice and examples of the evidence required to demonstrate compliance.

This paper describes and discusses these changes. It first provides a narrative of the pilot phase of the ECRIN certification scheme, and then a description of the revision of the standards and the factors that drove that revision. Additional file 1 illustrates the types of changes made, using three example standards. The current set of ECRIN standards is then briefly summarised. The issues still facing standard development are discussed, as are the methods available to develop, review and disseminate standards and possible alternative approaches for the future. A final section discusses possible future developments. The final standards are included as supplements to this paper: in the first, the standards are represented as a simple list of requirements (Additional file 2); in the second, each standard is presented with its associated explanation and elaboration material (Additional file 3). This dichotomy will allow users to quickly find standards and to find help for reviewing these standards.

### The ECRIN pilot phase

There had always been an intention by ECRIN to pilot the standards after their initial publication. Accordingly, a call was launched on 1 June 2011, with a closing date of 31 August, which invited ‘clinical trials units within the national networks linked to ECRIN to become a pilot centre for certification as an ECRIN data centre, and to assist in evaluating the certification process’ [4].

At the same time, an Independent Certification Board (ICB) was assembled to prioritise applications for audit, oversee the certification process and make the final certification decisions. The ICB was designed to bring together senior staff with experience and expertise in clinical trial quality assurance, and IT and data management systems in particular, from a broad geographical spread. In the end, the six members were drawn from Spain, Italy, France, Germany, Denmark and the UK, the membership being discussed and approved by ECRIN-PPI WP10 members.

A small initial team of auditors was also assembled, with the help of recommendations from ECRIN European Correspondents as well as WP10 members. Again, extensive experience and expertise in clinical trials IT and data management systems were sought, not necessarily at the level of seniority of the ICB members, but, for instance, from those with responsibility for such functions within their own unit. In the end, the initial auditor group consisted of one from France, the UK, Sweden, Ireland and Denmark, and three from Germany. A meeting of six of the auditors was held in September, in Paris, to discuss the standards and ensure that there was a shared understanding of the aims of the standards and audits and the methods to be employed. Because the standards are public, we believe we are not as reliant on the opinions of individual auditors as in some other systems; the key requirement for the preparation of auditors is to ensure they share an accurate interpretation of the standards. One of the purposes of the pilot phase was to test this approach in practice.

Four units applied for certification in the pilot phase and the applications were discussed by the ICB in a series of teleconferences. Two units were selected by the board - the Uppsala Clinical Research Centre and the Coordination Centre for Clinical Trials, Heinrich Heine University, Düsseldorf - and audits arranged for November 2011. In both cases a triad of auditors was selected, with at least one native speaker in the audited centre’s language in each team. Audits were designed to last for three days. Auditors were asked to give their judgement on the compliance of the centre with each standard, providing a brief summary of their reasoning and the evidence available in each case.

Both audits were able to be carried out within the planned three days. In both cases the attention of auditors and unit staff was focused more on the 'minimal' requirements, as the ones critical for certification, rather than those merely labelled 'best practice'. While some of the latter were considered, it was not possible to examine them in any detail.

The audit methodology consisted largely of examining and discussing with staff the written and electronic evidence of compliance - including reading controlled documents (for example Standard Operating Procedures (SOPs)), validation records, data extracts, allocation records, and so on - and, where appropriate, inspecting systems and premises. Opening sessions were performed in each case, to clarify the nature and the logistics of the audit with centre staff, and each audit closed with a feedback session where - on an informal basis - the auditors' findings and recommendation to the ICB were summarised.

Both audits took place primarily in English. Local documents were examined in German in the case of Düsseldorf, but usually translated into English in Uppsala. Both units were fully supportive of the audit process, making staff, documents and systems fully available (without compromising patient confidentiality) and both units appeared to find the audit experience a positive one. In each case auditors signed a confidentiality agreement with the unit.

The recommendations of the auditors to the board were similar in both cases - both units had reached almost all of the minimal standards for certification but there were a few gaps which prevented immediate certification. It was felt in both cases, however, that the outstanding issues could be successfully addressed within four months and the units themselves agreed with this assessment.

The ICB had previously agreed that, in such a situation, units would not have to re-apply from scratch. Instead, a short re-audit after the four-month period, to confirm the necessary changes had been made, would be sufficient to allow certification. The recommendation of the auditors, in both cases accepted by the ICB, was that both units should therefore be re-audited, with the expectation that they would meet the criteria for certification at that point.

The major recommendation, however, was that the standards themselves needed extensive revision, to provide a clearer and more widely understood basis for centre certification, a better basis for individual auditors to interpret the standards, and a better guide to good quality IT and data management in non-commercial trials units.

### The revision of the standards

A large number of comments and suggestions were made as a result of the pilot audits (though some of the issues had also arisen during the auditors' meeting in September). It was felt that:

• Considered as a whole, there were too many standards to be assessed within the three-day limits of an ECRIN audit. In particular, the 'best practice' standards should be dropped as they were not essential to the certification process (and in some cases were felt to be confusing).

• Many of the standards, as originally written, were somewhat ambiguous or open to different interpretations.

• Much more supporting/explanatory material was needed for many of the standards, to clarify their practical meaning. Such material could also be used to discuss the 'best practice' associated with that area of work, rather than having best practice standards.

• In several cases, the standards appeared to be measuring sponsor decisions and activity rather than the quality of the data centre itself.

These issues were discussed during the post pilot phase evaluation meeting (Brussels, December 2011), attended by auditors and members of the ICB as well as members of ECRIN-PPI WP9 and 10, and there was general agreement that the standards needed to be revised to meet these concerns.

### Versions 2.0 and 2.1

Version 2.0 of the standards was generated in December 2011 by the chair of the ICB, to reflect the feelings of the review meeting. The 'best practice' standards were removed and the remaining standards re-organised to 22 distinct lists. Efforts were made to clarify and simplify standard statements. Those standards that had been identified as really assessing sponsors were removed or reworded to better reflect the data centre's contribution. A first draft of supporting 'E&E' material was also produced.

All documents were made available in January to those who had expressed an interest (at the December review meeting) in helping to revise the standards. A series of four teleconferences was also organised to review groups of the standards in a more structured way. The set of standards that emerged from this exercise was labelled as version 2.1.

There had been recognition at the December review meeting of overlap between areas considered by ECRIN WPs 9 and 10, in particular in standards dealing with monitoring and pharmacovigilance. The feeling was that it would be better to work on these areas separately, using input from both groups, and remove them from the current set of standards.

As a result, in version 2.1, the list of standards dealing with pharmacovigilance was removed, leaving 21 distinct lists, and standards dealing with monitoring were restricted to the role of the data centre in supporting such activity.

### Final review and version 2.2

**Figure 1:**
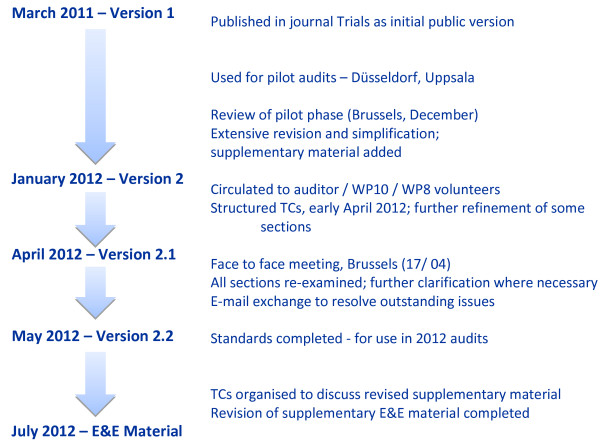
Summary of the review of standards and version evolution.

A final face-to-face meeting took place to complete the review of the standards on 17 April 2012 in Brussels. All standards were considered and several further revisions were agreed. A few standards were the subject of continued email exchanges until the beginning of May when agreement was finally reached. The resulting set of 139 standards, divided into 21 lists, is labelled as version 2.2 and is the current version for 2012 (see Figure 1).

The final stage was to circulate and discuss a revised set of E&E material, and this was carried out amongst a small group in June/July 2012 using teleconferences. Time constraints meant that not all of the support material was discussed in detail, so the intention is to keep this material under continuous review. The standards themselves, however, should only need to be reviewed and revised annually. The final standards are included as supplements to this paper, both as a simple listing (Additional file 2) and as the extended document with E&E material included (Additional file 3).

### Specific vocabulary

It was also necessary to develop a glossary of definitions as part of the review process, to reduce ambiguity in the standards.

For the most part the definitions are of relatively common terms but provide their specific meaning within the context of the ECRIN standards. Examples include 'centre', which was used to indicate the trials units, research centre, data centre (and so on) being audited, while 'site' was used for the clinical setting generating the data.

A few terms were developed specifically for the standards. The most significant of these was 'CDMA', for 'Clinical Data Management Application'. This was used to refer to the individual database or data application set up specifically for a trial, with all the trial particular screens and logic checks, and was developed to clearly differentiate the trial-specific applications from the underlying Clinical Database Management System (CDMS) and the Database Management System (DBMS) used to support it.

### Examples of standard revision

Additional file 1 includes three examples of standards and their supporting material and compares their original and final versions, explaining why the changes were made.

The clarification of the meaning and wording of standards, the use of consistent terminology and the introduction of E&E material to further explain both the criteria and the evidence required to demonstrate compliance should make future audits much easier, though those audits will remain under review. The clarification of the standards and their meaning should also allow trials units to assess themselves against the standards much more easily, and gain a better idea of their ability to gain certification as an ECRIN data centre.

### The current ECRIN standards

**Figure 2:**
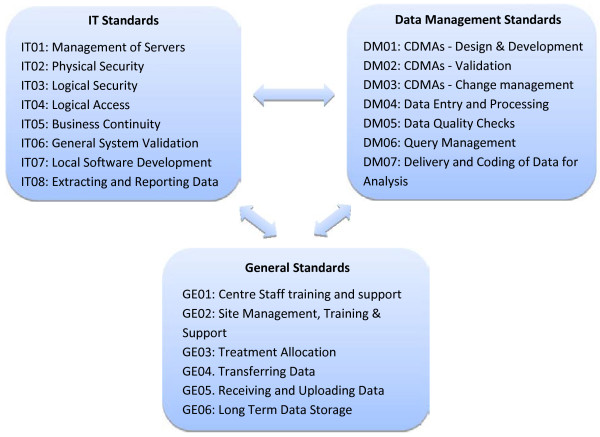
**The revised standard lists** - **version 2.2.**

The 21 lists in version 2.2 are divided into three groups, as shown in Figure 2. Most lists have between five and ten standards. The IT and data management groupings are self-explanatory, while the ‘general’ group comprises a mix of topics that either deal with general centre-wide characteristics like training or span both IT and data management, like treatment allocation.

As the double-headed arrows between the groups indicate, the three groups are not distinct: in reality there is considerable overlap between them.

### Standards by focus

The standards can also be grouped by their 'focus', that is the type of requirement they represent. For instance, the initial standard in many lists contains a requirement for a controlled document (for example a Standard Operating Procedure) dealing with the topic under consideration. In total, there are 21 standards (15%) focused on the centre's Quality Management System (QMS) by explicitly requiring, as part of the standard, controlled documents covering specified topics to be available.

Very many of the other standards, however, also include 'relevant controlled documents' amongst their specified evidence, so a mature QMS is an essential pre-requisite for an applicant unit.

A categorisation of the standards was carried out using the requirement type rather than the specific procedure or functional area responsible for its fulfillment. The categories were defined by the author group.

**Figure 3:**
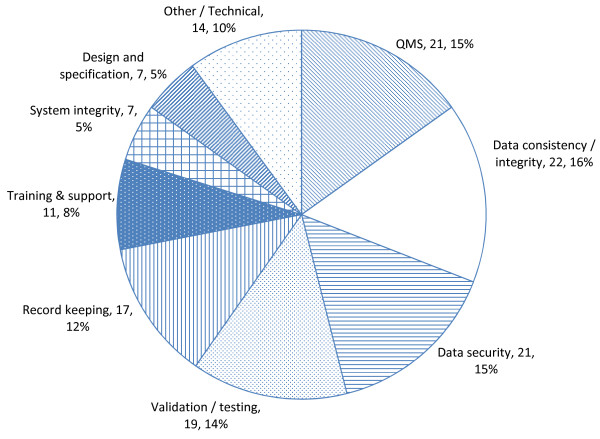
Standards grouped by 'focus'.

As shown in Figure 3, similar proportions of standards are concerned with maintaining data consistency and integrity (16%), data security and access control (15%), and validation and testing (14%). Standards in all of these major categories can be found within each of the IT, DM and GE lists, underlining the fact that the lists overlap considerably in practice.

Seventeen of the standards (12% of the total) are concerned with record keeping of some sort, including retention of data snapshots. Other areas identified were training and support (8%), maintaining system integrity (5%) and assuring appropriate specifications and design (5%). Only 10% of standards do not fall within these named groups.

Another framework within which to analyse the standards is provided by Donabedian's triad of structure, process and outcome measures, as first applied to medical care [5].

If one assumes that the outputs of a data centre are datasets that accurately reflect the study source data then using outcome measures - in a short external audit with no access to the original data - is very difficult, and such measures do not appear in the standards. In fact, 127 (91%) of the 139 standards relate to process. Only 12 can be unambiguously identified as relating to structural components, though it is true that many of the process standards are about how structural elements are specified, configured, validated and used in practice. In other words, the standards assume most of the relevant structural components (for instance, a network, external firewalls, a CDMS, and some form of treatment allocation system) are already present, and largely focus on the procedures and people that the centre puts around these components as indicators of quality.

### Current issues with the standards

Some issues will need to be resolved as the ECRIN standards develop further:

*The tension between standards for certification and guidance*: As outlined in the introduction, the ECRIN standards were originally developed to have a dual role:

• to act as a quality assurance benchmark for certifying units as ECRIN data centres, helping to provide an appropriate infrastructure for ECRIN-supported trials,

• to provide a clear description of good quality IT and data management, especially for smaller and newer units still developing those services, and thereby help to raise the general standards of clinical trial management in Europe.

It was the second role that led to the original inclusion of 'best practice' standards. They have now been removed, though some of their content has been included in the E&E material, and/or the introductory material to each list of standards. Focusing the standards more on the certification process makes that process simpler and the audits easier, but it means there is a risk that one of the two original purposes of the standards is now being sacrificed.

It will therefore be necessary to clarify the purpose of the standards, that is to decide to either focus on the certification function in isolation or reiterate that the standards have their original dual role, and should therefore be clearly linked to developing guidance/discussion materials. The second approach would be more coherent, and offers two advantages:

• using the standards for audit should identify areas where additional guidance would be particularly useful (system validation is an example of a topic where this already seems to be the case),

• developing guidance materials can directly inform the further development of related standards.

There will be a challenge, however, in integrating this activity with other parts of ECRIN (for example the ECRIN Campus initiative, designed to act as an information resource for trials units) as well as keeping material up to date with any changes in regulations.

*Standard scope and* ‘*optional*’ *certification areas*: The current scope of the standards is limited to IT and a relatively narrow interpretation of data management. Activities related to monitoring and site management, as well as pharmacovigilance and managing laboratory and biological samples-related data, are omitted from the current standards (apart from a single standard on supporting SDV (Source Data Verification) by others).

This is partly a function of the historical development of the standards and the focus of the WP10 group within ECRIN-PPI (monitoring and pharmacovigilance were considered by other groups), partly a recognition that these areas are not 'core' to the data management function, and a unit could therefore be certified as an ECRIN data centre without providing these services - the sponsor using other service providers as necessary.

It could be very useful to develop additional standards to cover these 'optional' areas. Those units that wished to provide such services (and advertise the fact to potential sponsors) could then be certified against those standards. That would change the application process slightly, as units would then have to indicate which, if any, of these optional areas they would want to have audited, and the auditing team/timetable might also need to be changed accordingly.

There are a variety of issues that remain to be resolved: initially there is the need to confirm that the suggestion of having this additional characterisation of units would be useful to potential sponsors. If that is the case, then decisions are required about the best way of providing this information, that is, by using standards or some other approach (possibly self-assessment). If standards are used, then decisions will need to be taken about which additional sets of standards should be developed and when.

### Need for steering, development and approval mechanisms

The standards were developed by WP10 of ECRIN-PPI, made up of domain experts from the ECRIN member networks. The problem is that the PPI phase of ECRIN has now finished and WP10, and the other WPs of that phase, no longer exist. The review of the standards was carried out by an ad hoc collection of people drawn from WP10, ECRIN auditors and ICB members, but there is currently no group formally set up to either develop or approve new standards and related material.

The certification program is planned to proceed under the management of ECRIN-European Research Infrastructure Consortium (ERIC), the new legal entity that is funded by health ministry monies from member states. This is in contrast to the funding for the ECRIN-IA (Integrated activity) programme, running from January 2012 to the end of 2015, which is funded from EU FP7 grants. Delays in establishing ECRIN-ERIC have meant not just a gap in the programme itself, but also a lack of clarity about how future developments in standards will be orchestrated and linked to the wider ECRIN agenda.

Setting up mechanisms for directing, developing and approving standards and related material is, therefore, an essential early task for ECRIN-ERIC, and needs to be done before the issues discussed above can be resolved. Part of establishing such mechanisms depend on deciding the best methods to use, as discussed below.

### Methods for developing and reviewing the standards

One of the major problems in developing and discussing the standards was the dependence on a relatively small group of people to provide input. Though many people could have become involved (potentially all the membership of WP10, all of the auditors, all ICB members) in practice, active input was limited to a self-selecting group of about eight, though the standards were read by more.

Though smaller groups are often more efficient, for a pan-European initiative these numbers are not satisfactory. Like any other project, ECRIN certification needs shared ownership and involvement if it is to continue to be supported, and it needs to use as wide a range of expertise and experience as possible to support the future development of standards and supporting material.

Various methodological techniques are sometimes suggested as a means of encouraging participation - the use of Wiki-based websites, collaborative working systems and file sharing, Delphi questionnaire methods and so on. In fact, some of these methods were tried during the standards development process (shared files, circulation of spreadsheets and comment collection) and none of them had any appreciable effect on the pattern of participation.

It would appear that more fundamental reasons were behind the low levels of participation in ECRIN standards development, only one of which might be classed as 'methodological':

• *Workload*: most of the people asked to participate in groups are working at fairly senior level and are very, very busy. Taking part in an occasional discussion is one thing but taking on additional time-consuming work, outside of one's normal job, such as reviewing and suggesting revised versions of standards, is extremely difficult for most of the group members.

• *The size of the task*: there were originally over 200 standards, and even the revised set has 139. The full standards document, with all the E&E material, is about 90 pages long. Expecting input on such a large mass of material - when it is not part of normal paid employment - is unrealistic and the scale of the task may itself have been intimidating, discouraging involvement.

• *The meeting format*: this involved teleconferences in most cases. The experience of teleconferences was that they could be very productive with small groups of active participants and with well-defined tasks, but that with groups larger than five or six, and/or more nebulous discussion, it was very easy for participants to become passive listeners, so that the teleconference tended to become dominated by a small group.

In the future it will be important to reduce these barriers to participation whilst still retaining a reasonably efficient process for developing materials and decision making. Rather than trying to retain a large 'standing group' of experts, that self-selects itself down to a small active core, it is suggested that it would be more productive to distribute future work to distinct, smaller and time-limited task groups.

This will be much easier moving forward because, of course, there is now a full set of standards and associated material to act as the basis of further development. ECRIN WP10, on the other hand, was faced with constructing a system from nothing, and therefore had to consider all aspects of the certification system and standards at the same time.

There are three types of tasks that need to be carried out:

• *An annual review of the standards*: the obvious group to do this is the auditors themselves, together with input from the certification board and ECRIN-ERIC. The auditors have the best direct knowledge of how the standards are interpreted and how auditable they are in practice. They also have the greatest incentive to make the standards clear and workable. The review of existing standards will take into account changes in regulations and emerging evidence relating to best practice.

• *Development of new groups of standards*: if required, for instance for areas like monitoring and pharmacovigilance, as discussed above. This will require domain expertise and the establishment of time-limited task groups, under the co-ordination of ECRIN-ERIC. Most groups should not need to exist for longer than three months.

• *Revision and development of supporting material*: sometimes because of concerns raised by auditors, sometimes as part of the broader ECRIN goal of raising standards in clinical trials units generally, input may be required to develop better and/or wider understanding of certain topics. This could involve not just working on the 'E&E' text, but also developing educational materials, discussion papers, collating and comparing experience, and proposing best practice. Again this will require domain expertise and thus specialist task groups, and may also require liaison with the ECRIN Campus initiative.

As envisaged, a typical task group would be usually asked to arrange face-to-face meetings at the beginning and end of the process, with teleconferences (in time perhaps video conferences) in between. Administrative, secretarial and functional support would need to be supplied from the (ECRIN-ERIC) centre. Collaborative working should, eventually, be supported by the new ECRIN website. Most task groups (apart from the auditor group) should not need to include more than eight members.

To encourage participation, group members should also be given some form of public recognition as 'ECRIN external experts', registration on the ECRIN website and joint authorship of any papers produced by the group.

This is a more flexible and hopefully much more accessible structure than the previous single working group. It will require, however, strong central support and co-ordination. This needs to be provided at two levels:

• *Executive co*-*ordination*: part of whatever executive structures are established in ECRIN-ERIC needs to oversee the certification programme, and part of that will be the continued development of the standards. This is necessary to integrate this activity with the rest of ECRIN and to match it against available resources, for instance in deciding upon the numbers and subjects of task groups. It is also necessary to provide a clear policy context in which further development can take place.

• *Central support*: recording meetings and rewriting documents cannot be done in a reasonable time frame unless central support is provided. A central function has also proven very useful in creating and circulating first drafts of material for discussion, and in creating a variety of record, dissemination and display systems. As central support is also required for the certification/audit process, the same staff should be used for both functions.

Both of these central co-ordinating functions should be established soon as part of ECRIN-ERIC. The executive co-ordination and central supporting function, together, would in effect form an ECRIN-ERIC steering group for both the certification and standard development process.

### The future

The current standards and supporting material provide a solid basis for future ECRIN audits but they also represent a summary of high-quality IT and data management practice in non-commercial clinical trials. As such they have potential value, and deserve wider consideration, beyond the certification and audit programme, and indeed beyond ECRIN. We hope, for instance, that the standards will stimulate debate about trials IT and data management, and its resourcing, amongst funders and senior researchers, as well as providing a general benchmark for planning and developing these services in non-commercial trials units throughout the EU.

The standards should therefore be disseminated more widely - both publicly, for example via the new ECRIN website, and within ECRIN itself, in particular to the European Correspondents, the national representatives of each ECRIN member state. The European Correspondents are important not just because they can help to identify auditors and task group members, but because they can assess whether the standards might be useful within their own countries independent of ECRIN audits. Translation into some other European languages could help with dissemination, if the resources can be found to support this.

For example if major funders or national bodies, especially the regulatory authorities charged with carrying out inspections of clinical trials units, can be persuaded to endorse or even use the standards, they become much more significant as a standard.

That would increase the attractiveness of ECRIN certification, but also means that units may wish to use the standards in a self-assessment exercise, for example to help prepare for inspection, which in turn means that ECRIN should consider developing the necessary materials and proformas to support self-assessment.

There are, of course, other groups involved in formulating standards, at national and international level, for example the Society for Clinical Data Management (SCDM) that publishes the Good Clinical Data Management Practices (GCDMP) guide. As described in the original paper [2], we did consider the SCDM requirements during the original construction of the standards, but were concerned that copyright issues could affect their use within an open public standard. The GCDMP standards also tend to assume a global commercial context, whereas we wanted to focus on the non-commercial European setting. We therefore did not make use of them at that time. Nevertheless, it would be very useful to explore the possibilities of convergence between these and other sets of standards and those published by ECRIN, because the principles underlying different sets of standards are the same, all deriving ultimately from the International Conference on Harmonization's guideline on GCP. We therefore need to consider how best to open and maintain a dialogue with relevant groups, for instance by inviting their input into task groups and by performing comparative reviews.

Both of these activities would help to establish the standards in the longer term. The aim should be to create a 'virtuous circle' whereby the standards become better known and more widely used and discussed, increase in significance, scope and quality, and thus become better known and even more widely used, and so on, and so on.

ECRIN also needs to encourage analysis of the impact of the standards at unit, national and international level, for instance gathering data on the costs of meeting standards as well as the perceived benefits, and assessing any organisational impact, for example on the way IT/data management services are organised within non-commercial trials units. This will be difficult but would provide important information to feed back into the standard development and management process.

## Conclusions

It has been possible to provide a substantial revision of the ECRIN standards following a successful pilot of the original standards and the audit process. The new standards are simpler and clearer, and much better supported by explanatory material, and provide a solid basis for future ECRIN audits, though they will need to be kept under review.

There remain a variety of issues to be resolved, particularly about the scope of the standards (and thus whether additional ones should be developed) and the best way of developing the standards and related material in the future in order to maximise participation from domain experts. Small task groups are seen as an effective way of moving forward, coupled with clear central policies and support.

In the future, as well as re-activating the ECRIN certification programme, it will also be important to disseminate the standards more widely, to obtain useful external input and explore how they can best be integrated into national and other frameworks to produce the general raising of standards and quality in trial IT and data management that is desired.

## Abbreviations

CDMA: Clinical Data Management Application: refers to the specific system established to hold the data for a single trial, plus the trial schedule and the specific data collection instruments, that is the eCRFs, that have been set up for the trial; CDMS: Clinical Data Management System: within centres, the system (or collection of systems) that holds the clinical data gathered during trials. CDMSs are specialist software systems and are often purchased from specialist vendors, but may be built and maintained in house; DBMS: Database Management System; E&E: Explanation and Elaboration; ECRIN: the European Clinical Research Infrastructures Network, an FP7-funded infrastructure supporting multinational clinical research projects in Europe. (See http://www.ecrin.org/); ECRIN-ERIC (European Research Infrastructure Consortium): the ECRIN European Research Infrastructure Consortium, a legal entity whose sustainability is supported by Member States, which will be in charge of the operations, including the support to multinational clinical trials selected by the ECRIN Scientific Board and certification; ECRIN-IA (Integrating Activity): the ECRIN Integrated Activity is the fourth and current step of the ECRIN programme, funded by the FP7 Infrastructures programme, running from January 2012 until the end of 2015; ECRIN-PPI: the ECRIN Preparatory Phase for the Infrastructure (2008 to 2011) was the third step of the ECRIN programme; FP7: the Seventh Framework Programme, the funding vehicle within the EU for all research-related EU initiatives; GCDMP: Good Clinical Data Management Practices; GCP: Good Clinical Practice is an international quality standard that ensures the safety of trial participants and the quality of data received; ICB: the International Certification Board, set up by ECRIN-PPI WP10 in 2011 to oversee the certification process and make the final decisions about certification of individual centres; IT: information technology; QMS: Quality Management System; RCT: randomised controlled trial; SCDM: Society for Clinical Data Management; SOP: Standard Operating Procedure; SDV: Source Data Verification; WP: ECRIN Working Party. Within each phase of ECRIN the tasks are divided between different working parties, established from domain experts throughout the ECRIN member countries.

## Competing interests

The authors declare they have no competing interests.

## Authors’ contributions

SC drafted the manuscript and co-ordinated comments and corrections received from other authors. All other authors provided suggestions and comments, and read and approved the final manuscript.

## Authors’ information

CO, SC and EN are members of the ECRIN Independent Certification Board. SC is the chair of that board and co-ordinated the revision of the standards. JD, MW, FG, CC, and SC are ECRIN auditors. JD, MW and SC participated as auditors during the pilot phase. All authors were involved in the review and revision of the standards.

## Supplementary Material

Additional file 1 Examples of standards and their evolution. Click here for file

Additional file 2 Certification of ECRIN data centres. Listing of standards. Click here for file

Additional file 3 Requirements for certification of ECRIN Data Centres with explanation and elaboration of standards. Click here for file
