# Supplementary material for: Revising the ECRIN standard requirements for information technology and data management in clinical trials
Source: Trials. 2013 Apr 5;14:97. doi: 10.1186/1745-6215-14-97 (PMC3653810; doi:10.1186/1745-6215-14-97)
Supplement: Additional file 1 — Examples of standards and their evolution. [file 1745-6215-14-97-S1.docx]

# Appendix 1: Examples of standards and their evolution

To illustrate the nature of the standards and how they have evolved, and the role of the related Explanation and Elaboration (E&E) material, three examples of standards are discussed below.

- - 1. IT03.02 Commitment to data protection
    2. The centre and its staff can demonstrate compliance with and commitment to all relevant data protection legislation, including the provision of related training programmes.

The original version of this standard was:

- - 1. The unit or its parent organisation can demonstrate compliance with and commitment to local data protection legislation, including relevant policies, training and individuals with designated roles (e.g. 'Data protection officer').

During the review an attempt was made to shorten and clarify the wording, replace the potentially confusing 'local' with 'all relevant' and put some of the original requirements within the E&E material. That material also attempts to clarify the meaning and scope of the standard, particularly the key phrase 'relevant data protection legislation', as well as giving a list of the evidence that might be used by auditors to assess if a centre was compliant.

*IT03.02 E&E material:*

A key component of system security relates to data protection legislation and policies.

Here 'relevant data protection legislation' means that which applies in the countries where trials managed by the centre are carried out, not just the legislation of the centre's own country. For instance German and Danish data protection regulations would be relevant to a French centre if that centre was running a trial with centres in Germany and Denmark.

The expectation is that staff are made aware of their legal and ethical responsibilities under data protection, as part of their initial and continued training (whether carried out by the centre or external agencies). Controlled documents should also be available that demonstrates the centre's commitment to data protection and how they comply with relevant legislation.

One or more members of staff, in the centre or the parent organisation or both, should be identified as a 'data protection officer' and be available to provide both local support and guidance and advice to management, where necessary, on potential problems in complying with data protection legislation.

The evidence required to show that the standard has been met includes:

a) controlled documents that describe how the centre implements data protection policies and the responsibilities of members of staff under those policies;

b) One or more staff identified as having special responsibility for ensuring compliance to data protection legislation

c) records of training concerned with data protection (some level of training will be required for all IT / DM staff);

d) interviews with staff to check understanding of data protection requirements and discuss how the systems work in practice.

N.B. The evidence lists provided for standards are not meant to be exhaustive or definitive – they are guides as to what is likely to be required in many cases, but it is recognised that a centre may have particular evidence available that is more appropriate to their particular situation.

A shorter example is provided by

- - 1. GE02.09 User Support - in English
    2. Help desk / web support can be provided in English as well as the data centre's native language

This standard was originally:

- - 1. Help desk and hot line can deal with the language of the users and provide some sort of help

but it was recognised that this was both very vague and unverifiable, and the standard was changed to something much more specific and testable. The E&E material simply explains the standard:

*GE02.09 E&E material:*

With multinational trials user queries and requests may arrive in a variety of languages. No centre can be expected to support all the potential languages staff might use in a cross European trial, but there is a requirement that they can provide such support in English at least.

Evidence would come from direct observation.

The third example is:

- - 1. DM05.04 Supporting source data verification
    2. The centre has procedures for supporting source data verification, as a minimum providing access to its data for those implementing and conducting the SDV

Originally this read:

- - 1. A risk based source data verification regime is implemented as specified in the protocol, with the emphasis on primary target variables and other essential data. A check of primary endpoints and other essential data is conducted

It was realised, however, that the source data verification regime is something determined by the sponsor and not the data centre, and the original standard was therefore inappropriate. It is true that in many cases the 'data centre' will be a trials unit that is itself, or is acting directly for, the sponsor, but within the certification program the applicant units are considered as 'data centres' providing IT and data management services, and the standards are written to reflect this.

More fundamentally, it was also realised that in some cases the organisation carrying out SDV may not be the same as that carrying out the data management. The standard was therefore changed to its present form, which requires only that the centre can 'support' SDV, by providing access to its data. The E&E material for the standard makes these distinctions clearer:

*DM05.04 E&E material:*

The sponsor will normally determine both the SDV strategy required and decide who will be doing the SDV. Pharma sponsors may, for instance, want to use their own monitors for SDV. Even non-commercial sponsors may wish to use a different trials unit for the monitoring / SDV function than for the data centre function.

What a data centre does need to do is support the work of monitors carrying out SDV, by making the trial data available to them. There should therefore be procedures in place for allowing monitors access to the data so that they can inspect and assess it, and for exporting and presenting data on demand, on a subject by subject basis, to monitors.

It would be good practice, though not currently a formal requirement, to further support SDV with reports detailing query rates and late data (or any other indicators of problems during data entry) on a site by site basis.

The evidence that the standard had been met would be the controlled documents describing the relevant procedures, together with explanations from staff about how they worked in practice.
