# Supplementary material for: Revising the ECRIN standard requirements for information technology and data management in clinical trials
Source: Trials. 2013 Apr 5;14:97. doi: 10.1186/1745-6215-14-97 (PMC3653810; doi:10.1186/1745-6215-14-97)
Supplement: Additional file 2 — Certification of ECRIN data centres. Listing of standards. [file 1745-6215-14-97-S2.pdf]

Certification of ECRIN Data Centres

Listing of Standards

Version 2.2
July 2012

EUROPEAN CLINICAL RESEARCH INFRASTRUCTURES NETWORK AND BIOTHERAPY FACILITIES
FP7 Capacities – Research Infrastructure

Certification of ECRIN Data Centres - Listing of Standards

Contents

Introduction 1
IT01 Management of Servers 2
IT02 Physical Security 2
IT03 Logical Security 3
IT04 Logical Access 3
IT05 Business Continuity 4
IT06 General System Validation 5
IT07 Local Software Development 5
IT08 Extracting and Reporting Data 6
DM01 CDMAs - Design and Development 6
DM02 CDMAs – Validation 7
DM03 CDMAs - Change management 7
DM04 Data Entry and Processing 8
DM05 Data Quality Checks 9
DM06 Query Management 9
DM07 Delivery and Coding of Data for Analysis 10
GE01 Centre Staff training and support 10
GE02 Site Management, Training & Support 11
GE03 Treatment Allocation 12
GE04 Transferring Data 12
GE05 Receiving and Uploading Bulk Data 13
GE06 Long Term Data Storage 14
Glossary 15

Authors / Contributors to the document:

Christian Ohmann¶Ŧ	DE	Coordination Centre for Clinical Trials, Heinrich-Heine-University, Düsseldorf
Steve Canham*¶Ŧ	UK	Independent Consultant specialising in clinical trials systems
Jochen Dreß*Ŧ	DE	Centre for Clinical Studies (ZKS), Köln
Michael Wittenberg*Ŧ	DE	Coordination Centre for Clinical Trials, Philipps-University Marburg, Marburg
Enrico Nicolis¶Ŧ	IT	Mario Negri Institute, Milan
François Gueyffier*Ŧ	FR	Clinical Pharmacology and Clinical Trials Department, University Hospitals, Faculté Laennec, Lyon, France
Catherine Cornu*Ŧ	FR	Centre d'Investigation Clinique, Hôpital Cardiologique Louis Pradel, Lyon
Wolfgang KuchinkeŦ	DE	Coordination Centre for Clinical Trials, Heinrich Heine University, Düsseldorf
Susan Lennon*	EI	Molecular Medicine Ireland / ICRIN, Dublin
Jens Lauritsen¶	DK	Dept. of Biostatistics, Odense University, Odense
Nader Salas*	DK	Copenhagen Trial Unit, Rigshospitalet, University Hospital, Copenhagen,
Christian Ruckes*	DE	Inter-disciplinary Centre for Clinical Trials (IZKS) Mainz
Jose Fernandez Sardinya	ES	Hosptital Clinici Provincial, Barcelona, d'Hebrón, Barcelona
Carlos Lorenzo	ES	Hosptital Clinici Provincial, Barcelona, d'Hebrón, Barcelona
Jadranka Rogan	AU	Cyathus Exquirere PharmaforschungsGmbH, Vienna
Catherine Pham	SE	Uppsala Clinical Research Centre (UCR), Uppsala

¶Certification Board Member
*ECRIN Auditor
Ŧ Member of final review group

Introduction

This document details the systems and functionality that a non-commercial trials unit needs to demonstrate if it is to become certified as an 'ECRIN Data Centre'.

It does so by listing a series of standards - some dealing mainly with IT systems, others focused on data management practices, others concerned with more general topics, but all indicative of safe, effective and compliant data storage and data processing.

The 139 standards are divided into 21 different sections, each dealing with a particular topic.

IMPORTANT

1. Several common terms have specific meanings within these standards. Please refer to the glossary for definitions of the terms used.

2. This document is only a simple listing of the standards and is designed for quick reference. A much more complete explanation and interpretation of the standards, which also includes examples of the evidence required to demonstrate compliance, can be found in the ECRIN document "**Requirements for Certification of ECRIN Data Centres with Explanation and Elaboration of Standards**".

3. Any unit considering applying for ECRIN certification should use the comprehensive '… with Explanation and Elaboration…' document referred to above as the basis for assessing readiness for certification and for preparation for audit.

4. Very many of the standards use the word 'should' , e.g. ' There should be a retirement policy for production servers.... ' In English, 'should' can indicate an imperative, conditional or subjunctive mood, depending on context and tone. To clarify for non-native English speakers, the 'should' in the standards is always **imperative**. It is equivalent to '**must**'.

IT01 Management of Servers

IT01.01 Server specification
The centre can demonstrate that the servers (and related equipment) that they use meet their specifications, as determined by the software and functions being supported.

IT01.02 Server configuration records
Detailed records of server configurations must be available, with logs of subsequent updates

IT01.03 Server support
Hardware support arrangements should be in place to allow equipment to be replaced or repaired in accordance with the centre's own planned times for disaster recovery.

IT01.04 Server retirement
There should be a retirement policy for servers (and related equipment) that takes into account usage, expected hardware lifetimes and support arrangements.

IT01.05 Server maintenance
Necessary patches and updates should be identified and applied in a timely but safe manner to server operating systems, utilities and applications.

IT02 Physical Security

IT02.01 Locked server room
Servers must be housed within a dedicated locked room with unescorted access limited to specific roles, known to and reviewable by the centre.

IT02.02 Secured power supply
The power supply to servers should be secured, e.g. by a UPS unit, to allow an orderly shutdown on power failure

IT02.03 Encryption of non physically secured data
Clinical data relating to individuals should only be stored on protected servers and storage devices. It should not be stored on non secured devices (e.g. on laptops, desktops, USB sticks etc.) unless encrypted

IT02.04 Server failure and response
Failure of any server directly supporting clinical trial activity, within normal local business hours, should result in alerts being sent automatically to relevant personnel

IT02.05 Controlled environment
Servers should be housed in a temperature controlled environment

IT02.06 Hazard control - fire alarms
The server room should be fitted with heat and smoke alarms, monitored 24/7

IT03 Logical Security

IT03.01 Security management system
Regular reviews of security (practices, incident analysis, risk assessment, documentation etc.) should occur across all IT systems relevant to clinical trials activity, followed by any necessary corrective and preventative actions.

IT03.02 Commitment to data protection
The centre and its staff can demonstrate compliance with and commitment to all relevant data protection legislation, including the provision of related training programmes.

IT03.03 External firewalls
External firewalls should be in place and configured to block inappropriate access

IT03.04 Encrypted transmission
Clinical data transmitted over the internet to or from the trials unit should be encrypted

IT03.05 Server administrator roles
Administrative access on servers should be restricted to specified members of IT staff

IT03.06 Administrator access management
Administrator access to servers should be subject to agreed access management policies

IT03.07 Internal firewalls
Inappropriate access to clinical trials data from elsewhere in the organisation should be blocked, e.g. by correctly configured internal firewalls.

IT04 Logical Access

IT04.01 Logical access procedures
Controlled documents covering access control to all systems directly supporting clinical trial activity should be in place

IT04.02 Access control management
Each system requiring access controls should have mechanisms, e.g. using roles, group membership, etc., that can be used to effectively differentiate and manage access

IT04.03 Granularity of access
Access control mechanisms should be granular enough to allow compliance with the data centre's own policies on access control

IT04.04 Network log-in management
Network log-in management should be enforced on all users, usually including regular change and / or complexity rules for the log-in password

IT04.05 Remote access
Remote access should be controlled using the same principles as local access control, and should not normally include access to the host's network (unless the user has a pre-existing identity on that network).

IT04.06 Network lockout
Logins to the network should be locked after a locally determined inactivity period, requiring secured re-activation

IT04.07 Administration of access to clinical data
Access rights to systems storing or processing clinical data should be regularly reviewed, changes to access requested and actioned according to defined procedures, with records kept of all rights, when granted, why and by whom.

IT05 Business Continuity

IT05.01 Business continuity plan
The centre should have or be developing a Business Continuity Plan, covering likely action in the event of a major loss of function (e.g. fire, long term power failure, full server failure, sudden loss of key staff).

IT05.02 Back up policies
Controlled documents detailing backup policy, procedures, restores and testing should be in place

IT05.03 Back up frequency
Backups must be taken using a managed, documented regime that ensures that new or changed data is backed up within 24 hours, and which allows the centre to check that the system is operating properly.

IT05.04 Back up storage
Back up media storage (location, protection, redundancy) should be sufficient to avoid data loss if there is a fire or other disaster

IT05.05 Back up - Environment
Any necessary data management / administration data (access groups, log-ins, scheduled jobs etc.) should be backed up and restorable

IT05.06 Recovery Testing
Testing of full restore or failover procedures, should take place and be documented at a frequency that reflects system and staff changes (for all servers relevant to clinical trial activity)

IT06 General System Validation

IT06.01 **Validation policies**
Controlled documents should be in place covering system validation approaches, responsibilities and processes

IT06.02 **Validation system inventory**
The centre should have system inventory documentation, identifying all IT systems relevant to clinical trials activity, the risks associated with each, and - in summary - the consequent validation strategy for each

IT06.03 **Risk based approach to Validation**
The general approach to validation of any system should be based on analysis of potential risk, and take into account the system's usage, users and origins

T06.04 **Validation Detailed Evidence**
Detailed validation documents should exist for any particular system, detailing the validation carried out, including any test data and protocols, and the results obtained

IT06.05 **Validation Summaries**
A signed and dated summary of the results of each validation should exist

IT06.06 **Change Management Policies**
Controlled documents should be in place defining change management mechanisms and their scope, who should authorise and review requests, and how they should be documented

IT06.07 **Change and risk evaluation**
Change management in relation to systems that support clinical trial activities should include a documented risk evaluation (including a review of the need for revalidation) and a record of the consequent decision and actions.

IT06.08 **Change and evidence of re-validation**
Any re-validation associated with a change, of the entire system or parts of it, should be planned, executed and documented as part of the change management process

IT07 Local Software Development

IT07.01 **Documentation of in-house software**
System documentation should cover system architecture, plus identification of individual modules / classes and their inputs, outputs, and purpose

IT07.02 **In line Commenting**
All code should have sufficient in line documentation to support tracing of program execution

IT07.03 **Software development**
The software development methodologies used can support quality assurance techniques and promote ease of future maintenance

IT08 Extracting and Reporting Data

IT08.01 **Report access control**
Access to reports should be controlled and match the users' requirements as well as the relevant regulations and laws.

IT08.02 **Report validation**
The structure and accuracy of reports should be validated.

IT08.03 **Validating extractions**
Any data extraction process should be validated

IT08.04 **Validating transformations**
Any data transformation process should be validated

DM01 CDMAs - Design and Development

DM01.01 **CDMA development policies**
Controlled documents covering the development of CDMAs and CRFs should be in place

DM01.02 **Cross-disciplinary CDMA development**
CDMA and CRF development is performed by a cross-disciplinary team (e.g. investigator, trial manager, statistician, data manager, programmer)

DM01.03 **Requirement specifications of CRF**
The specification for CRFs is driven by the protocol (e.g. primary safety and efficacy variables)

DM01.04 **Design of CRFs**
CRF development is compliant with procedures described in controlled documents and includes version management

DM01.05 **Functional specifications of CRFs**
CRF design and functional specifications exist identifying each data item on each CRF (including field names, types, units, validation logic, conditional skipping)

DM01.06 **Specification approval**
CRF design and functional specifications are signed off and dated by relevant signatories

DM01.07 **Isolation of development CDMAs**
CDMAs in development should be isolated from CDMAs used productively

DM01.08 Isolation of training eCRFs
Access to the CDMA for training purposes is managed to ensure that is isolated from clinical data

DM01.09 Production of interim CRF
For trials / sites using eCRFs, procedures should be in place to generate accurate iCRFs (interim CRFs) for sites, if and when necessary

DM02 CDMAs – Validation

DM02.01 CDMA validation policies
Controlled documents for CDMA validation are in place

DM02.02 CDMA Specific test plan
A trial-specific test plan and a test documentation set exists for each CDMA.

DM02.03 CDMA testing against functional specifications
Testing with sample data against functional specifications is carried out for each CDMA before deployment to live environment

DM02.04 Assessment of CRFs by users
Users are involved in assessing CRFs for ease of use

DM02.05 CDMA approval
Each CDMA should be formally approved, dated and signed by the relevant signatories, before production use.

DM02.06 Validation detailed findings
All validation results, including any test data and protocols, are retained for each CDMA

DM03 CDMAs - Change management

DM03.01 Change management of CDMA
Controlled documents for CDMA change management are in place

DM03.02 Documenting change requests
Individual requests for change to CDMAs are justified, itemised and documented

DM03.03 Change and risk analysis
A risk analysis is conducted and recorded when considering any change

DM03.04 Testing of CDMA changes
Any change is tested in the development / test environment and the test results are recorded

DM03.05 Communicating changes
Mechanisms are in place to inform relevant staff and users of changes, and provide support and explanatory material as required

DM03.06 Changes and protocol revision
Processes should exist to ensure ongoing consistency between a CDMA and the associated trial protocol

DM04 Data Entry and Processing

DM04.01 Data entry policies
Controlled documents for data entry and corrections are in place

DM04.02 Access control for data entry
Access control is fully implemented; data entry / review is only accessible to authorised personnel and according to need

DM04.03 Restriction of site data access
Site staff only have access to the data of their site(s)

DM04.04 Management of missing CRFs / Data
Mechanisms are in place to identify and report on missing or late pCRF / eCRF and safety data

DM04.05 Tracking of pCRFs
For pCRFs a receipt tracking system is in place.

DM04.06 Patient blinding requirements
Processes exist to allow the blinding of inappropriate patient identifying information submitted to the centre

DM04.07 Simple checks used
Simple checks on single values (e.g. range checks) should be available and used where appropriate

DM04.08 Complex checks used
Complex checks on multiple variables (e.g. for logical consistency across forms) should be available and used where appropriate

DM04.09 Self-evident corrections
Clear guidelines and procedures should exist to identify and carry out self-evident corrections

DM04.10 Audit trail
All transactions in the CDMA (insert, update, delete) must have an audit trail, covering the date and time of the input, the person making the change and the old and new values

DM04.11 Timestamp control
Sites using eRDC should not be able to change the CDMS's time stamp

DM05 Data Quality Checks

DM05.01 Data quality policies
Controlled documents are in place regarding data quality and the checking required to support it

DM05.02 Batch validation checks
Validation checks can be executed via a batch process, to identify missing, illogical and inconsistent data and are used where appropriate

DM05.03 Data review
Validated reports are available in formats to support the manual review of data (e.g. for consistency checking, medical review)

DM05.04 Supporting source data verification
The centre has procedures for supporting source data verification, as a minimum providing access to its data for those implementing and conducting the SDV

DM05.05 Documentation of checks
All data checking exercises should be documented and analysed, and any emerging issues reported to the appropriate person(s) for resolution

DM06 Query Management

DM06.01 Query Policies
Controlled documents are available covering query format, generation, data change and resolution

DM06.02 Query creation - data entry
Queries can be created - automatically and / or manually – based on documented staff roles, procedures and pre-constructed logic checks

DM06.03 Query Creation – Batch Process
Queries can be created in accordance with documented procedures from batch checking of data, as necessary

DM06.04 Tracking of queries
Responses are recorded when returned, identified when outstanding and queries resent if necessary

DM06.05 Actions in response to queries
Query resolution is tracked, and appropriate actions taken and documented.

DM07 Delivery and Coding of Data for Analysis

DM07.01 Policies for database locking
Controlled documents should be in place dealing with taking a snapshot of the trial data, and / or 'locking' and 'unlocking' that data

DM07.02 Data completion
All relevant data (or all except for a pre-defined / pre-agreed fraction) should be received prior to data extraction for analysis

DM07.03 Query resolution completion
All queries (or all except for a pre-defined / pre-agreed fraction) have been resolved prior to data extraction for analysis

DM07.04 Data reconciliation
All external data (e.g. safety database, lab data) has been reconciled prior to data extraction for analysis (or all except for a pre-defined / pre-agreed fraction)

DM07.05 Post lock data amendment
Controlled documents should be in place detailing procedures to be followed if data needs to be altered after the snapshot or DB lock

DM07.06 Read only retention of analysis data
The data provided for analysis is retained within a read only regime, and is available as a reference data set for any future re-analysis or audit

DM07.07 Extracted data validation
The data generated for analysis should be validated against the data in the clinical database, or the extraction process itself is validated

DM07.08 Policies for coding
If data coding is carried out, controlled documents are in place detailing the procedures to be used

DM07.09 Coding training
If data coding is carried out, it is carried out only by personnel trained on the relevant systems with access to authorised trial specific support material

GE01 Centre Staff training and support

GE01.01 Policies for training
Controlled documents are in place describing initial and continuing training requirements, policies and procedures

GE01.02 Documentation of training
Records of initial and continuing education are kept for all IT / DM staff.

GE01.03 Identifying training requirements
A mechanism exists to identify training / development requirements, and document these and subsequent actions.

GE01.04 Problem resolution
Staff know who to go to within the organisation to seek advice and resolution of problems.

GE02 Site Management, Training & Support

GE02.01 Policies for site opening and support
Controlled documents for opening and supporting a site for data collection are in place

GE02.02 User training for data entry
User training with data entry instructions or guidelines, for pCRFs and / or eCRFs, is provided for site staff

GE02.03 Test or production environment
There is a clear and consistent on-screen indication to the user if they are working on a test or training eCRF.

GE02.04 Site access to production system
A site is given access to a production CDMA only once the sponsor, or the sponsor’s representative, has confirmed that all relevant preparation, permissions and agreements have been completed

GE02.05 Individual access to production system
Individuals have access to production data only when they have been trained with the CDMS and the specific CDMA.

GE02.06 Site documentation
Processes exist to update and redistribute site documentation when this is required as part of change management

GE02.07 Responsibility list
Processes exist to assure that up to date information of who can do what at each site, including entering data and / or signing off CRFs, is available to data centre staff

GE02.08 User Support - prompt response
The centre is able to provide Help Desk support and / or web based support (details as agreed with sponsors) to provide a rapid initial response to site requests

GE02.09 User Support - in English
Help desk / web support can be provided in English as well as the data centre's native language

GE03 Treatment Allocation

GE03.01 Procedures for treatment allocation
Controlled documents are in place dealing with the set up and management of treatment allocation

GE03.02 Policies for ensuring blinding
Controlled documents exist covering the preservation of blinding (where used)

GE03.03 Policies for Unblinding
Controlled documents are in place to support rapid and safe unblinding of blinded treatments when required

GE03.04 Algorithms and supporting systems
The underlying algorithms and operations of all systems for allocating subjects to treatments must be clearly documented and validated

GE03.05 Specification documentation
Details of the treatment allocation specification for any specific trial should be documented and recorded

GE03.06 Problem Management in Treatment Allocation
Any problems or errors that arise in the treatment allocation process are logged and the subsequent actions recorded

GE03.07 Treatment Allocation Training
All staff who handle allocation requests are adequately trained for each specific trial randomisation process

GE03.08 Record of Allocation
Records of all allocation material generated and all allocation decisions made must be maintained

GE03.09 Failover to Manual
System(s) must be in place, supported by training, to deal with a loss of IT based treatment allocation (if used)

GE04 Transferring Data

GE04.01 Data Transfer Procedures
Controlled documents dealing with the transfer of data from the data centre should be in place

GE04.02 Encryption of Individual Data
Any file(s) transferred out of the data centre that include data relating to individuals should be encrypted

GE04.03 Purpose Recorded
The purpose of the planned data transfers should be known and documented

GE04.04 Assuring Security
The centre sending the data must have a written agreement / declaration from the recipient that the receiving organisation will maintain appropriate security of data (whilst it remains in their direct care)

GE04.05 Format of Data Transfers
Procedures should be in place for agreeing, specifying and documenting the format of the transferred data

GE04.06 Records of Transfers
Details of any specific data transfer should be logged, and include a summary description of the data, sender, recipient and transfer method, and the date sent

GE04.07 Retention of Copies
Copies of the data sent should be retained within a read only regime and be available as a reference data set for audit / reconstruction purposes.

GE04.08 Retention of post-processed data
If data is processed before being transferred, copies of the data as extracted before post processing should be retained as well as copies of the data actually sent

GE05 Receiving and Uploading Bulk Data

GE05.01 Import Procedures
Controlled documents dealing with receiving and uploading bulk data should be in place

GE05.02 File Retention
The original files received should be retained within a read only regime, and be available as a reference data set for audit / reconstruction purposes.

GE05.03 Retention of post-processed data
If imported data has to be pre-processed before upload to the CDMS, copies of the data actually uploaded should be kept within a read only regime

GE05.04 Logging of receipts and uploads
Each receipt and upload process should be documented and logged

GE05.05 Format of received data
Procedures should be in place for agreeing, specifying and documenting the format of the received data

GE05.06 Requests for direct amendment
Procedures should exist to deal with requests for direct changes of data in the database

GE05.07 Recording direct amendments
Any direct amendments made must be logged and the details noted, including the justification for the change

GE06 Long Term Data Storage

GE06.01 Policies for long term storage

Controlled documents are in place concerning long term storage of both trial documents and electronic data

GE06.02 Access to long term storage

Access to physical and electronic long term storage is controlled and removal or re-activation of any documents or data is recorded

GE06.03 Protection of long term storage

Measures are in place to guarantee secure long term storage (e.g. locked rooms and fire-proof cupboards)

GE06.04 Length and Content of Storage

Procedures should be in place to agree with the sponsor the length and content of long term storage.

GE06.05 Data Destruction

Procedures should be in place to support the final destruction of physical and electronic data, as required by regulations and/or sponsor

Glossary

Centre: is used to refer to the organisation or team seeking certification as an ECRIN data centre, even though it may call itself a trials unit, a research centre, a clinical research department, a trials and statistics co-ordination centre, or any one of the many variations on these titles. If there is a risk of ambiguity the term data centre is used.

Clinical data (or 'individual data', or 'data relating to individuals'): is used to refer to any data that is associated with an *individual* trial subject, whether or not it describes a clinical symptom or situation. In particular it could include demographic, treatment and lab details – anything that is considered as relevant to the study and which is an attribute of a single study subject or their experience.

Clinical Data Management Application (CDMA): refers to the *specific* system established to hold the data for a single trial. As well as the data itself, the CDMA contains the schedule and check logic for that trial, and the specific data collection instruments, i.e. the eCRFs, that have been set up for the trial. A CDMA is therefore a specific application of the underlying CDMS.

Clinical Data Management System (CDMS): Within centres, the system (or collection of systems) that holds the clinical data gathered during trials. CDMSs are specialist software systems and are often purchased from specialist vendors, but may be built and maintained in-house. Examples are Medidata Rave, OpenClinica, InferMed Macro, Omnicomm TrialMaster and SAS PheedIt.

Controlled Documents: is the generic term used for *all* quality management documents that are authorised (i.e. signed off as correct and designated for implementation) by one or more people, and which are version controlled. They include SOPs and work instructions, and most policies. Most organisations keep their controlled documents within electronic filing systems and apply document management to differentiate the various versions. Because different units designate different controlled documents differently within their quality management systems the standards always use the generic 'Controlled Documents' rather than the more specific SOPs, work instructions etc.

CRF: is the generic term used for all types of Case Report Form(pCRF, eCRF, iCRF).

Data relating to individuals: = clinical data, as defined here

Database Management System (DBMS): This refers to the underlying data storage system for a CDMS, often known as the 'back end' database. Almost all CDMSs use a commercial database system for data storage, e.g. Microsoft's SQL Server, Oracle, PostgreSQL, or MySQL. Most use a relational table structure and some variant of SQL (Structured Query Language) to access and edit data and table structures.

eCRF: In the context of eRDC the electronic screen based case report form, used for direct input into the CDMS from the clinical site. eCRFs normally include validation and range checks so that unlikely values can be flagged, and errors corrected, during initial data entry.

eRDC: is the term used here for electronic remote data capture, i.e. data entry direct from sites. In most eRDC systems access for data entry will be via a web browser.

iCRF: (interim CRF) In many cases it is not practical for research staff to access eRDC systems while interviewing patients and / or collating information, and in any case many staff prefer not to do so, feeling it is disruptive to the interview and uncomfortable for the patient. In such cases it is useful to have a paper version of the eCRF, to capture data in a structured and accurate way, rather than simply making notes freehand. This paper CRF, probably printed from the eRDC system and used / retained within the clinical site, i.e. not sent to the trials unit, is here referred to as an interim or iCRF.

individual data: = clinical data, as defined here

IT host organisation: is the organisation responsible for managing a particular component of the centre's IT systems – exactly which component will vary with the context. To keep things simple the body providing the IT component, which might be the centre itself, it's parent organisation or an external host, are all referred to as the IT host organisation.

Parent organisation: is used to refer to that organisation (or organisations) to which the centre belongs – normally a university or a hospital, sometimes both. In some contexts it may mean in practice just that part (e.g. faculty, clinical directorate) which directly contains the centre, in others the whole organisation.

Patient Identifying Data (or PID): any data within clinical data that could potentially be used to identify subjects, either directly or by linkage to other systems. PID obviously includes names and initials, but also hospital system IDs or national health service / insurance IDs, numbers which in conjunction with those systems would identify an individual. Dates of birth can be PID, though normally not in a large data set and without other associated data (e.g. identifying source hospital) when identification would be difficult. *There is no absolute definition of PID* - it depends on the size of the data set and what data is present. Any clinical data can be PID if it is rare, in a small data set, or linked to other information (e.g. geographical location).

pCRF: The traditional paper based case report form, distributed by the trials unit to the sites and then returned completed, usually by post or courier.

Policy: A fairly general statements of the aims of the organisation with regard to a particular aspect of functioning. Policies will usually be distinct documents approved by a senior manager or committee, and may or may not include a broad brush description of how the policy should be carried out. Some policies may only be written down only as minutes of meetings, however, so not all will necessarily be formerly controlled documents. Policies would normally trigger the production of supporting SOPs.

Remote access: as used here, is *not* the same as eRDC. It refers instead to the process whereby collaborators (including other trials units) and centre staff working away from the centre premises gain access to the CDMS using technologies like Citrix, Terminal services or VPN, as well as browser based methods. This may involve data entry, but could also include other functions like entering monitoring results, or even CDMA design. Remote access is therefore a more general term than eRDC, and can include a wider range of access methods and functionality.

Site: is used for the various clinical and other data collection locations that are participating in a trial and that provide the data to the centre.

Systems directly supporting Clinical Trials: This phrase, and minor variations of it, refers to all systems that store or process trial clinical data or analyses, trial administration and financial data, or trial specific documents (e.g. protocols, agreements), i.e. all things that directly support trial activity and that would stop or disturb that activity if they malfunctioned. It *excludes* systems exclusively used for development, testing and training, and systems that only store non trial specific documents and data (e.g. general centre inventories, staff and budgetary information). It *includes*, however, mirrored or back up servers, even if they are normally passive partners, that could be called into immediate action as part of a failover mechanism.
