# Supplementary material for: Revising the ECRIN standard requirements for information technology and data management in clinical trials
Source: Trials. 2013 Apr 5;14:97. doi: 10.1186/1745-6215-14-97 (PMC3653810; doi:10.1186/1745-6215-14-97)
Supplement: Additional file 3 — Requirements for certification of ECRIN Data Centres with explanation and elaboration of standards. [file 1745-6215-14-97-S3.pdf]

Requirements for Certification of ECRIN Data Centres

with
Explanation and Elaboration of Standards

Version 2.2
July 2012

EUROPEAN CLINICAL RESEARCH INFRASTRUCTURES NETWORK AND BIOTHERAPY FACILITIES
FP7 Capacities - Research Infrastructures

Requirements for Certification of ECRIN Data Centres

Contents

1. Introduction 1

2. Notes on the Audit Process 5

3. Key Terms and Abbreviations 7

4. The Standards 11

IT01 Management of Servers 12

IT02 Physical Security 17

IT03 Logical Security 22

IT04 Logical Access 27

IT05 Business Continuity 31

IT06 General System Validation 35

IT07 Local Software Development 42

IT08 Extracting and Reporting Data 44

DM01 CDMAs - Design and Development 48

DM02 CDMAs - Validation 53

DM03 CDMAs - Change management 56

DM04 Data Entry and Processing 59

DM05 Data Quality Checks 64

DM06 Query Management 67

DM07 Delivery and Coding of Data for Analysis 69

GE01 Centre Staff training and support 73

GE02 Site Management, Training & Support 75

GE03 Treatment Allocation 79

GE04 Transferring Data 83

GE05 Receiving and Uploading Bulk Data 86

GE06 Long Term Data Storage 89

Authors / Contributors to the document:

Christian Ohmann¶Ŧ	DE	Coordination Centre for Clinical Trials, Heinrich-Heine-University, Düsseldorf
Steve Canham*¶Ŧ	UK	Independent Consultant specialising in clinical trials systems
Jochen Dreß*Ŧ	DE	Centre for Clinical Studies (ZKS), Köln
Michael Wittenberg*Ŧ	DE	Coordination Centre for Clinical Trials, Philipps-University Marburg, Marburg
Enrico Nicolis¶Ŧ	IT	Mario Negri Institute, Milan
François Gueyffier*Ŧ	FR	Clinical Pharmacology and Clinical Trials Department, University Hospitals, Faculté Laennec, Lyon, France
Catherine Cornu*Ŧ	FR	Centre d'Investigation Clinique, Hôpital Cardiologique Louis Pradel, Lyon
Wolfgang KuchinkeŦ	DE	Coordination Centre for Clinical Trials, Heinrich Heine University, Düsseldorf
Susan Lennon*	EI	Molecular Medicine Ireland / ICRIN, Dublin
Jens Lauritsen¶	DK	Dept. of Biostatistics, Odense University, Odense
Nader Salas*	DK	Copenhagen Trial Unit, Rigshospitalet, University Hospital, Copenhagen,
Christian Ruckes*	DE	Inter-disciplinary Centre for Clinical Trials (IZKS) Mainz
Jose Fernandez Sardinya	ES	Hosptital Clinici Provincial, Barcelona, d'Hebrón, Barcelona
Carlos Lorenzo	ES	Hosptital Clinici Provincial, Barcelona, d'Hebrón, Barcelona
Jadranka Rogan	AU	Cyathus Exquirere PharmaforschungsGmbH, Vienna
Catherine Pham	SE	Uppsala Clinical Research Centre (UCR), Uppsala

¶Certification Board Member
*ECRIN Auditor
Ŧ Member of final review group

1. Introduction and Background

This document describes the systems and functionality that a non-commercial trials unit needs to demonstrate if it is to become certified as an 'ECRIN Data Centre'. It does so by listing a series of standards - some dealing mainly with IT systems, others focused on data management practices, others concerned with more general topics, but all indicative of safe, effective and compliant data storage and data processing.

The 139 standards are divided into 21 different sections, each dealing with a particular topic. Each section is prefaced by a short statement clarifying the scope of the standards within it, or discussing some general issues about those standards.

Each standard is then presented, along with some 'Explanation and Elaboration' material (the term has been borrowed from the Consort initiative: http://www.consort-statement.org/consort-statement/overview0/). This material has been added to clarify what the standard means, for instance by providing examples, and to describe the evidence that would normally be used to show that it had been met.

In a few cases additional material has been added at the end of a section to discuss best practice in that area, over and above the ECRIN requirements.

The ECRIN standards are designed to be used as the basis of an on-site audit by appointed ECRIN auditors, who then report their conclusions to ECRIN's In dependent Certification Board (see section 2 for further information on the audit process). They are also designed to be used by units for self-assessment purposes, and as a general guide to what is considered to be good quality practice in clinical trials IT and data management.

The focus of the standards, the audit and the certification is the *IT and data management activitie*s of a research unit, even though that unit will usually be involved in many other aspects of the research process - writing protocols, gaining approvals, analysing results, publishing papers etc. This is why throughout the document the research unit is referred to as a 'data centre', or more often just the 'centre'. It is the IT and data management services that the unit can provide, for itself, for external sponsors, and potentially for other research units, that are under consideration.

Certification as an ECRIN data centre is not just an indicator of good quality systems. It is the intention of ECRIN to maintain and publicise a central list of data centres and, once sufficient units have been certified, to encourage the sponsors of ECRIN supported trials to use those centres to provide the data management infrastructure for their trials.

Origin of the Standards
The standards are based upon the principles laid out in the International Conference on Harmonisation's guidelines on Good Clinical Practice (ICH GCP). In many cases, however, these guidelines, as applied to IT systems and data management (DM), are rather vague. ECRIN Working Party 10, working from 2008 to 2011, considered the GCP guidelines, along with many other international and national documents and regulations, and used them to derive a set of more detailed IT and DM specific standards for non-commercial trials units that could be applied across Europe.

The rationale for the standards and the way in which they were developed is described in more detail in a paper published in the journal Trials: Ohmann et al., *Standard requirements for GCP-compliant data management in multinational clinical trials*, Trials, 2011; 12:85; available on the web at: http://www.trialsjournal.com/content/12/1/85

Version Development and Review
The initial version of the standards was published as a supplementary paper to the Trials paper above, in March 2011. This was the version used for the initial audits within the data centre certification pilot phase, at Düsseldorf and Uppsala, in November 2011. This version had 230 standards, divided into 146 considered 'minimal' or 'essential' and a further 84 categorised as 'best practice'. The standards were divided into 29 distinct lists, each dealing with a specific topic.

A large number of comments and suggestions were made by the auditors during the pilot phase. In general it was felt that:

a) There were too many standards to be assessed within the three day limits of an ECRIN audit, and that the 'best practice' standards should be dropped as they were not essential to the certification process (and in some cases could be confusing).

b) Many of the standards, as originally written, were unclear or open to different interpretations, or difficult to assess by external auditors in the time available.

c) Much more supporting / explanatory material was needed for many of the standards, to clarify their exact meaning. Such material could also be used to discuss the 'best practice' associated with that area of work, rather than having best practice standards.

d) In several cases the standards were measuring sponsor decisions and activity rather than the quality of the data centre itself.

These issues were discussed during the post pilot phase evaluation meeting (Brussels, December 2011), attended by auditors and members of the Independent Certification Board (ICB) as well as members of ECRIN-PPI working groups 9 and 10, and there was general agreement that the standards needed to be revised to reflect these concerns.

Versions 2.0 and 2.1
Version 2.0 of the standards was generated in December 2011 by the chair of the ICB, reflecting the feelings of the review meeting. The 'best practice' standards were removed and the remaining standards re-organised to 22 distinct lists. Efforts were made to clarify and simplify standard statements. Those standards that had been identified as really assessing sponsors were removed or reworded to better reflect the data centre's contribution. A first draft of supporting 'Explanatory and Elaboration' material was also produced.

All documents were made available in January to those who had expressed an interest (at the December review meeting) in helping to revise the standards. A series of 4 teleconferences were also organised to review groups of the standards in a more structured way. The set of standards that emerged from this exercise was labelled as version 2.1.

There had been recognition at the December review meeting of overlap between areas considered by WPs 9 and 10, in particular in standards dealing with monitoring and pharmacovigilance. The feeling was that it would be better to work on these areas separately, perhaps using input from both groups, and remove them from the current set of standards.

As a result, in version 2.1, the list of standards dealing with pharmacovigilance was removed, leaving 21 distinct lists, and standards dealing with monitoring were restricted to the role of the datacentre in supporting such activity.

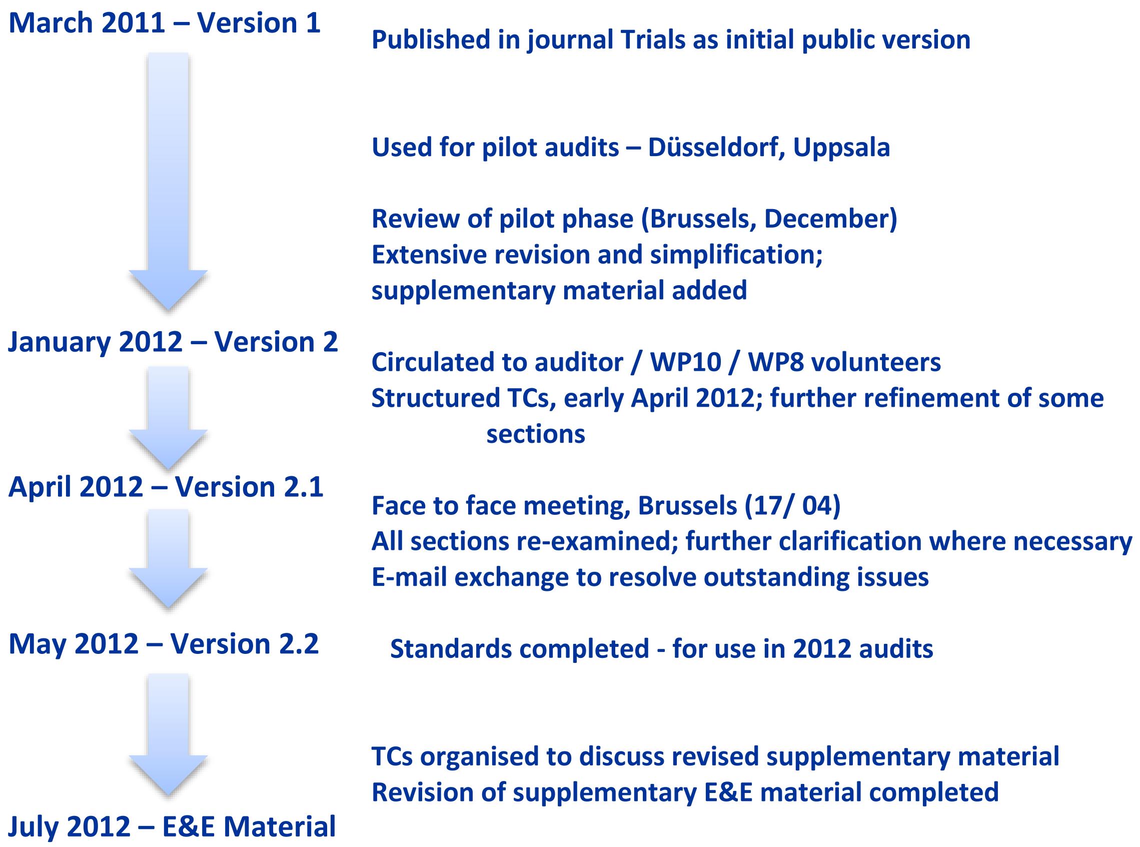

Figure 1: Summary of the Review of Standards and Version Evolution

Final review and version 2.2

A final face to face meeting took place to complete the review of the standards on the 17th April in Brussels. All standards were considered and several further revisions were agreed. A few standards were the subject of continued email exchanges until the beginning of May when agreement was finally reached. The resulting set of 139 standards, divided into 21 lists, are labelled as version 2.2 and is the current version for 2012 (see figure 1).

The final stage was to circulate and discuss a revised set of Explanation and Elaboration material, and this was carried out amongst a small group in June / July 2012 using teleconferences. Time constraints meant that not all of the support material was discussed in detail, so the intention is to keep this material under continuous review. The standards themselves, however, should only need to be reviewed and revised annually.

The Organisation of the Standard Lists

The 21 lists in version 2.2 are divided into three groups, as shown below. Most lists have between 5 and 10 standards. The IT and data management groupings are self-explanatory (a CDMA or Clinical Data Management Application is the individual database or data application set up for a trial, with all the trial specific screens and logic). The 'General' group comprises a mix of topics that span both IT and data management.

IT Standards
IT01: Management of Servers
IT02: Physical Security
IT03: Logical Security
IT04: Logical Access
IT05: Business Continuity
IT06: General System Validation
IT07: Local Software Development
IT08: Extracting and Reporting Data

Data Management Standards
DM01: CDMAs - Design and Development (CDMA = Clinical Data Management Application)
DM02: CDMAs - Validation
DM03: CDMAs - Change management
DM04: Data Entry and Processing
DM05: Data Quality Checks
DM06: Query Management
DM07: Delivery and Coding of Data for Analysis

General Standards
GE01: Centre Staff training and support
GE02: Site Management, Training & Support
GE03: Treatment Allocation
GE04. Transferring Data
GE05. Receiving and Uploading Data
GE06: Long Term Data Storage

Next steps

Certification and the management of the ECRIN standards will fall under the remit of ECRIN-ERIC, so future developments and activity will be dependent on the resources, structures and processes currently being established within that organisation.

Within the next revision it is hoped to extend the scope of the standards by adding sections dealing with more specialist areas of data management. Monitoring and pharmacovigilance are the two areas likely to be considered first, once the relevant groups of experts have been established within ECRIN. Other potential areas include managing biological samples and laboratory derived data.

Developing these additional sections, on top of the core IT and data management standards, will allow units the option to become certified as providers of these additional services (see 'Organisational Choices' in the next section).

2. Notes on the Audit Process

A unit that applies for ECRIN certification (normally as part of an annual call for applications) will have their application reviewed by the ECRIN Independent Certification Board (ICB). If the initial application indicates that the unit can probably meet the ECRIN criteria, an audit will be arranged at a time suitable to the applicant unit.

ECRIN Audits are currently planned to last three days, and normally involve a team of three auditors, with the audit results and auditors' recommendations being passed to the ICB, who make the final decision about the certification of a unit as an ECRIN data centre.

A centre will be awarded certification if the ICB is confident all criteria have been met. If most of the standards have been achieved, and the auditors estimate that the remainder could be met within 4 months, the ICB may allow a provisional certification, to be confirmed by a smaller follow up audit normally within the 4 month period. Otherwise the unit will need to re-apply at a later date for ECRIN data centre status.

The audit itself will normally be conducted in English, but ECRIN will try to ensure that the audit team will include at least one individual who can speak, natively, the language of the data centre, so that all evidence can be inspected. If this is not possible for some reason then the centre may be asked to translate critical documents into English.

ECRIN auditors will be happy to sign confidentiality agreements with audited centres.

Evidence sought by auditors
Many trials units have experienced radical changes in their processes and procedures in recent years, so that data and IT management now may be radically different than it was even 2 or 3 years ago. ECRIN auditors are interested in the arrangements made for *current* and *future* trials, so will focus their inspection on recent activity and trials that have begun recently, usually within the last 12 to 24 months.

Auditors will expect to see a well developed quality management system within any candidate unit, with current SOPs and other controlled documents describing most of the areas covered by the standards. Such documents are not sufficient, however - evidence will also be sought of these controlled documents being implemented in practice, by examining trial specific documentation and specific logs, validation records, agreements, meeting minutes, e-mails, etc., as well as interviewing staff. Direct inspection of the centre's systems, especially the clinical data management system (usually only with dummy or test data) will also be required.

The specific evidence that would be expected for each standard is included in this document as part of the Explanation and Elaboration material. This describes only the most common evidence that auditors would expect to see, however, and in any particular case there may be more appropriate evidence available, more relevant to the particular situation of a specific data centre. The references to expected evidence should therefore only be seen as a guide, not as absolute requirements.

Organisational Responsibilities

In some cases part or all of the functionality covered by a standard may not be the direct responsibility of the trials unit itself, e.g. it may be provided by the parent organisation, or a commercial host, or another collaborating trials unit. Examples are IT services provided by a university or hospital central IT department rather than the trials unit, or a randomisation service provided by an external body.

In such cases the candidate ECRIN Data Centre would be expected to carry out an audit exercise itself on its 'supplier', to ensure that the relevant standards are met by the organisation(s) that provides them. These will often be supplemented by formal written agreements, e.g. in the form of contracts or SLAs, but these on their own are insufficient without evidence they are implemented in practice. Auditors will expect to see the detailed evidence gathered (i.e. by the centre) to support the claims made by third party service providers that they are compliant with the standards.

For standards where this scenario is particularly common this point is re-iterated in the Explanation and Elaboration material.

Organisational Choices

The standards in the current lists focus on core data management functions and the supporting IT infrastructure. There are a few standards (e.g. those in IT07 on local software development) which might not apply to some units, but the expectation is that the great majority will be relevant to the data centre functionality of most trials units, even though the details may vary considerably - for instance in the types of treatment allocation that the centre is able to provide.

There are several areas of related activity, for instance monitoring, pharmacovigilance, sample tracking and handling translational data, and managing disease specific registries, that some centres may carry out while others do not. It would be very useful for sponsors and others to know which centres could provide such services to a high standard. It is therefore the intention to

a) Try and develop, over time, sets of standards to cover those areas, and

b) Allow centres the *option* to be audited against these standards as well, so that they can publicise this additional functionality as being ECRIN certified, as well as the core data management services.

It is hoped to develop standards dealing with monitoring and pharmacovigilance in the next version of the standards. As part of the ECRIN application process, candidate centres will then be asked to indicate which of these 'non-core' services it can provide for multi-national trials, and which of these it wants audited / certified against the ECRIN standards.

Note that once certified a centre will be expected to enter into a formal agreement with ECRIN-ERIC to allow its details, and particularly the range of services it can provide, to be listed as part of its profile within the ECRIN system.

3. Key Terms and Abbreviations

This section provides explanations of some of the terms and abbreviations used within the standards and supporting material.

Many of these terms are relatively common but because of that are often ambiguous. A more precise definition is therefore provided, at least for their usage in this context. Other terms have been developed to describe particular ideas or entities and are therefore explained here.

Being clear about these terms is necessary for a full understanding of the standards. The definitions have therefore been included here rather than in a separate appendix or glossary, and *should be read before considering the standards*.

Terms referring to Organisations

Centre: is used to refer to the organisation or team seeking certification as an ECRIN data centre, even though it may call itself a trials unit, a research centre, a clinical research department, a trials and statistics co-ordination centre, or any one of the many variations on these titles. If there is a risk of ambiguity the term **data centre** is used.

Parent organisation: is used to refer to that organisation (or organisations) to which the centre belongs - normally a university or a hospital, sometimes both. In some contexts it may mean in practice just that part (e.g. faculty, clinical directorate) which directly contains the centre, in others the whole organisation.

IT host organisation: is the organisation responsible for managing a particular component of the centre's IT systems - exactly which component will vary with the context. To keep things simple the body providing the IT component, which might be the centre itself, it's parent organisation or an external host, are all referred to as the IT host organisation.

Site: is used for the various clinical and other data collection locations that are participating in a trial and that provide the data to the centre. (These are also commonly called centres of course, but an attempt has been made to clearly distinguish them by consistently referring to them as sites).

Terms referring to Systems

Clinical Data Management System (CDMS): Within centres, the system (or collection of systems) that holds the clinical data gathered during trials. CDMSs are specialist software systems and are often purchased from specialist vendors, but may be built and maintained in-house. Examples are Medidata Rave, OpenClinica, InferMed Macro, Omnicomm TrialMaster and SAS PheedIt.

Clinical Data Management Application (CDMA): refers to the *specific* system established to hold the data for a single trial. As well as the data itself, the CDMA contains the schedule and check logic for that trial, and the specific data collection instruments, i.e. the eCRFs, that have been set up for the trial. A CDMA is therefore a specific application of the underlying CDMS.

Database Management System (DBMS): This refers to the underlying data storage system for a CDMS, often known as the 'back end' database. Almost all CDMSs use a commercial database system for data storage, e.g. Microsoft's SQL Server, Oracle, PostgreSQL, or MySQL. Most use a relational table structure and some variant of SQL (Structured Query Language) to access and edit data and table structures.

The relationship between the three types of systems is illustrated by Figure 2 below:

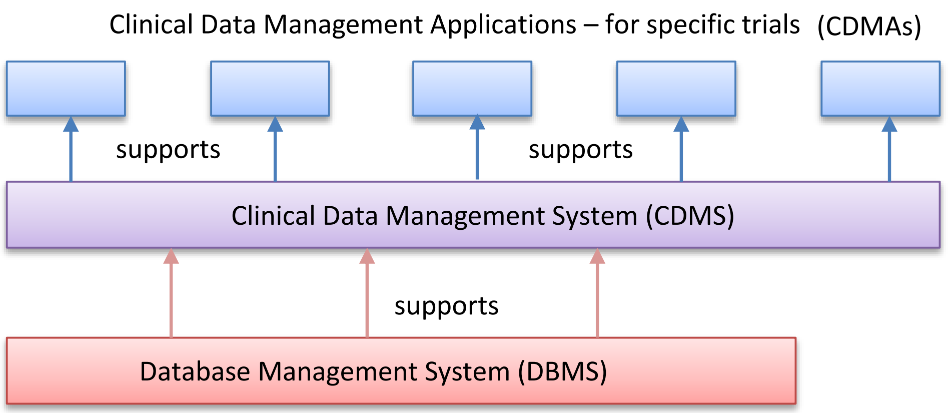

Figure 2: Relationship between CDMSs, CDMAs and DBMSs

'Systems directly supporting Clinical Trials': This phrase, and minor variations of it, refers to all systems that store or process trial clinical data or analyses, trial administration and financial data, or trial specific documents (e.g. protocols, agreements), i.e. all things that directly support trial activity and that would stop or disturb that activity if they malfunctioned.

It *excludes* systems exclusively used for development, testing and training, and systems that only store non trial specific documents and data (e.g. general centre inventories, staff and budgetary information). It *includes*, however, mirrored or back up servers, even if they are normally passive partners, that could be called into immediate action as part of a failover mechanism.

Terms referring to Documents

Controlled Documents: is the generic term used for *all* quality management documents that are authorised (i.e. signed off as correct and designated for implementation) by one or more people, and which are version controlled. They include SOPs and work instructions, and most policies, at least as those are defined below. Most organisations keep their controlled documents within electronic filing systems and apply document management to differentiate the various versions.

Because different units designate different controlled documents differently within their quality management systems the standards always use the generic 'Controlled Documents' rather than the more specific SOPs, work instructions etc.

Policies: are seen as fairly general statements of the aims of the organisation with regard to particular aspects of functioning. They will usually be distinct documents approved by a senior manager or committee, and may or may not include a broad brush description of

how the policy should be carried out. Some policies may only be written down only as minutes of meetings, however, so not all will necessarily be formerly controlled documents. Policies would normally trigger the production of supporting SOPs.

SOPs: can overlap with policies in scope, but must always be controlled documents, with version control and relevant authorisations, application/review dates etc. They provide a more detailed and considered description of procedures to be followed, describing and assigning responsibilities for the tasks and subtasks, and identifying the ordering, inputs and outputs of the processes involved. An SOP should be specific enough to be auditable and provide the necessary guidance to staff.

Work Instructions (WIs): also known as Procedures or Guidance Notes, are the detailed procedural documents (or web pages) that describe how to actually carry out tasks. These documents should also be controlled (i.e. there should be a clearly defined current version) but may not require the full review / authorisation procedure of an SOP. For instance an IT work instruction may be better revised and distributed by the IT manager, in conjunction with his or her team, rather than the full quality management team.

Terms referring to Data

Clinical data, individual data, and 'data relating to individuals': are all used to refer to any data that is associated with an *individual* trial subject, whether or not it describes a clinical symptom or situation. In particular it could include demographic, treatment and lab details - anything that is considered as relevant to the study and which is an attribute of a single study subject or their experience.

Aggregated data: data only about groups of such subjects, as provided in statistical summaries and the research papers derived from the study.

Patient Identifying Data (or PID): any data within clinical data that could potentially be used to identify subjects, either directly or by linkage to other systems. PID obviously includes names and initials, but also hospital system IDs or national health service / insurance IDs, numbers which in conjunction with those systems would identify an individual. Dates of birth can be PID, though normally not in a large data set and without other associated data (e.g. identifying source hospital) when identification would be difficult. *There is no absolute definition of PID* - it depends on the size of the data set and what data is present. Any clinical data can be PID if it is rare, in a small data set, or linked to other information (e.g. geographical location).

Pseudo-anonymised data is data from which the *obvious* PID has been removed, but which contains a unique identifier for each individual subject. That identifier not only groups and labels the data for a single subject, it can also be used as a key to link the data back to the subject's identifying data, if and when necessary. The identifying data must be stored separately (and normally more securely) from the pseudo-anonymised data.

Anonymised data is clinical data from which the obvious PID has been removed, and while it often contains a unique identifier for each subject, that identifier *cannot* be linked to any patient identifying data. Anonymising data is a one way process - once done the data cannot normally be linked back to individuals.

Terms referring to Data Capture
Both forms of data capture, using paper or electronic forms, are recognised as valid and - unless one or other method is explicitly mentioned - all the standards can be taken to apply to both types of data collection. Where the distinction is made, however, the following terms are used:

eRDC: is the term used here for electronic remote data capture, i.e. data entry direct from sites. In most eRDC systems access for data entry will be via a web browser.

Remote access: as used here, is *not* the same as eRDC. It refers instead to the process whereby collaborators (including other trials units) and centre staff working away from the centre premises gain access to the CDMS using technologies like Citrix, Terminal services or VPN, as well as browser based methods. This may involve data entry, but could also include other functions like entering monitoring results, or even CDMA design. Remote access is therefore a more general term than eRDC, and can include a wider range of access methods and functionality.

CRF: is the generic term used for all types of Case Report Form. Three types are recognised:

pCRF: The traditional paper based case report form, distributed by the trials unit to the sites and then returned completed, usually by post or courier.

eCRF: In the context of eRDC the electronic screen based case report form, used for direct input into the CDMS from the clinical site. eCRFs normally include validation and range checks so that unlikely values can be flagged, and errors corrected, during initial data entry.

iCRF: (interim CRF) In many cases it is not practical for research staff to access eRDC systems while interviewing patients and / or collating information, and in any case many staff prefer not to do so, feeling it is disruptive to the interview and uncomfortable for the patient.

In such cases it is useful to have a paper version of the eCRF, to capture data in a structured and accurate way, rather than simply making notes freehand. This paper CRF, probably printed from the eRDC system and used / retained within the clinical site, i.e. not sent to the trials unit, is here referred to as an interim or iCRF.

A note on 'should'
Very many of the standards use the word 'should' , e.g. ' There should be a retirement policy for production servers.... ' Confusingly, in English, 'should' can be used to indicate an imperative, conditional or subjunctive mood, depending on context and tone.

To clarify for non-native English speakers, the 'should' in the standards is always **imperative**. It is equivalent to '**must**'. The only difference is that it sounds (to many English speakers anyway) slightly more polite, especially when an imperative is repeated many times. That does not alter the fact that centres must comply with any 'should' statement if they are to meet the standard that contains it.

4. The Standards

The following pages list the 139 ECRIN standards in their 21 sections. In each case the standard code and title is given in blue, the standard statement in bold black text, and the explanation and elaboration material, and notes on expected evidence, are provided below, as in the following example.

IT01.02 Server configuration records

Detailed records of server configurations must be available, with logs of subsequent updates

The current configuration (operating system version and settings, applications, users, utilities etc.) of each server directly supporting clinical trials activity should be stored. This allows a machine to be….

…

…

The evidence required to show this standard has been met would normally be:

a) Up to date configuration records and patch logs for the servers concerned;

b) Controlled documents detailing how server configuration information is maintained and by whom.

Two important reminders:

1. The use of several common terms have specific defined meanings within these standards and the support material. Section 3 on Key Terms and Abbreviations should therefore be read before examining the standards.

2. The 'evidence required' cited is what would probably be used in any particular case. There may be more appropriate and / or additional evidence available, more relevant to the particular situation of a specific data centre. References to expected evidence should therefore only be seen as guides, not as absolute requirements.

IT01 Management of Servers

The standards in this section are concerned with the servers and related hardware (e.g. network storage) that support the core IT functionality of the data centre. They cover the specification, management and support of that hardware through its life cycle.

Servers and associated equipment may be managed directly by the data centre, or by the host organisation, or by an external hosting facility, or by some mixture of these. Whatever the detailed management arrangements, it will be the responsibility of the data centre to have all the relevant evidence available during an audit. The centre may therefore need to gather material from its service providers beforehand and / or arrange that staff and facilities from those service providers are available during an audit.

Contractual and Service Level Agreements (SLAs) between the centre and service suppliers may form part of the evidence for these standards, but the centre should be able to show that such agreements are actually being met - i.e. there is an expectation that a centre will monitor and document the performance of its service providers.

Note that smaller items such as desktop PCs, laptops, and printers are seen as more straightforward to obtain and configure and are *outside* the scope of this section.

IT01.01 Server specification

The centre can demonstrate that the servers (and related equipment) that they use meet their specifications, as determined by the software and functions being supported.

However servers and related equipment are supplied (i.e. through the traditional procurement of physical servers, or by purchasing virtual servers and related services from the local host organisation or from a remote hosting facility) the functionality and configuration required needs to be specified beforehand.

Specifications may include operating system requirements (e.g. specific versions to support particular products), or hardware specifications (e.g. particular amounts of memory, storage, connectivity etc.), or functionality requirements (e.g. needs to support a number of databases of a certain size with acceptable performance). In other words the specifications should match the centre's needs and those of the software it intends to run.

The data centre should demonstrate that it has the expertise to identify requirements and specify servers and related equipment accordingly, and has been able to obtain the functionality it specified (with, if necessary, additional purchases or processes to bridge any initial gaps).

The most important evidence that this standard is met will come from

a) Controlled documents that provide an overview of the process;

b) the detailed records, including e-mails, of previous and ongoing server acquisitions and / or service purchases , as well as supporting interviews with relevant staff.

IT01.02 Server configuration records

Detailed records of server configurations must be available, with logs of subsequent updates

The current configuration (operating system version and settings, applications, users, utilities etc.) of each server directly supporting clinical trials activity should be stored. This allows a machine to be accurately rebuilt to the same state if necessary, and also permits further work on a server to be carried out safely, based on full knowledge of the machine's existing state.

In some centres server monitoring systems allow configuration information to be updated automatically. In others 'snapshots' of server configurations are taken only at critical time points, e.g. at initial build and before and after major changes. In some cases the 'snapshot' may be a full image of the server, i.e. with all systems and data.

Using periodic snapshots is acceptable as long as there are accurate records of any updates and patches that are applied between those snapshots. All updates should therefore be logged and, along with the configuration snapshot information, the log should always be available (the update log that Windows automatically maintains on a server is not sufficient, because the times that a server becomes inaccessible is exactly when the details are most likely to be needed).

The evidence required to show this standard has been met would normally be:

a) Up to date configuration records and patch logs for the servers concerned;

b) Controlled documents detailing how server configuration information is maintained and by whom.

As mentioned in the introduction to this section these may not be the direct responsibility of the data centre, but the centre will be expected to ensure that such evidence is available during audit.

IT01.03 Server support

Hardware support arrangements should be in place to allow equipment to be replaced or repaired in accordance with the centre's own planned times for disaster recovery.

Centres or their host IT organisation should have a maintenance agreement in place, usually with the original hardware suppliers, to allow for the prompt repair or replacement of critical equipment like servers.

The details of the arrangement(s), and the response times, may vary with the type of hardware provision (e.g. leased versus purchased, virtual versus physical servers) and the type of functionality being supported (e.g. an on-line randomisation service versus a paper based trial, development systems versus production).

The key requirements, that constitute evidence that the standard has been reached, are:

a) controlled documents that include the planned times for disaster recovery for the systems supporting clinical trial activity.

b) documents and / or commercial agreements that detail how repairs and replacements should be managed so that the response times will be achieved (for instance including reference to the agencies used and their contact details).

These functions may not be the direct responsibility of the data centre, but the centre will be expected to ensure that the relevant evidence is available during audit.

IT01.04 Server retirement

There should be a retirement policy for servers (and related equipment) that takes into account usage, expected hardware lifetimes and support arrangements.

Support arrangements for servers and related equipment (e.g. SANs) are usually only available for a finite time, reflecting the increasing risk of failure as a system ages - even though individual machines may continue to operate without problems well beyond that point.

Older machines are also more likely to suffer from a reduction in performance when the load upon them increases, because of more users or more demanding software, and may therefore need replacement. A managed replacement programme to keep machines properly supported and fit for purpose should be in place.

'Fit for purpose' is an important factor - a server may be too slow in a production environment with a few hundred users, but perfectly adequate in a test environment with just one or two. It is therefore not uncommon for a machine to

be 'retired' from production use but still continue in use for many years as a test or training machine.

Leasing and hosting arrangements often have such lifecycle management processes built in, and virtual environments often make the details of the replacement process transparent to the data centre, being managed instead by the host IT organisation. Whoever is responsible for actually carrying out the process, however, the centre should have a policy and / or agreement that ensures the machines it is using are subject to appropriate life cycle management, with retirement from critical functions as necessary.

The evidence that this is the case includes:

a) Controlled documents that outline the server retirement policy and how it operates in practice;

b) Details of how the servers and related equipment currently used by the centre adhere to that policy.

This evidence may not be held in the data centre itself but the centre will be expected to ensure that it is available during audit.

IT01.05 Server maintenance

Necessary patches and updates should be identified and applied in a timely but safe manner to server operating systems, utilities and applications.

This standard requires that there is active management of server patching and upgrades, i.e. a set of procedures that determine how this is done, when, and by whom. Though there can be a risk in *not* applying patches, because they often close security loopholes, there is also an inherent risk in adding a patch or update to a functioning system. Patch management should include safeguards to try and minimise these risks.

In the standard 'utilities' mean things like programs to support anti-malware systems, remote access and backups, whilst 'applications' include (but are certainly not limited to) databases and clinical data management systems. The standard effectively applies to *all* software installed on servers directly supporting clinical trial activities.

Patch testing for operating systems and common applications may be carried out by specialist commercial patch testing services. Using such a service reduces risk but does not eliminate it, so patch management should still include defensive mechanisms (e.g. taking data backups and configuration snapshots) so that the patch can be rolled back and the system restored quickly to its former state if necessary. Patches to less common programs will often need additional management, e.g. application to a test server and evaluation by staff before application to a production server.

The management and timing of patches should involve data centre staff even when the servers are managed by the parent or a hosting organisation, partly to help warn users of any interruptions to services and minimise disruption, partly because only data centre staff are likely to have the expertise to test specialist systems after patches have been applied.

Evidence that the standard was being met would include:

a) controlled documents, detailing how patches / updates should be applied as safely as possible and who is responsible for doing what;

b) specific patch / upgrade records that demonstrate that the patches identified as required have been applied;

c) discussion with the relevant staff about how the system works in practice.

IT02 Physical Security

The standards in this section deal with the physical security of a data centre's systems and data, including not just protection from intrusion and theft but also from environmental threats such as fire, and system threats such as power loss.

IT02.01 Locked server room

Servers must be housed within a dedicated locked room with unescorted access limited to specific roles, known to and reviewable by the centre.

Servers must be located in a locked room, or rooms, specifically allocated for that purpose.

The standard states that the centre, even if it does not manage the server room(s) directly, should still know who is able to have unescorted access to the rooms. Knowing the individual's names may not be very meaningful, and they may change in any case, so knowing which *roles* (or in some cases which teams) have access is more important, as well as the numbers involved for each.

Non IT staff that might have unescorted access include maintenance, cleaning and security staff. The centre should know who, in terms of job title, level of experience and seniority, has access to the server rooms and what the relevant procedures would be (possible reasons for access, logging of individual visits etc.).

The centre should also be able to review the actual patterns of access - who, why and when - to see what the granted access rights mean in practice.

If the centre manages its own server rooms maintaining compliance with the standard and reviewing access will be straightforward. If this is not the case the centre should still be able to review both the list of those with potential access and the actual access, for instance every twelve months. It should be able to make any concerns known to the IT host organisation, seeking a revision of the list if necessary.

Evidence that the standard is being met includes:

a) physical inspection of the server rooms (or at least their associated security mechanisms);

b) controlled documents describing access policies;

c) inspection of the current list of those with access, together with any access logs.

If servers are hosted in a remote facility than the IT host organisation's literature, accreditation, and relevant agreements may need to be utilised rather than direct inspection of facilities and access logs. The centre would be expected to have this evidence available during an audit.

IT02.02 Secured power supply

The power supply to servers should be secured, e.g. by a UPS unit, to allow an orderly shutdown on power failure

Servers and related equipment need to be protected from loss of power, at least to the extent that they can be shut down in an orderly fashion.

Evidence that the standard is met can be obtained from:

a) inspecting the UPS, or perhaps its specification;

b) from examining records of successful testing of the UPS;

c) from records of and discussion about incidents when the UPS became necessary.

If servers are hosted in a remote facility than the IT host organisation's literature, accreditation, and relevant agreements may need to be utilised rather than direct inspection of the facilities and test records. The centre would be expected to have this evidence available during an audit.

IT02.03 Encryption of non physically secured data

Clinical data relating to individuals should only be stored on protected servers and storage devices. It should not be stored on non secured devices (e.g. on laptops, desktops, USB sticks etc.) unless encrypted

This standard says that *any* non aggregated data, i.e. that relates to individual trial subjects, must not be stored on non secured devices unless encrypted. This includes demographic, treatment and lab details as well as data relating to clinical signs and symptoms - anything that is an attribute of a single study subject or their experience.

Secured devices are servers and network storage devices that are physically secured by being in locked rooms, and logically secured by being within the centre's (or its IT host organisation's) firewall. *Non secured devices* include desktop PCs and laptops as well as USB sticks and CDs / DVDs, that are not encrypted. (Desktop PCs can easily be stolen, and frequently are).

No distinction is made between data that contains obvious patient identifying data (PID) and data which does not. This is because PID is hard to define and the distinction is not absolute. Obvious patient identifying data, like name, initials, and health system number stand at one end of a continuum. At the other extreme is anonymised data without any such items, or links to data that might contain them, and without localising data (either in space, such as hospital name, or in time, such as date of birth).

Some individual clinical data without obvious PID is so detailed, however, and / or so rare, that - especially with some localising data included as well - it *can* become potentially identifying. Such data stands somewhere between obvious PID and

anonymised data. To keep things simple and safe therefore, the standard requires *all* data relating to individuals to be encrypted unless it is stored on a secure device.

The level of encryption required should match, as a minimum, the recommendations of the relevant national research or health organisation (128 bit AES in many instances, 256 bit in others). Many centres now routinely provide automatic 'whole-drive' encryption for laptops and USB sticks, which reduces the potential impact of the standard as well as being seen as best practice.

Evidence for the standard being met can come from:

a) the controlled documents describing the policy;

b) direct examination of laptops and desktops;

c) interviews with staff, e.g. to check their understanding of the relevant controlled documents.

IT02.04 Server failure and response

Failure of any server directly supporting clinical trial activity, within normal local business hours, should result in alerts being sent automatically to relevant personnel

If a server does experience some sort of failure it is important that staff are aware of this straightaway, at least during normal local business hours.

Note that this standard covers all servers 'directly supporting clinical trial activity', i.e. it excludes machines used exclusively for test and development, but includes all production machines and those used for immediate backup, e.g. mirrored or failover machines. Failure of a production machine is often obvious because the functionality suddenly disappears, but the centre also needs to be aware of 'silent failures' that may occur in a backup machine, and which may not become obvious until later - perhaps when that functionality is urgently required.

Evidence that the standard has been met includes:

a) inspecting the server monitoring system(s);

b) looking at examples of any past alerts;

c) interviewing staff.

If the centre does not manage its servers itself it would still be expected to have the relevant evidence available during an audit, obtained from the organisation responsible for that management.

IT02.05 Controlled environment

Servers should be housed in a temperature controlled environment

Servers require controlled conditions of temperature and humidity for optimum functioning and any server room should at least be able to maintain temperatures within a defined range.

Evidence that the standard has been met comes from inspecting the server room system(s) or the specification / accreditation of the server room's features.

If the centre does not manage its servers itself it would still be expected to have the relevant evidence available during an audit, obtained from the organisation responsible for that management.

IT02.06 Hazard control - fire alarms

The server room should be fitted with heat and smoke alarms, monitored 24/7

Servers must be protected from fire, hence this requirement.

Evidence that the standard has been met comes from inspecting the server room system(s) or the specification / accreditation of the server room's features.

If the centre does not manage its servers itself it would still be expected to have the relevant evidence available during an audit, obtained from the organisation responsible for that management.

Other aspects of environmental and system control

Over and above the requirements listed within the standards, there are a range of other features of environmental control that a centre could introduce, or pay for if their servers were hosted by another organisation, which are indicative of good practice in this area. These include:

- An alternative power supply, e.g. from a local generator, to allow continued functioning during a lengthy power loss (UPS systems are usually designed only to last long enough for a managed shutdown).
- An automatic 24/7 intruder alarm system, providing alerts remotely (to security staff and / or senior IT staff) if triggered.
- Automatic 24/7 server monitoring, (rather than the business hours requirement of IT02.04) with alerts being sent 24/7 to relevant personnel, so that failures can be picked up in the evenings and over weekends and national holidays.
- Success / fail messaging built into scheduled jobs, using for instance the messaging capabilities of PowerShell on a Windows server, or the built in email services in a modern DBMS. This augments the hardware monitoring

provided by server monitoring systems, and provides useful assurance that functionality is continuing as planned. Suddenly discovering that a nightly file transfer process has not worked for the last two months can be both embarrassing and costly!

- Full HVAC (heating, ventilation, and air conditioning) control systems installed in server rooms. These are usually found in commercial and dedicated server hosting environments, but may not be available where premises originally designed for other purposes are used to house servers and related equipment.

- Automatic fire response measures (e.g. inert gas or a misting system) as well as fire alarms.

- Protection against include water ingress (e.g. from external flooding or a burst pipes)

- Protection against infestation with insects or rodents

IT03 Logical Security

The standards in this section cover protecting data from unauthorised access, from outside the data centre (controlling and differentiating access from within the centre is dealt with in IT04).

Variations between systems and the constantly changing nature of security threats mean that it is difficult to stipulate specific security measures for systems. What is essential, however, is an ongoing review of security risks, security mechanisms and incidents (hence IT03.01) as well as general commitment to the principles of data protection and access control (as illustrated by the other standards in the section).

IT03.01 Security management system

Regular reviews of security (practices, incident analysis, risk assessment, documentation etc.) should occur across all IT systems relevant to clinical trials activity, followed by any necessary corrective and preventative actions.

This standard is equivalent to implementing a basic Information Security Management System (ISMS). The term is borrowed the ISO27001 standard on Information Security Management, though there is *no* expectation that that the centre or its parent organisation has obtained or is seeking full ISO27001 accreditation. The essential features of an ISMS are:

- Management commitment to information and system security, and the production of associated policy statements and controlled documents.
- Identification of security risks, together with an assessment of the potential damage to the centre from a failure in each case.
- Selection and implementation of security controls to reduce the identified risks and to meet the security objectives.
- Continued review and adjustment of security controls as circumstances change and incidents occur and are analysed

An ISMS ensures that "security controls are not merely specified and implemented as a one-off activity but are continually reviewed and adjusted to take account of changes in the security threats, vulnerabilities and impacts of information security failures…." (www.iso27001security.com/html/27001.html).

Evidence that this standard has been met would include:

a) controlled documents dealing with system security, that identify who is responsible for different aspects of security management;

b) minutes or other records of a periodic review process and any subsequent corrective or preventative action;

c) records of incident analysis and any subsequent corrective or preventative action;

d) interviews with staff to discuss how the system operates in practice.

This evidence may be available at the level of the data centre and / or the IT host organisation.

IT03.02 Commitment to data protection

The centre and its staff can demonstrate compliance with and commitment to all relevant data protection legislation, including the provision of related training programmes.

A key component of system security relates to data protection legislation and policies.

Here 'relevant data protection legislation' means that which applies in the countries where trials managed by the centre are carried out, not just the legislation of the centre's own country. For instance German and Danish data protection regulations would be relevant to a French centre if that centre was running a trial with centres in Germany and Denmark.

The expectation is that staff are made aware of their legal and ethical responsibilities under data protection, as part of their initial and continued training (whether carried out by the centre or external agencies). Controlled documents should also be available that demonstrates the centre's commitment to data protection and how they comply with relevant legislation.

One or more members of staff, in the centre or the parent organisation or both, should be identified as a 'data protection officer' and be available to provide both local support and guidance and advice to management, where necessary, on potential problems in complying with data protection legislation.

The evidence required to show that the standard has been met includes:

a) controlled documents that describe how the centre implements data protection policies and the responsibilities of members of staff under those policies;

b) One or more staff identified as having special responsibility for ensuring compliance to data protection legislation

c) records of training concerned with data protection (some level of training will be required for all IT / DM staff);

d) interviews with staff to check understanding of data protection requirements and discuss how the systems work in practice.

IT03.03 External firewalls

External firewalls should be in place and configured to block inappropriate access

A centre or (more normally) its host IT organisation should have external firewalls set up to block unauthorised access from outside the centre.

Exactly how the firewalls would need to be configured will depend on circumstances. A centre running eRDC, for instance, would normally have at least two such firewalls with any externally facing web server(s) placed in the logical 'space' between them, the so-called demilitarised zone or DMZ.

Centres providing non web based remote access, e.g. through VPN or Citrix, will need to configure their firewalls to support this.

Carrying out and documenting regular penetration testing is not part of the standard but it is seen as best practice. Such testing can be done by commercial organisations but in the non-commercial sector can also be done by arranging mutual testing between institutions.

It is also good practice to continually monitor traffic activity and to try and identify and investigate any hacking or denial of service attempts.

It would be up to the centre or IT host organisation staff to explain how the firewall configuration worked to block inappropriate access, and provide documentary evidence as appropriate - e.g. diagrams of the server / firewall systems, firewall settings reports, records of firewall penetration tests, failed access attempts, etc.

IT03.04 Encrypted transmission

Clinical data transmitted over the internet to or from the trials unit should be encrypted

All clinical data must be encrypted if transmitted to and from the centre over the internet, to prevent eavesdropping, tampering and 'man-in-the -middle' security attacks.

This will normally be in the context of eRDC, when the https protocol is used to encrypt transmitted information. It may also take place in the context of a VPN or Citrix connection. In the latter case the encryption should extend to the whole of the data transmission and not just the initial exchange of certificates.

Centre staff will need to explain how the systems they use support this and provide the documentary evidence as appropriate - perhaps taken from the vendor's / developer's specifications of the CDMS.

IT03.05 Server administrator roles

Administrative access on servers should be restricted to specified members of IT staff

Administrator level access to the centre's servers should be restricted to a small number of specified staff, usually IT staff within the centre and / or IT hosting organisation with particular responsibility for server management.

More senior staff within either the centre or the host IT organisation should not routinely have administrator level access unless they also have specific server management roles.

The evidence that this standard has been met would be the production of the current list of staff with this access plus justification for the access that has been given.

IT03.06 Administrator access management

Administrator access to servers should be subject to agreed access management policies

Administrator access must be subject to appropriate management policies, so that the security of the access can be maintained over time.

These policies would be locally agreed, but would normally be based on a strict password management regime, i.e. 'strong' passwords and enforced password change after a fixed period, though in some circumstances could include biometric or card reading technologies. Password change might also be necessary after key staff leave, especially if the leaving was not by choice.

Administrator or other elevated privilege accounts are often necessary for running services (or 'daemons' in UNIX environments). Such accounts should be set up and maintained separately from accounts assigned to individuals, which allows them to be managed separately.

From the point of view of business continuity it is a good idea to have some key administrative passwords stored off site (traditionally in a sealed envelope in a safe). This can conflict, however, with the need to periodically change these passwords to ensure that they are not compromised. There is no easy answer to this problem, though using a secure cloud based 'password locker' may work in some cases, as long as it is kept up to date.

Evidence that the standard had been met would normally be obtained from:

a) controlled documents detailing access management policies as they apply to administrator level accounts;

b) interviewing staff - it would be up to centre staff to explain how the policy provided an effective barrier to administrator access being compromised.

IT03.07 Internal firewalls

Inappropriate access to clinical trials data from elsewhere in the organisation should be blocked, e.g. by correctly configured internal firewalls.

Most centres are a part of a larger parent organisation, especially with regards to IT infrastructure. When this is the case there is a need to ensure separation from other users in the organisation, or at least strictly control the allowed traffic.

For a university, there is a particular need to block accidental or deliberate attempted access by student users, whilst for a hospital there is a need to prevent any unauthorised access into hospital systems from the centre, as well as vice versa.

The commonest method to block access in this way is probably by using internal firewalls between different parts of the network, but other forms of access control (e.g. domain and user group management) may be used instead of or in addition to firewalls.

The evidence that the standard has been met will include

a) relevant controlled documents describing how access is managed and / or blocked

b) interviews with staff with reference to maps of the network's logical structure, explaining how the access control systems work in practice.

IT04 Logical Access

The standards in this section cover the control and differentiation of access from within the centre (protecting data from unauthorised access from outside the data centre is dealt with in IT03).

The access being considered is to the data centre's own network and to 'all systems directly supporting clinical trial activity'. This most obviously includes the CDMS, but will also include (for instance) treatment allocation and trial administration systems. It excludes systems used exclusively for development, testing and training.

IT04.01 Logical access procedures

Controlled documents covering access control to all systems directly supporting clinical trial activity should be in place

This standard simply requires that controlled documents exist that govern access management, both to the network, which acts as the initial portal, and then to systems involved in directly supporting clinical trial activity.

Network access is often managed by the centre's host organisation, while the centre would normally manage access to its own systems. There will therefore often be two sets of controlled documents.

The evidence will be the documents themselves, which should include a summary of responsibilities, processes, outcomes and documentation involved in controlling logical access.

IT04.02 Access control management

Each system requiring access controls should have mechanisms, e.g. using roles, group membership, etc., that can be used to effectively differentiate and manage access

This standard requests that sufficient mechanisms exist to provide differential access, in terms of both allowed functionality and data. This might be by role assignment in a CDMS, or by explicit allocation of rights within a file management system, and would normally be done through managing group membership rather than on an individual basis.

The standard is concerned with 'each system requiring access controls', starting with the initial log-in to the centre's / parent organisation's network for internal staff, and including access to the CDMS for both internal and remote eRDC staff, but also including any other systems where access control would be expected because they directly support clinical trials activity.

Evidence that the standard has been met includes:

a) controlled documents detailing how access control is implemented;

b) direct demonstration of access control mechanisms and inspection of systems, especially log-in processes.

IT04.03 Granularity of access

Access control mechanisms should be granular enough to allow compliance with the data centre's own policies on access control

This standard (which in practice would probably be considered together with IT04.02) emphasises the need to support granular access, i.e. to allow fine control over the access provided and the functionality provided with it, to different datasets and for different roles.

Granularity clearly applies to remote eRDC staff, who should only ever see their 'own' site's data, but it also applies within the centre, where staff should not be able to see data or other files that are sensitive scientifically, e.g. randomisation lists, or clinically / commercially, e.g. analysis results, unless they have a genuine need to do so.

Granularity may also be found in fine control over access to clinical data - for example a member of staff who works on one study should be able to see and edit the data for that study; her manager might be able to view that data but not edit it; a monitor might be able to raise and close queries for that study but not enter data, etc.

The granularity required should match the centre's policies on access control, themselves driven by the organisation of staff, tasks and systems.

Centres that store more obvious PID (e.g. patient names and addresses used to contact trial subjects in quality of life studies) will usually need to provide greater granularity of access, to protect that data, than centres that do not (or are not allowed to because of local data protection legislation).

Evidence that the standard has been met includes:

a) controlled documents detailing how access control is implemented;

b) direct demonstration of access control mechanisms and inspection of systems, especially with regard to particularly sensitive data types;

c) discussions with staff about how and why the necessary granularity is supported.

IT04.04 Network log-in management

Network log-in management should be enforced on all users, usually including regular change and / or complexity rules for the log-in password

Protecting initial entry to the network for centre staff is a key part of managing access. Normally a process is established that enforces 'strong' passwords and a change after a fixed period (e.g. 90 days), but in some centres biometric devices or personal cards may be used, instead of or in combination with passwords ('2-factor authentication').

Evidence that this standard was met would come from:

a) Controlled documents detailing the management policies for network log-in;

b) Proformas and other documentation, and / or demonstration showing those policies being used;

c) Discussion with centre staff about how the local network log-in policy worked.

IT04.05 Remote access

Remote access should be controlled using the same principles as local access control, and should not normally include access to the host's network (unless the user has a pre-existing identity on that network).

Remote access is used here to mean direct access to a server and specific applications and / or the centre's network, e.g. using Citrix or VPN, rather than the browser mediated access of an eRDC system to data entry screens.

It may be provided for centre staff, who will usually have their own identity on the local network (for instance a monitor when working away from the centre) or for staff who are completely external to the centre, perhaps working for a collaborating organisation.

Remote access management should reflect this. It should prevent external users from gaining access to anything other than the specific applications and datasets that they have been authorised to use, and in particular prevent access through to the host's network. Internal employees may, in some systems, enjoy the same access as they would have if they logged in locally (more often a sub-set), and the remote access mechanisms should be able to manage this effectively.

Evidence for this can be obtained from:

a) relevant controlled documents;

b) from interviews discussing how any remote access is managed;

c) demonstration of the remote access system's access control mechanisms and records, including relevant proformas.

IT04.06 Network lockout

Logins to the network should be locked after a locally determined inactivity period, requiring secured re-activation

When an employee moves away from their machines while logged into the network and / or a particular system, there is a risk that another user may use that machine, 'hijack' their access rights and gain unauthorised entry to systems. There should therefore be an *automatic* mechanism that locks the screen and which requires a password or equivalent mechanism to unlock.

The mechanism must be automatic after a pre-set time- not normally more than 15 minutes.

Requesting that users lock their machines manually does not provide a sufficient guarantee that it will actually happen, though those with particularly high access rights, such as senior staff, may be advised to lock their machines manually before the automatic time-out is triggered. The lock-out should apply to the network log-in and therefore lock the whole machine. Many CDMSs also provide an automatic log-out mechanism but on its own this is insufficient.

Evidence for this can be most easily obtained from direct observation, backed up by interviews with staff.

IT04.07 Administration of access to clinical data

Access rights to systems storing or processing clinical data should be regularly reviewed, changes to access requested and actioned according to defined procedures, with records kept of all rights, when granted, why and by whom.

This standard deals with the administration of access to clinical data systems. It requires that a system is in place to request and implement changes, to record when access rights were changed and by whom and that the rights are reviewed periodically (at least annually) to ensure that they are all still required.

Periodic review is particularly important for remote users, who are often employed by other organisations, and who may therefore leave without the data centre being made aware that they can drop access.

The standard only applies to those systems dealing with clinical data, but it would be good practice to extend the requirement and record *all* access requests / changes, including to the network and other (e.g. trial administration) systems.

Evidence that the standard has been met should come from:

a) the relevant controlled documents;

b) examples of the request and review procedures;

c) the records maintained within the system itself.

IT05 Business Continuity

Business Continuity (BC) is the set of activities performed by an organisation to ensure that critical business functions will remain available to staff, customers, suppliers, regulators (etc.) after a major loss of function. The loss may be caused by a natural disaster (flood, fire, earthquake, hurricane, etc.) or be man-made (e.g. sabotage, walkouts) or be as simple as the sudden loss of key staff.

BC is *not* restricted to IT systems! It can include communicating with clients, storing copies of key material off-site, arranging alternative premises, hiring consultants or temporary staff and finding alternative service suppliers. The IT component of BC is Disaster Recovery (DR): the process of recovery or continuation of IT systems after a massive loss of functionality.

DR may include rebuilding and / or restoring data for applications, and re-establishing hardware, communications and other IT infrastructure. Key to any disaster recovery policy is the retention of copies of data, but so also is keeping copies of other key information (passwords, activation keys, scheduled jobs, user information etc.).

This section deals with business continuity in general (IT05.01) though the rest of the standards are focused on IT disaster recovery.

IT05.01 Business continuity plan

The centre should have or be developing a Business Continuity Plan, covering likely action in the event of a major loss of function (e.g. fire, long term power failure, full server failure, sudden loss of key staff).

It is recognised that a BCP can take a relatively long time to implement properly, not only because additional funding may be required, but also because much has to be done in conjunction with the parent organisation. The current standard therefore requires that the centre has, or as a minimum is developing, such a plan.

Because a BCP is seen as an important requirement for ECRIN certified data centres this standard is likely to change in a few years, so that centres will be required to have a *completed plan in plac*e. The requirement will also probably include the need for that BCP to be reviewed regularly, at least annually, in conjunction with the parent organisation.

The evidence required is the BCP document itself, or documents that show that such a plan is in current development.

IT05.02 Back up policies

Controlled documents detailing backup policy, procedures, restores and testing should be in place

This standard requires that there are controlled documents that detail the backup procedures.

Even if the IT host organisation is entirely responsible for the implementation of backups and restores, the data centre would still be expected to have policies regarding the type and frequency of backups and the restore tests that it requires. That policy would then be incorporated into agreements between the centre and the host IT organisation.

The evidence would be the controlled documents themselves.

IT05.03 Back up frequency

Backups must be taken using a managed, documented regime that ensures that new or changed data is backed up within 24 hours, and which allows the centre to check that the system is operating properly.

This standard on back up frequency reflects the fact that back up regimes are usually sophisticated enough to identify and only process data that actually needs backup because it has been changed or newly inserted.

If a centre is managing their own data backups it is relatively straightforward to monitor that the process is operating properly. If backups are the responsibility of the IT host organisation the centre still needs to assure itself (e.g. by receiving reports or periodic copies of the logs) that the backup process is operating properly.

The evidence that the standard has been met includes:

a) documentation describing the backup regime and how it is managed, either from the data centre or the IT host organisation;

b) logs of the backup process and / or periodic summary reports indicating the backups are proceeding as required.

IT05.04 Back up storage

Back up media storage (location, protection, redundancy) should be sufficient to avoid data loss if there is a fire or other disaster

Simply backing up data does not guarantee that it will survive a large scale disaster such as a fire, especially if it remains in the same location as the original data.

A variety of mechanisms exist to ensure that a such a disaster will not wipe out data, for instance secured off-site storage of tapes, on site storage in fire-proof safes, duplication of back up data to a mirrored site, and twinned but physically

separate backup systems (e.g. at opposite ends of a large university or hospital campus)

This standard requires that one of these mechanisms, or something equally effective, is in place to ensure that if a large scale disaster happens at one of the data storage sites a copy of the data is still available.

The evidence that the standard was being met would come from:

a) controlled documents describing the procedures for storage of backups and the systems supporting this.

b) discussion with staff to clarify procedures and explore how the systems work in practice.

IT05.05 Back up - Environment

Any necessary data management / administration data (access groups, log-ins, scheduled jobs etc.) should be backed up and restorable

Though the retention of copies of data is necessary for disaster recovery, so also is keeping copies of other critical information (passwords, activation keys, scheduled jobs, user information etc.).

This is particularly important for database systems, where the database server may hold a great deal of data management / administration information. This may or may not be backed up automatically by the IT host organisation's systems, and so may require additional agreements or scripts being run by the centre staff. (The same sort of data is also necessary for file based systems but this is usually backed up along with all the other file material).

Evidence that the standard had been met would come from:

a) relevant controlled documents;

b) interviews with staff, including explanations and demonstration of the backup / restore mechanisms used.

IT05.06 Recovery Testing

Testing of full restore or failover procedures, should take place and be documented at a frequency that reflects system and staff changes (for all servers relevant to clinical trial activity)

Back up is of little use without corresponding mechanisms for restoring data, and those restore mechanisms should be tested. With single or small groups of files this is rarely problematic, but it is more difficult when the need is to rebuild a whole server back to the state prior to failure, or to that of the night before, from the bare machine (a 'full restore').

It is therefore necessary to carry out and document periodic test restores of whole servers to ensure that the proposed methods actually work. Ideally these would involve less experienced staff rather than the senior staff who probably set the system up initially. There is no guarantee that any particular staff members will be present if and when a restore is required for real.

Relatively small and simple servers may be straightforward to restore using re-application of machine snapshots. Virtualisation can make this even easier, by simply moving a server profile to another hardware base, though such mechanisms still require full testing. Tests of full restores on a large database or file server can take time to set up and will normally involve spare hardware.

For database based systems, mirrored servers or data duplication (using scheduled replication or transaction log shipping) allows a much more rapid failover if failure occurs and is generally regarded as good practice. It does, however, carry an additional administrative overhead as well as demanding additional hardware.

In these circumstances 'restore' and its testing usually involves a failover process, but may still include renaming servers or changing IP addresses to ensure that applications point to the right systems.

The optimum frequency of restore / failover tests will depend on circumstances: there is little point in running through a restore process if nothing has changed since the last test, but major changes in the system will almost certainly demand a revision of the restore procedure that in turn requires retesting.

There is also a need to ensure that new IT staff are familiar with the procedure if they may be asked to carry it out unassisted by more experienced staff. The standard as written reflects these requirements. Although the standard does not specify a fixed period between tests a full recovery test should probably occur at least every two years for any server directly supporting clinical data activity.

It will be up to centre staff to describe and justify their methods for recovery testing and its frequency, and show that they have assured themselves that they could safely restore or failover any of their production servers to normal operation within the period stipulated by their own controlled documentation.

Testing recovery is easier to organise if the centre manages all of its own IT. If it is the responsibility of the IT host organisation, than the centre still needs to assure itself that recovery testing is occurring and is successful, and should build this into the service agreements with the host organisation. In practice, even if the host IT organisation takes the main responsibility for recovery testing, they will probably have to liaise with the centre to carry it out effectively.

IT06 General System Validation

As used within the ECRIN standards and related material, 'validation' refers to the process of ensuring and documenting that a system is functioning as intended. Note that a 'system' will normally involve hardware, software, people and processes - i.e. it does not just include the IT components - and validation should reflect that.

This section looks at validation in general, of all systems used by the data centre. There are additional specific aspects of validating trial specific database systems (CDMAs) but these are covered in section DM02.

The complexity of systems and their usage means that absolute validation, i.e. of all possible inputs and situations, is almost always impossible. To quote the FDA, validation is:

*Establishing documented evidence that provides **a high degree of assurance** that a specific process will consistently produce a product meeting its pre-determined specifications and quality attributes."*

(FDA, Guideline on General Principles of Process May 1987, emboldening added).

Judgements therefore need to be made on the level of evidence required to show, with 'a high degree of assurance', that any particular system is functioning properly. Those judgements should be based on the potential risks of system malfunction, to the validity, integrity, availability and security of the data, and ultimately to patient safety, as well as the practicality and costs of the validation methods used.

In the latest version of the GAMP framework (GAMP 5, 2008), 'validation' has largely been replaced by 'verification', defined as the confirmation that specifications have been met. Most data centres and their staff, however, are more familiar with validation and the associated terminology from the V-model of GAMP 4 - i.e. initial, operational and performance qualification. For the moment therefore the ECRIN standards and related material have retained the GAMP 4 terminology.

The three types of qualification are defined below:

- *IQ (Installation Qualification*): checks that a system's installation is correct with respect to the vendor's (design) specifications - i.e. everything is in the right place and the various components / modules are interconnected properly and can be accessed as required.

  IQ is the normal initial step in validating systems. In practice IQ scripts usually check installation by verifying a core sample of functionality, that confirms that all components in the system are accessible and available.

- *OQ (Operational Qualification)* checks that a system is functioning correctly, i.e. against the system's functional specification for commercial systems or the design team's specification for local systems.

  In practice this means establishing, documenting and running through a series of test cases, often supplied for commercial systems by the vendor as an OQ script, that examines each aspect of the claimed functionality. OQ for a major system like a CDMS may take several weeks.

- *PQ (Performance Qualification)* is the process of checking that the system, over a range of 'real world' conditions, continues to perform as intended.

  PQ is an important additional stage because OQ, especially if only using a vendor supplied list of test cases, may not fully reflect the intended usage. It is one thing to confirm that a module works as advertised with 1 user and 20 patients, quite another to check that performance is still acceptable with 50 users and 5000 patients, or to discover that intrinsic limits prevent work with populations (of data items, subjects, logic checks etc.) greater than a certain size.

In practice PQ can often be partly integrated with OQ by designing additional test cases with realistic loads. The context of PQ should also mimic, as far as is possible, actual usage - in particular real users should be involved in some aspects of the testing process. In other words PQ should include some User Acceptance Testing (UAT).

The balance between OQ, PQ and the sign off into production use is another risk based decision process. In low risk scenarios it might be OK to start to use a system after successful OQ, after which the system would be tested / monitored against a steadily accumulating range of real usage conditions. In higher risk scenarios some PQ / UAT will usually be done as well, with users being given access to the system, deployed as it would be for production use, and asked to run additional tests.

There is always a balance between the time and resources spent on validation and the risks involved in not confirming a system's functionality in different scenarios.

The results of validation are the basis of the decision to accept or reject a system for production use, but there is not always an automatic simple link between the two processes. In other words validation - i.e. 'does this system appear to work as advertised?' is different from the acceptance decision - 'does this system work well enough for us to use it?'. The second question demands a risk based decision based on the answers to the first.

For instance even if a system fails some components of its OQ / PQ testing it still may be acceptable for use if the problems are not critical (i.e. do not affect GCP and regulatory compliance), or a workaround is available, or the system vendor / designer can be persuaded to quickly add or fix the missing functionality. The reality is that the time and money spent on assessing and procuring a system, or

building one in-house, and then installing and validating it, are usually far too high for a non-commercial data centre to be able to quickly switch to another system.

Validation is therefore more complex than a simple test that must be 'passed' before a system can be used - it is more about completely understanding a system and discovering its limitations as well as having confidence in the core functionality.

Validation is also an *ongoing* process. Systems change (are upgraded, reconfigured etc.) and so do the loads and requirements placed upon them. The technical environment (e.g. other systems added, new physical infrastructure) may change, and the organisational context may also evolve (e.g. new procedures and staff).

All such changes present possible new risks to data validity and integrity, and thus patient safety, and so re-validation should be considered as part of any change management process.

At some point there may be less risk involved in retiring and / or replacing a system than trying to continue to use it. Ongoing validation and risk based assessment should therefore be seen as the basis of deployment and usage decisions throughout the life cycle of any system, and not as something limited to initial installation and deployment.

IT06.01 Validation policies

Controlled documents should be in place covering system validation approaches, responsibilities and processes

This standard requires the centre to have developed controlled documents that describe a validation strategy. This should describe the *general* approach(es) to be taken, the responsibilities of different roles within the centre, and the expected outputs, the whole approach being integrated within the centre's overall Quality Management System.

The documents may include reference to particular frameworks and models for validation and risk assessment (e.g. GAMP, PIC/S) but there is no expectation that any particular framework should be used - partly because those frameworks are themselves evolving, partly because most have their origins in the pharmaceutical industry, and often in manufacturing and laboratory practice rather than the specific validation requirements of data management systems.

Such frameworks can certainly be very useful, but work better as a starting point for developing local ideas and systems than being 'dropped in' as complete, fully formed solutions.

The evidence would be the relevant controlled documents.

IT06.02 Validation system inventory

The centre should have system inventory documentation, identifying all IT systems relevant to clinical trials activity, the risks associated with each, and - in summary - the consequent validation strategy for each

Validation should be considered for *all* systems in the centre, and not just the obvious ones like the CDMS and the individual CDMAs. The risk assessment and validation decisions about each system should be documented and justified, even if the decision is not to do any local validation (for instance most centres would not try to validate server operating systems, assuming this had been done by the vendors and their beta-testing, as well as the huge numbers of previous users).

The documentation should identify the risks associated with each system, the types and level of testing required, and normally who will be responsible for it, when, and why, what tools they will use, the nature of the outputs of the validation process and the nature - in broad terms - of the *revalidation* policy with regard to system upgrades and patches. The key requirement is that validation requirements have been carefully considered and match the risks associated with each system.

These validation management decisions are often collected together as a '*Validation Master Plan*' though that term can mean different things to different people. The requirement is that the documentation exists, though it need not be a single document with a particular title, hence the use in the standard of 'system inventory documentation'.

IT06.03 Risk based approach to Validation

The general approach to validation of any system should be based on analysis of potential risk, and take into account the system's usage, users and origins

The expectation is that the validation strategy will be based on risk assessment. Factors that can influence risk assessment, for systems and the components within them, include:

- *The potential impact of malfunction:* A component that contributes to data integrity, or GCP or other regulatory compliance, or is otherwise involved in maintaining patient safety, clearly has a higher potential impact - on patients, the scientific conduct of the trial and the reputation of the data centre - if it operates incorrectly than (for instance) a module allowing users to easily reset their own passwords or a report that gives a breakdown of accrual figures by site / month.

- *The possibility of silent failure:* Some problems in systems are obvious as soon as they appear. They will disrupt work but are unlikely to be allowed to impact the study's results in the longer term because they will be resolved. Other problems are less obvious and may introduce errors without the users being aware of the problem until much later. The costs of resolving the problem, and the potential impact of the issue, are correspondingly greater.

- *The numbers of other users:* Though systems should always be validated in their own local environment, systems developed by established vendors and in common usage (e.g. operating systems, DBMSs) will normally carry less risk than specialist, often locally configured systems (e.g. CDMSs). Systems with a large user base are extensively tested by their vendors, and there will also be a user community that can identify and publicise potential issues.

- *The resources used to develop the system:* Systems that are developed by companies with extensive development resources, and well established quality management practices themselves, are likely to carry less risk than systems created by new and / or small development teams, and especially by a very small in-house development team - often one person. (On the other hand the responsiveness of the development team in fixing identified problems often varies in the opposite direction).

The amount and types of testing required for validation, e.g. the amount of additional performance qualification over and above vendor supplied OQ scripts, the amount and type of user acceptance testing, should vary with the risks associated with any system, taking into account the factors listed above and any others that appear relevant.

Other elements of risk based validation management include the processes established to manage change management in a system.
The evidence that this standard has been met would largely be found in the validation system inventory (see IT06.02), as well as in discussion with centre staff, explaining how risk assessment was applied in practice.

IT06.04 Validation Detailed Evidence

Detailed validation documents should exist for any particular system, detailing the validation carried out, including any test data and protocols, and the results obtained

Each system validation exercise should generate a set of retained detailed validation evidence - i.e. the descriptions of the tests and their results. The documents should also indicate who carried out the tests and when. In some cases these may be electronic rather than paper documents.

A system that failed an initial validation exercise would normally then have further documents listing the actions taken to remedy the problems identified, and then further detailed validation documents covering the aspects of the system that were retested.

The evidence for compliance would be the documents themselves, against a range of different systems.

IT06.05 Validation Summaries

A signed and dated summary of the results of each validation should exist

As well as the detailed results (see IT06.04) any validation exercise should also generate a relatively short summary (often one page) of the validation, signed off and dated by one or more key staff, that confirms that validation has been completed and which indicates its result.

A system that failed an initial validation exercise would normally then have a further summary statement after actions had been taken to remedy the problems identified, indicating the results of the second validation.

The evidence for compliance would be the summary statements themselves, against a range of different systems.

IT06.06 Change Management Policies

Controlled documents should be in place defining change management mechanisms and their scope, who should authorise and review requests, and how they should be documented

All systems are subject to change, for instance from user requests or vendor upgrades and patches, and those changes should be managed for systems to retain their validation status.

This standard requires that there are controlled documents that specify the change management process and procedures, as well as the roles and responsibilities involved, and how it is documented. Such documents are often augmented by sample proformas for requesting and signing off changes.

The evidence would be the documents themselves.

IT06.07 Change and risk evaluation

Change management in relation to systems that support clinical trial activities should include a documented risk evaluation (including a review of the need for revalidation) and a record of the consequent decision and actions.

As with initial validation the basis of decision making in change management should be a risk analysis. Some questions include:

- How critical is the functionality being changed?
- Who will be affected by the change and in what context?
- What are the possible impacts on other aspects of the centre's functioning?
- Will documentation and / or training need to be revised to reflect the change?

The response to the first question in particular will dictate how much re-validation of the relevant parts of the system will be required.

Many centres use a 'check-list' approach to change management that allows these, and any other issues identified as relevant, to be considered in a structured way and the decisions taken in respect of each to be easily documented.

The evidence that the standard is being met would be the change management documentation illustrating that risk based evaluations are taking place and describing the decisions taken as a consequence.

IT06.08 Change and evidence of re-validation

Any re-validation associated with a change, of the entire system or parts of it, should be planned, executed and documented as part of the change management process

This standard requires that when re-validation takes place as part of the change management process it is integrated within that process, and not simply added on afterwards to a change that has already happened.

The re-validation would normally generate detailed documentation that would indicate if the relevant parts of the system still functioned as intended, or not, plus a signed and dated summary statement to that effect. If the re-validation is successful that part of the 'change management loop' can be closed. If not the change will need to be reversed or revised and the change management process reset.

Evidence that the standard has been met would be:

a) change management documentation that clearly reflected this method of working;

b) structures (e.g. test systems in which changes can be rehearsed) that supported it in practice;

c) discussions with staff to clarify how the systems worked in practice.

IT07 Local Software Development

This section provides three standards dealing with local development of software and systems. The scope *excludes* statistical scripts generated during analysis, but includes all other types of code and system development, for instance of utilities supporting the CDMS or reporting, procedures within databases, and trial administration, coding and treatment allocation systems. In some centres the CDMS itself may have been developed locally.

These systems are subject to the same validation assessment and requirements as any other system but they also have specific requirements relating to their development.

It is recognised that many centres do not carry out any local software development (apart from creating statistical scripts during analysis). These standards therefore only apply to those centres and systems where local development occurs.

Two of the three standards (IT07.01 and IT07.02) relate to documentation, and are designed to ensure that a centre is not leaving itself exposed if staff were to leave, a common problem with local software development.

IT07.01 Documentation of in-house software

System documentation should cover system architecture, plus identification of individual modules / classes and their inputs, outputs, and purpose

The focus of this requirement is a top-down overview of the system and its architecture, including a brief description of each module and constituent class - purpose, inputs and outputs.

The level of documentation should be sufficient - when used with the in-line commenting described in IT07.02 - for another competent developer to make sense of the program and start work on it in a reasonably short time.

The evidence would be obtained from examining the relevant documentation. The judgement is necessarily a subjective one but worth attempting because of the importance of documentation in supporting any software project.

IT07.02 In line Commenting

All code should have sufficient in line documentation to support tracing of program execution

The focus of this particular requirement is bottom-up in-line commenting, so that program execution, particularly when it involves non-obvious algorithms, is adequately described.

The level of documentation should be sufficient that - when used with the overview documentation described in IT07.01 - another competent developer could make sense of the program and start work on it in a reasonably short time.

The evidence would be obtained from examining the relevant code. The judgement is necessarily a subjective one but worth attempting because of the importance of documentation in supporting any software project.

IT07.03 Software development

The software development methodologies used can support quality assurance techniques and promote ease of future maintenance

This standard deals with the need to assure that the methods used in local development can support QA processes and current 'good practice' - in particular that the systems are likely to be flexible and easier to maintain because they are well compartmentalised, demonstrating what is known as 'separation of concerns'.

Within such systems distinct functions are handled by distinct components - modules that in theory can be swapped in and out, as long as their replacements provide the same interface to the rest of the system. The MVC design paradigm for web based applications is an example of a methodology specifically designed to support separation of concerns.

Other aspects of software development that would indicate good quality (other than good documentation) include:

- Use of a source control system that allows branching and release management
- Programming against interfaces rather than concrete fixed components
- Programming against data repositories rather than fixed data sources
- Use of a unit testing framework and / or integration tests
- Use of a library of user controls / common modules across systems
- Code reviews and walk-throughs; shared coding

The expectation is not that all of these techniques would be used - not all of them are applicable to all types of development and they can be difficult (and time consuming) to retro-fit to existing systems and patterns of work.

There would be an expectation, however, that in-house developers were aware of these approaches, and could explain why they had implemented some and not others. The judgement of the auditors would necessarily be subjective, but would focus on current development approaches and discussion with developers.

IT08 Extracting and Reporting Data

These four standards are all concerned with exporting data from the CDMS, as formatted reports or files, either directly or involving further data processing.

In one sense they are all redundant - access control for reports is just one case of general access control, while validating reports, extracts and data transformations are all special cases of general validation.

They have been brought out as separate standards, however, because rigorous report / export management can be difficult because:

- the range of reports available in many systems, and the fact that these can often be parameterised, leads to a very wide range of reports / data extracts to consider and test, and
- user designed reports and extracts are usually added gradually to a system over time, and may 'slip through' normal control and validation procedures.

The three standards on validation - IT08.02, IT08.03, IT08.04 - are very similar and may overlap in practice, because report, data extraction and data transformation themselves often overlap in practice. They may therefore be audited together.

IT08.01 Report access control

Access to reports should be controlled and match the users' requirements as well as the relevant regulations and laws.

Access to reports (and data extractions), like access to any other data in the system, should be controlled, i.e. users should only see the data that they have a right to see and be able to run the reports that are relevant to their role within the system.

Evidence that the standard has been met would come from:

a) controlled documents describing how access to reports and data extractions was controlled;

b) demonstration of the control mechanisms;

c) discussion with staff to clarify how the system worked in practice.

IT08.02 Report validation

The structure and accuracy of reports should be validated.

As with other validation tasks, the basis has to be a risk assessment. Relevant questions might include:

- How are the reports used? Are they providing critical clinical data (e.g. SUSAR details), quality management data (e.g. query rates by site) or administrative details (accrual figures)? The risks associated with report usage will probably be the major factor in determining validation requirements.

- How have the reports been constructed? Are they standard reports built in to the system and used (and therefore checked) by a wide variety of users, or are they ad hoc reports only available at a single centre, and perhaps only used by a few individuals at that centre?

- How complex are the reports? Are they simple listings or do they contain complex derivations and sub-totals?

- How easy are they to cross check? Would errors be obvious, e.g. by visual cross checking with the data in the databases or with data from other sources, or could errors slip through if not checked in detail?

Many reports can be parameterised, so part of the validation process will be deciding what range of parameters should be checked.

Although reports built into a system will often be validated as part of that system's validation, reports that are constructed on an ad hoc basis later may slip through normal validation procedures. It is important that such ad hoc reporting is also validated.

As with all validation, results should be documented and available for inspection. The validation documents would then form the evidence that the standard had been met.

IT08.03 Validating extractions

Any data extraction process should be validated

As with other validation tasks, the basis has to be a risk assessment. Relevant questions might include:

- How are these extractions used? Are they part of the process of providing data for analysis, or do they provide 'only' quality management or administrative data? The risks associated with the usage of the data will probably be the major factor in determining validation requirements.

- How have the extractions been created? Are they standard exports built in to the system and used (and therefore checked) by a wide variety of users, or are they ad hoc extractions only available at a single centre, and perhaps only used by a few individuals at that centre?

It will be important to consider the full range of data exports that are available to the centre, including those constructed on an ad hoc basis. Often exports can be parameterised, so part of the validation process will be deciding what range of parameters should be checked.

As with all validation, results should be documented and available for inspection. The validation documents would then form the evidence that the standard had been met.

IT08.04 Validating transformations

Any data transformation process should be validated

Reports and data extractions often include data transformations when they are generated, but such transformations can also occur in isolation, for instance changing the format of extracted data (e.g. from XML to SAS), or in preparing data prior to importing it into the system (e.g. into CSV files ordered in particular ways).

A particularly common and important transformation is that between the 1 row / data item structure used internally by many CDMSs and the 1 row / subject-visit, or 1 row / subject-domain structures preferred by many statisticians (and the FDA). Such a transformation may be built into the CDMS or it may be constructed in-house - either way if it is used it requires extensive validation as it forms a critical part of the data processing chain.

As with other validation tasks, the process should start with a risk assessment, focusing on the process(es) in which a transformation is used, and how critical those processes are to the overall scientific and data management of a study. When transformations can be parameterised it is also important to consider what range of parameters should be checked.

As with all validation, results should be documented and available for inspection. The validation documents would then form the evidence that the standard had been met.

Useful Reporting / Extraction capability

The ECRIN standards do not require any specific reports or report types to be available. Nevertheless the availability of a core set of reporting / extraction functionality, if properly managed and validated, can help to increase confidence that the centre possesses a robust and mature management system for reporting data from their systems.

This functionality, which should be seen as indicative of good practice, could include:

- A good range of standardised reports
  A set of frequently required (and parameterised) standard reports, available to appropriate users. Examples include accrual reports, by site and in total, missing data reports by site, query numbers / proportions and query status reports (e.g. by site).

These reports can be particularly useful for monitors wishing to identify sites which may be experiencing problems in supporting the trial. They may come from the CDMS or from other systems maintained by the centre (e.g. for trial administration).

- UI Ad Hoc Reports
  Ideally it should be possible to extract ad-hoc filtered reports via the UI, for instance with specific data items / forms / visits selected for a selected sub set of subjects.

- Optional Inclusion of Audit Data
  Selected and or ad / hoc reports should include the option of including audit data

- Report Save and Rerun
  Once a report is parameterised by a user it should be possible to save and rerun it manually.

- Automatic Report Generation
  It should be possible to automate and schedule the generation of reports, ideally also automating emailing them to selected users.

- Optional Inclusion of Metadata
  The option should exist to include a metadata description of data items within reports.

- Study-definition export
  Standard reports should include one to export the current study definition (for instance for re-import to another server). Ideally this would be structured using a standard system such as CDISC ODM.

- Single Subject report
  All the data received for a single subject should be extractable / reportable, ideally in a form that allowed easy comparison with source data, for ease of possible audit.

- Data by Input Personnel
  It should be possible to examine and export a record of a single data entry staff member's input data, for instance to identify the source of errors or training needs.

- Key Field Changes
  It should be possible to examine and export a full list of changes to identified key fields, e.g. fields reporting toxicity, to help support data monitoring.

DM01 CDMAs - Design and Development

A CDMA, or Clinical Data Management Application, is a system supporting data entry and management *for a specific trial*. It includes the databases and files used to store the data and associated notes and queries. It also includes the CRFs (paper and / or electronic) used for data entry and the trial specific data validation checks, skipping logic and derivations that those CRFs contain.

The standards in this section deal with how CDMAs and CRFs are specified and constructed, and how CDMAs in development are isolated from those in production. In addition, several examples of 'best practice', that can make CDMA development and CRF design quicker and easier, but which do not form part of the formal ECRIN requirements, are listed at the end of the section.

DM01.01 CDMA development policies

Controlled documents covering the development of CDMAs and CRFs should be in place

Developing CDMAs and the CRFs within them must be done using defined procedures, with tasks and responsibilities clearly delineated for design, development, testing and deployment. Controlled documents should therefore exist covering these areas. The evidence that the standard has been met would be the relevant documents.

DM01.02 Cross-disciplinary CDMA development

CDMA and CRF development is performed by a cross-disciplinary team (e.g. investigator, trial manager, statistician, data manager, programmer)

Developing CDMAs and the CRFs within them should involve the various users of the system, or key representatives of those users.

These would normally include an investigator and / or sponsor representative, plus the trial manager, statistician, and members of the data management and IT staff. The input from each would vary between different tasks. As a *minimum*, the expectation is that a representative of those setting the CDMA up (i.e. the IT staff), those collecting the data (i.e. the trial's data management staff) and those analysing the data (i.e. the trial statistician) are all involved in CDMA design and development.

Evidence that the standard has been met would come from

a) inspection of the relevant controlled documents;

b) discussion with staff to clarify how the CDMAs were developed;

c) the range of names and signatures involved in signing off documents relevant to CDMA / CRF production.

DM01.03 Requirement specifications of CRF

The specification for CRFs is driven by the protocol (e.g. primary safety and efficacy variables)

The final decisions about the CDMA design and contents may rest with the sponsor, but an ECRIN data centre has to be far more than just the passive recipient of somebody else's specification - it needs to input its expertise as an active partner in developing CDMAs.

A fundamental requirement is that the centre works with the sponsor to ensure a clear link between the protocol and the set of CRFs, with the CRFs capturing relevant and sufficient data but avoiding redundant questions and those that 'might possibly be useful one day'. One way of doing this is to first use the protocol to specify the data that the statisticians will need to carry out the required analyses, and then use these *analysis data requirements* in addition to the relevant safety parameters to drive the CRF specification.

It will be difficult for ECRIN auditors to assess this standard in detail in the context of specific trials, but it should be possible for the centre to describe and demonstrate how the general process of CRF construction and review is based upon the requirements of the protocols (accepting that in a particular case the sponsor or investigator may have the final say about CRF design).

DM01.04 Design of CRFs

CRF development is compliant with procedures described in controlled documents and includes version management

CRFs change over time and the overall development and deployment process should therefore include CRF versioning, as well as being clearly compliant with the procedures described in the relevant controlled documents (see DM03 for standards dealing with CDMA change management).

Version management should include clear records of when new versions were signed off and introduced into the system (possibly on a site by site basis), as well as clearly indicating the differences between versions and the reasons for the changes.

Evidence that the standard has been met would come from inspection of the CRFs and relevant specification documents.

DM01.05 Functional specifications of CRFs

CRF design and functional specifications exist identifying each data item on each CRF (including field names, types, units, validation logic, conditional skipping)

A key aspect of developing an eCRF is creating a full functional specification, characterising all the data items and associated validation (i.e. data checking), skipping and derivation logic.

The specification may contain an 'annotated CRF' (though on its own this is unlikely to contain all of the required information and will usually need to be supplemented by other documents) or it may exist as an entirely separate set of documents, for instance as a set of spreadsheets or as a database report.

CDMA programmers can use the specifications to accurately build the eCRFs. This is not necessarily a single 'specify then create' process - often an iterative approach will be used - but the CRF should still be clearly based upon a specification. Without such a specification it becomes very difficult to properly validate, and document the validation, of the final eCRF.

Evidence that the standard has been met would come from
a) inspection of CRF specifications;

b) discussion with staff to clarify how the specifications are developed;

c) relevant controlled documents.

DM01.06 Specification approval

CRF design and functional specifications are signed off and dated by relevant signatories

Once CRF / CDMA specifications have been constructed they will need to be formally approved and signed off by the key individuals involved with the trial.

The final specification may not be signed off until at the end of the CRF construction process (i.e. it does not always have to be fully approved before the CRF is begun) but the CRF should still be clearly approved against a specification.

The evidence that the standard had been met would come from relevant approval signatures on CRF specifications.

DM01.07 Isolation of development CDMAs

CDMAs in development should be isolated from CDMAs used productively

A CDMA should be developed within an environment reserved for development and test activity only. The development and production systems should be isolated from each other - there should be no possibility of any problems in a developing CDMA spilling over to affect any production system, or of users inadvertently confusing the development and production instances.

This could be done by having distinct data stores (e.g. different databases or even database servers) for the development and production environments, or possibly two distinct instances of the CDMS.

The evidence that the standard had been met would come from:

a) explanation and demonstration by centre staff of how the CDMAs in development were kept isolated from production systems;

b) inspection of relevant controlled documents.

DM01.08 Isolation of training eCRFs

Access to the CDMA for training purposes is managed to ensure that is isolated from clinical data

Users need to be trained on CDMAs, generally using dummy or test data, and it is important that this data is kept separate from actual study data.

User access for training purposes must therefore be managed to ensure that this is the case, sometimes by using a completely different CDMS instance and / or data store for training than for production, sometimes by setting up dummy 'training sites' within the production system (the data from which is excluded from analysis).

The evidence that the standard had been met would come from:

a) explanation and demonstration by centre staff of how the data generated in training was kept separate from actual study data;

b) inspection of relevant controlled documents.

DM01.09 Production of interim CRF

For trials / sites using eCRFs, procedures should be in place to generate accurate iCRFs (interim CRFs) for sites, if and when necessary

A centre should be able to generate so called interim CRFs or iCRFs, if required and if the sponsor agrees this would be appropriate.

These may be needed in eRDC systems if direct data entry into the system is not possible or desired during initial data collection. Anecdotal evidence suggests that this is a common situation, especially as many site staff find it difficult, and rather unsympathetic, to interview subjects and use an eRDC system at the same time.

In such circumstances the research staff at the site are far safer using structured paper documents that match the eCRF to note down responses and other data, rather than blank sheets of paper or whatever else might be available. The system should therefore be able to produce such iCRFs, ideally directly at the site ('system' being all available systems and processes, including but not limited to the CDMS).

In some cases the iCRFs can be as simple as screen shots of the eCRF screens, though they should include a mechanism for noting the subject's name, number or similar unique identifier. The important thing is that they allow data collection

to be structured in the same way as if the eCRF was directly available, and safely stored before it is transferred to the eRDC system.

The evidence that the standard had been met would come from:

a) explanation and demonstration by centre staff of how interim CRFs could be created;

b) inspection of relevant controlled documents, detailing the procedures to be followed.

Further Indicators of Good Practice in CDMA Design and Development

Listed below are several examples of 'best practice' in CDMA development and CRF. They do not form part of the ECRIN requirement but their usage provides greater confidence that procedures for CRF creation are well developed and applied consistently.

- *Using libraries and metadata repositories:* Having libraries available of items and forms, or a more formal metadata repository, enables reuse of data items and a consistent approach to coding and naming, especially if backed up by local guidance documents. Such libraries can also promote the consistent use of repeating question groups (or alternatively lists of single questions) within particular domains.
- *Consistent local coding systems:* Common principles applied to item design and metadata (e.g. preferred coding systems, even for 'yes' and 'no', styling and numbering of items, the coding of different types of missing data, preference for positive formulated questions, etc.) can all make systems more consistent and easier to use.
- *Using standard coding systems (e.g. CDISC CDASH):* In some domains international standards are available for data item codes and definitions, especially those defined by CDISC within the Clinical Data Acquisition Standards Harmonisation (or CDASH) project.
- *Using standardized questionnaires and instruments:* Using validated questions, scales or standard instruments (e.g. for quality of life questionnaires) improves the reliability of the final results and, if already available in a library, speed development. Decisions about the use of such validated instruments are ultimately the sponsor's, but a data centre should have them available and be able to promote their use.
- *Local design and guidance documents:* Local documents specifying good design practice and preferred orientation, colours, fonts, graphics, positioning etc. (so far as the CDMS allows variation in these) can promote consistency and a 'house style'. Consistent and sensible use of dividers and sectioning, and white space, can also add to consistency and the ease of use of systems.

DM02 CDMAs - Validation

Once a CDMA has been constructed it must be validated to ensure that it works as intended and is fit for purpose. The validation required will follow the general principles described in IT06, but there are CDMA specific aspects of validation that are commonly applied, and which are collected together in this section.

Validation in this context does not refer to *data* validation, i.e. the process of checking that data contains reasonable values and is logically consistent. That process is better referred to as (logical) data checking. It does apply, however, to the process of verifying that the data checks operate correctly, and indeed this 'checking the checks' usually forms a significant part of CDMA validation.

Because CDMAs are specified and built in-house, they can normally be amended relatively easily until they meet their original specification, though occasionally the validation process itself may trigger last minute changes to the specification. The validation process would normally be shared by IT and data management staff, as well as end users (see DM02.04), to ensure that the system and its constituent eCRFs were fit for purpose.

DM02.01 CDMA validation policies

Controlled documents for CDMA validation are in place

There should be a general procedure for CDMA validation, specified in controlled documents, detailing procedures, responsibilities, outcomes etc., even though each individual CDMA will need its own specific validation documentation.

Evidence that the standard had been met would come from the controlled documents themselves.

DM02.02 CDMA Specific test plan

A trial-specific test plan and a test documentation set exists for each CDMA.

Each CDMA will require its own set of specific validation documentation. These will usually be based on the general procedures but list the specific study parameters (e.g. uniqueness checks), and eCRF logic checks, skips, and derivations in test documents. Such documents can then be completed with the result of the tests recorded for each individual element.

Some CDMAs may require additional testing, for instance to check access from particular sites, or particular functionality (like coding, or message triggering) that is not found in other study applications. The testing of these should form part of the valuation plan. In other words validation should, as usual, be based on a risk assessment and identification of the elements that need to be tested.

Evidence that this standard had been met would come from examination of the CDMA specific validation documentation for a range of studies.

One approach to CDMA validation involves completing test pCRFs (or iCRFs), inputting them into the CDMA, and then exporting them again in a form that is readily comparable with the original data.

This has the advantage of testing overall usability as well as many of the functional components of the system, and more importantly it also means that the extraction / reporting functions are tested as well - something that may be more important if locally built routines are used for part or all of the extraction.

The main disadvantage of this approach is that - unless enormous care is taken in preparing a large set of test data - not all functional components of the system will be systematically tested. If used, the method should therefore probably be seen as an addition to the detailed testing of each component above.

Evidence that the standard had been met would come from examples of trial specific test documentation, along with discussion with staff to clarify how it was used in practice.

DM02.03 CDMA testing against functional specifications

Testing with sample data against functional specifications is carried out for each CDMA before deployment to live environment

One of the key aspects of CDMA validation is the detailed testing of each CRF against its functional specification.

This will include checking the correct data items are there, of the right type, with the specified codes and code lists, and in the right order. Most of the testing effort, however, will be centred on the logic built into each CRF - the range and consistency checks, the skipping (or enabling / disabling) logic, and the generation of any derived values. Each of these checks should be separately documented, with - for the logic checks - input values and the system's response.

Ideally, the system should be able to generate some of the necessary test documentation itself, for instance it should be able to generate a listing of all the logic checks on a particular CRF. These might then need further processing to create a proforma (or a database) for recording the test results.

Alternatively, some data centres use their system for generating and recording the CRF functional specifications to also produce the test documentation, and to record the results of those tests - something that is relatively easy to do with specifications stored in databases rather than spreadsheets.

The evidence that standard has been met will come from the detailed test records for a range of CDMAs.

DM02.04 Assessment of CRFs by users

Users are involved in assessing CRFs for ease of use

An important part of CDMA testing is assessing usability. Rather than checking individual components, this considers the CRFs as a whole and how easy they are to navigate, identify and select items, and work with in terms of raising or responding to queries. To be valid, such checking needs to involve a sample of actual users (ideally with different levels of experience).

Note that the requirement is not for formal *usability testing*, which is a more sophisticated process involving detailed measurement of responses and response times, and which would be beyond the resources of a data centre. The standard asks instead that some users are involved in the testing process and can provide feedback.

Evidence that the standard had been met would come from explanation by centre staff of how user feedback is gathered, plus inspection of documents including user feedback and / or sign off, against a range of specific CDMAs.

DM02.05 CDMA approval

Each CDMA should be formally approved, dated and signed by the relevant signatories, before production use.

Once CDMA validation has been completed it needs to be signed off, normally by a small cross-disciplinary team but as a minimum by the trial or project manager who will oversee the use of the CDMA in supporting the study. In most cases a single sign off will cover the whole CDMA, but some centres may have each CRF signed off separately.

Evidence that the standard has been met will be appropriate dated signatures confirming that the CDMA is OK to be used as a production system.

DM02.06 Validation detailed findings

All validation results, including any test data and protocols, are retained for each CDMA

All the detailed test documentation / systems, as well as the results, and any scripts, dummy data, listings etc., used for any particular validation should be retained. Much of this may be in electronic form rather than on paper.

The evidence that the standard had been met would be:

a) an explanation and demonstration by centre staff of how and where the detailed test results were retained;

b) inspection of actual results against a range of CDMAs.

DM03 CDMAs - Change management

Even after a CDMA has been successfully validated and moved into production changes will be requested. Such changes must be carefully managed to ensure that the system retains its validation status.

The change management required follows the general principles outlined in IT06 (standards IT06.06, .07 and .08 in particular) but CDMA change is relatively common, and its proper management critical to data management, so a separate section of standards is justified.

DM03.01 Change management of CDMA

Controlled documents for CDMA change management are in place

Controlled documents should be in place dealing with CDMA change management, detailing procedures, roles and responsibilities and documentation.

Evidence that the standard has been met will be the controlled documents themselves.

DM03.02 Documenting change requests

Individual requests for change to CDMAs are justified, itemised and documented

The initial step in the change management process is to ensure that any requests for change to the CDMA are properly described and authorised. This would normally involve a paper or screen based proforma being completed with the necessary specification of and justification for the request.

Evidence that the statement had been met would be from inspection of such proformas.

DM03.03 Change and risk analysis

A risk analysis is conducted and recorded when considering any change

The change management process must include an assessment of the *potential impacts and risks* associated with a proposed change. For relatively trivial changes (addition of additional categories to a code list for instance) these impacts may be small; for large changes - e.g. the addition of a new eCRF - they may be considerable.

Changes that would risk orphaning data already in the system (e.g. dropping questions or categories) or making existing data invalid (e.g. changing the type of a question) should not normally be allowed and the change request should be rejected.

Any change will impact the CDMA itself, but there may also be impacts 'downstream', for instance on the data extraction process or the scripts used during statistical analysis, or on system documentation and / or user training. A CDMA change may also imply a change to the protocol (see DM03.06).

It is important that all these aspects are taken into account. Some centres use a 'change checklist' approach to structure the assessment of risk and to help with its documentation.

Evidence that the standard had been met would be the inspection of the risk assessment documentation against a range of proposed CDMA changes.

DM03.04 Testing of CDMA changes

Any change is tested in the development / test environment and the test results are recorded

The risk analysis (see DM03.03) will determine the amount and type of re-validation required. This should always take place in the development / test environment and the results recorded.

In a busy data centre it is sometimes tempting to make and inspect trivial changes in the production environment, but then the flow of versions between the two environments is disturbed, and the next import of a study definition from the test environment will overwrite the earlier change.

All changes should therefore be implemented in the development environment first, and the revised system then exported to the production environment. This also makes it easier to store each version of the study definition metadata file for future reference.

Evidence that the standard had been met would come from inspection of the detailed test results relating to changes.

DM03.05 Communicating changes

Mechanisms are in place to inform relevant staff and users of changes, and provide support and explanatory material as required

The potential impact of any change on users should also be considered. In most cases data entry staff will need to be informed of changes and why they have been introduced, and so mechanisms should be in place to allow this to happen consistently.

For substantial changes there may also be a need to provide additional training, and the communication should reflect that.

Evidence that the standard had been met would come from explanation by centre staff of how the system worked, the relevant parts of controlled documents and from examples of the mechanism in action.

DM03.06 Changes and protocol revision

Processes should exist to ensure ongoing consistency between a CDMA and the associated trial protocol

There is a fundamental requirement for the CDMA to provide the data collection requirements implicit in the study protocol.

When the protocol itself changes the process of cascading those changes to the CDMA is fairly straightforward, because it is the direction in which change would be expected to flow.

From time to time, however, a requested CDMA change may be significant enough that it represents a change to the protocol, even though it may not have been initially recognised or presented as such.

A change to the protocol will itself trigger a requirement for review by ethics and regulatory bodies, and the proposed change cannot be introduced into the production system until the relevant re-approvals have been obtained.

Thus, whether or not a requested change represents a protocol amendment should be part of the evaluation of any change (for instance part of a 'change checklist'). If it does, then procedures should exist for the necessary actions to take place so that the protocol amendment is managed properly and effectively, and integrated into the change management process.

Evidence that this standard had been met would come largely from inspection of the relevant controlled documents and associated proforma, together with discussion of any examples of the mechanisms being used in practice.

DM04 Data Entry and Processing

The standards in this section deal with data entry into the CDMA. Most modern CDMSs make this very straightforward but, as one of the core processes of data management, it still requires a framework of policies and procedures if it is to be carried out consistently to agreed standards.

DM04.01 Data entry policies

Controlled documents for data entry and corrections are in place

Some of these documents may be generic (e.g. general policies on using self-evident corrections) but others may be trial specific and usually found within the Data Management Plan for the trial (e.g. the specific self-evident corrections that have been agreed as acceptable)

Evidence that the standard had been met would be the controlled documents themselves.

DM04.02 Access control for data entry

Access control is fully implemented; data entry / review is only accessible to authorised personnel and according to need

Data entry must take place in the context of controlled access, i.e. adhering to the centre's own policies on access control.

This is a special case of the access control already required under IT 04.02. It is included here partly to provide an additional emphasis on access control within the CDMS, partly because access control for data entry is often a joint responsibility of IT and data management staff, and partly because it is often the subject of specific policies and controlled documents.

The evidence that the standard had been met would come from

a) the controlled documents dealing with CDMS access control

b) demonstration of the access control system

DM04.03 Restriction of site data access

Site staff only have access to the data of their site(s)

An important aspect of being able to access data only 'according to need' is that remote site staff only have access to the data (and related material like queries) of their site.

This is a special case of the granularity of access control already required under IT 04.03. It is included here to provide an additional emphasis on access control

within the CDMS, and partly because access control for data entry is often a joint responsibility of IT and data management staff (including sometimes staff at the remote site) and therefore may be subject to slightly different procedures.

The evidence that the standard had been met would come from

a) the controlled documents dealing with CDMS access for site staff

b) demonstration of the access control system

DM04.04 Management of missing CRFs / Data

Mechanisms are in place to identify and report on missing or late pCRF / eCRF and safety data

Monitoring what data has arrived is part of the data entry process, so that sites can be contacted to request missing or late data. Most eRDC systems make this straightforward, with the system set up to identify missing data and the centre able to send messages to sites to query that data. With trials using paper CRFs there is usually a need for a separate pCRF tracking system (see DM04.05).

Evidence that the standard had been met would come from:

a) the relevant controlled documents;

b) demonstration of the missing / late data management system(s) and explanation of their use in practice.

DM04.05 Tracking of pCRFs

For pCRFs a receipt tracking system is in place.

With trials using paper CRFs there is often a lag (from several days to several weeks) between CRF receipt and the addition of the data to the CDMS, so that the CDMS cannot be used reliably to monitor receipt of data. It is therefore necessary to have a separate CRF tracking system in place, unless the lag time can be guaranteed to be limited to a few days.

A useful feature of CRF tracking systems (and scheduling systems within eRDC system) is the ability to automatically truncate a subject's schedule when notification is received that the subject has died or is lost to follow up, or at least allow easy manual amendment. This avoids irritating sites by requesting data that will never exist. This is not currently part of the standard but is regarded as best practice.

The evidence that the standard had been met would come from

a) the relevant controlled documents;

b) demonstration of the pCRF tracking system and its outputs.

DM04.06 Patient blinding requirements

Processes exist to allow the blinding of inappropriate patient identifying information submitted to the centre

One of the errors that can be occur with pCRFs and safety data is patient identifying information being incorrectly added or retained on submitted data.

In some cases this may contravene national regulations, in others the policy of the centre and / or sponsor. In either case the identifiers should be removed or blocked out and the site reminded of the requirement to omit such identifiers. In an eRDC system this problem should not arise, assuming it has been designed to conform to blinding requirements from the start.

The evidence that the standard had been met would come from

a) relevant controlled documents;

b) discussion with staff and demonstration of the blinding being put into action.

DM04.07 Simple checks used

Simple checks on single values (e.g. range checks) should be available and used where appropriate

The data entry process should result in the firing of simple checks (i.e. with single value inputs, like range checks) as and when appropriate, and the subsequent generation of queries.

Most systems support two types of checks:

a) 'Hard' or 'Reject' checks that will refuse to accept any data that triggers them, where the user must leave the data item blank or put in a more acceptable value, and

b) 'Soft' or 'Warning' checks that will trigger a warning message, to prompt the user to change the data item before saving it, but will allow the original data to be input if that is what is actually in the source data. Usually the system will then label the data item, with a suitable icon, as somehow odd or unexpected.

With eRDC systems hard checks can be useful so long as the trigger response represents something that genuinely could not be correct - for instance a date given in the future for something that must have happened in the past.

For systems with the data submitted as pCRFs hard checks should be used with great care, if at all, because if the user is to accurately input the pCRF's data (however strange or seemingly impossible) they will need to be able to input any values. Hard checks may therefore result in data items being left blank when data exists, and ultimately an incorrect audit trail.

The evidence that the standard has been met would be:

a) Demonstration of simple checks on a variety of eCRFs

b) discussion with centre staff justifying their appropriate use.

DM04.08 Complex checks used

Complex checks on multiple variables (e.g. for logical consistency across forms) should be available and used where appropriate

The data entry process should result in the firing of complex checks (i.e. with multiple input values, such as cross form consistency checking) as and when appropriate, and the subsequent generation of queries.

Exactly how complex such checks might be is partly dependent upon the capabilities of the CDMS. Few CDMS systems for setting up logic checks, however, can match the functionality available in statistical packages or database languages, and the more complex the check the more difficult and time consuming it can be to validate it. Although the advantage of immediate feedback will be lost, some very complex checks might therefore be better done by checking directly against the data after data entry, or within an extracted data set.

The evidence that the standard has been met would be:

a) Demonstration of complex checks on a variety of eCRFs

b) discussion with centre staff justifying their appropriate use.

DM04.09 Self-evident corrections

Clear guidelines and procedures should exist to identify and carry out self-evident corrections

In some cases the data on pCRFs is obviously incorrect and would fire a warning or reject message if input, but it is clear what the correct data should be - the error has been caused by a common omission, addition or transposition. An example would be '07/11/209' or '07/11/20009' for '07/11/2009', or the omission of a response to the 'Any Adverse Events?' question followed by a report of three adverse events.

In such cases it does not make sense to query the site, and a self-evident correction (or an 'automatic obvious data modification') can be used to amend the data. The use of such self-evident corrections must be tightly controlled however, restricted to a pre-agreed list of situations where they could be applied, normally agreed at the level of the individual study. In addition there should be a clear procedure to follow when self-evident corrections are applied, including instructions on how the source document should be marked to indicate that the correction had been made.

The evidence that the standard had been met would include:

a) the relevant controlled documents (e.g. examples of data management plans with self-evident correction instructions in them);

b) discussion with and demonstration by the centre staff of the procedure in action.

DM04.10 Audit trail

All transactions in the CDMA (insert, update, delete) must have an audit trail, covering the date and time of the input, the person making the change and the old and new values

Providing an audit trail of the CDMS transactions is a regulatory requirement. For instance the FDA (CFR 21 (11), section 11.10(e), 2010) requires the

"Use of secure, computer-generated, time-stamped audit trails to independently record the date and time of operator entries and actions that create, modify, or delete electronic records. Record changes shall not obscure previously recorded information."

Modern CDMSs normally support such an audit trail.

The audit trail requirements do not include a 'reason for change' (RFC) as a mandatory data item, though many CDMSs support this as well. Some data centres like to make use of this feature, others are less convinced of the utility and accuracy of the data recorded.

Evidence that the statement had been met would come from demonstration of the audit trial being created in a test database.

DM04.11 Timestamp control

Sites using eRDC should not be able to change the CDMS's time stamp

Because an accurate time stamp is an integral part of the audit trail, it is important that there is no ambiguity about the time recorded against data activity. In particular it should only be possible to set this time centrally, i.e. at the data centre, and not at the remote sites.

Most CDMSs support this feature automatically, and also record both the local time at the data centre and the time at the remote site inputting data, usually as the data centre time +/- *n* hours according to the site's time zone.

Evidence that the standard had been met would normally come from the CDMS documentation and demonstration of the use of local / site times within the data.

DM05 Data Quality Checks

A data centre should be able to run checks on the accuracy and consistency of the data it contains, for example checking the database contents against paper CRFs, or analysing the data for medical consistency.

The standards in this section cover this area, but they are concerned only with the data quality checks that occur in the centre - they exclude those that take place at sites, and specifically *they exclude source document verification* (SDV) even though SDV is an important mechanism for checking data quality.

DM05.05 is concerned with how a centre can *support* SDV but there are no standards dealing with how SDV should actually be carried out, in either a pCRF or eRDC context. This is because SDV is normally carried out as part of a monitoring rather than data management function (though obviously these areas overlap) and was therefore originally seen as out of scope of these standards.

(In the future standards relating to SDV may be included as part of a set of standards for monitoring, allowing data centres who wish to be known as having good quality monitoring services to become certified against those standards).

Note that there is also no requirement in these standards for a data centre to be able to carry out double data entry (DDE). Not all CDMSs support DDE, and even those that claim they include it implement DDE support inconsistently. Many also question the value of the technique, in terms of the time and effort required against the accuracy gained, especially when so many data entry errors can be caught by the built in data checking logic of modern CDMSs.

DM05.01 Data quality policies

Controlled documents are in place regarding data quality and the checking required to support it

There should be general or default policies and procedures covering this area, for instance including the error levels that would trigger a further, more extensive examination of the data. In any particular case, however, the details of the data checking regime might be modified by the sponsor and / or trial management team (and be described in the study specific data management plan).

The evidence that the standard had been met would be the controlled documents themselves.

DM05.02 Batch validation checks

Validation checks can be executed via a batch process, to identify missing, illogical and inconsistent data and are used where appropriate

One method to carry out checking is by 'batch validation' - essentially the application of the sort of check logic that is applied at data entry to the data of one or more CRFs of a group of subjects.

In many cases batch validation is a feature of and takes place within the CDMS, and the logic applied will then usually be the same as that applied during normal data entry. Batch validation in this context is highly desirable when a new check is applied after data has been entered, and the existing data must be validated against it, or data is imported in bulk (e.g. from a laboratory system) and checks were not available during the import process, or if the normal checking on data entry has been turned off (as allowed by some CDMSs), for instance to speed up data entry.

Batch validation can also be done independently, however, for example by running SQL scripts against the database or by exporting the data to a statistics package and analysing it there. In these cases the actual logic used for checking might be the same as or an addition to the logic used within the CDMS during data entry. This method is particularly useful if checks are required that are difficult to formulate within the CDMS.

Many centres run both types of batch validation, according to need, mimicking the checks of data entry in some circumstances, augmenting them in others. Note, however, that the requirement is that centre can run one or other types of batch validation if required, not that it is routinely doing so.

The evidence that the standard had been met would come from

a) demonstration of either or both methods of batch validation;

b) controlled documents detailing the use of batch validation;

c) discussion and explanation by centre staff of how and when such methods are (or would be) actually used.

DM05.03 Data review

Validated reports are available in formats to support the manual review of data (e.g. for consistency checking, medical review)

Manually checking data against source documents (pCRFs, lab data sheets etc.), or against data from elsewhere in the database (e.g. to check the medical consistency of reported symptoms, lab. values etc.) is made much easier if reports can be generated that present the data in a suitable format - e.g. the same, or approximately the same, way as the source documents, or in line listings that bring together the data for easy comparison. Such reports may be built in to the

CDMS or need to be constructed separately (either way they will need to be validated).

The evidence that the standard had been net would be demonstration of the relevant reports, supported by a discussion with staff as to how they are used.

DM05.04 Supporting source data verification

The centre has procedures for supporting source data verification, as a minimum providing access to its data for those implementing and conducting the SDV

The sponsor will normally determine both the SDV strategy required and decide who will be doing the SDV. Pharma sponsors may, for instance, want to use their own monitors for SDV. Even non-commercial sponsors may wish to use a different trials unit for the monitoring / SDV function than for the data centre function.

What a data centre does need to do is *support* the work of monitors carrying out SDV, by making the trial data available to them. There should therefore be procedures in place for allowing monitors access to the data so that they can inspect and assess it, and for exporting and presenting data on demand, on a subject by subject basis, to monitors.

It would be good practice, though not currently a formal requirement, to further support SDV with reports detailing query rates and late data (or any other indicators of problems during data entry) on a site by site basis.

The evidence that the standard had been met would be the controlled documents describing the relevant procedures, together with explanations from staff about how they worked in practice.

DM05.05 Documentation of checks

All data checking exercises should be documented and analysed, and any emerging issues reported to the appropriate person(s) for resolution

Data review exercises are of limited value if they are not recorded properly, with details and summaries of results plus the dates and personnel involved. They are also of limited value if they do not lead to action to resolve identified problems.

Thus there should be clear 'resolution pathways', supported by appropriate procedures where necessary, for instance to generate queries, increase the checking activity, retrain data entry staff, provide additional input to a centre etc.

The evidence that this standard was met would come from:

a) relevant controlled documents outlining the procedures to be taken in recording data reviews and in dealing with identified issues;

b) examples of actual data reviews and consequent actions.

DM06 Query Management

Query management is usually integrated into modern CDMSs, with queries raised, annotated, responses reviewed and the queries closed all on screen, the CDMS acting as the transport medium between centre and sites.

For paper based trials queries must be raised and tracked separately, in some centres using IT systems developed for the purpose, in others more basic tools like spreadsheets.

The standards in this section apply to both types of query management.

DM06.01 Query Policies

Controlled documents are available covering query format, generation, data change and resolution

Whatever the detailed mechanisms the production and resolution of data queries should be regulated by appropriate policies and procedures, detailing actions, roles, responsibilities and documentation.

The evidence would be the controlled documents themselves.

DM06.02 Query creation - data entry

Queries can be created - automatically and / or manually - based on documented staff roles, procedures and pre-constructed logic checks

Queries are commonly created during data entry, as a function of the omissions and discrepancies noted by data entry staff, usually prompted by the validation messages generated by the check logic in the CDMA.

Only certain staff should be able to generate them, usually with particular roles within the CDMS, and they should be generated and distributed according to the relevant procedures within the centre.

This standard simply requires that this is confirmed, and the evidence it had been met would come from an examination of queries generated and a discussion with staff about how the relevant controlled documents are applied in practice.

DM06.03 Query Creation - Batch Process

Queries can be created in accordance with documented procedures from batch checking of data, as necessary

Queries can also be created from batch validation (see DM05.02) and especially during analysis of extracted data sets, when missing, inconsistent or odd values can be identified by applying tests and scripts. Queries can then be generated for sending to sites, a process which may or may not involve the CDMS.

The standard requires that these queries are created according to the relevant procedures, so confirmation of compliance would be from:

a) the relevant controlled documents;

b) examination of generated queries;

c) discussion with staff as how such queries are (or could be) created, distributed and used in practice.

DM06.04 Tracking of queries

Responses are recorded when returned, identified when outstanding and queries resent if necessary

Having sent the queries out, through an eRDC system or by post or courier, the centre needs to be able to track the responses to them and identify those for which no response has been received, or for which the response is unclear, resending the query or generating a new one if necessary.

If queries are sent out through the eRDC system that system will normally have such tracking functionality built in. For trials using pCRFs a separate query tracking mechanism will be necessary For best practice it would be linked to the query generation process and include functionality to prevent duplicate queries being sent out to sites, though this is not a formal requirement.

Evidence that the standard has been met would be demonstration of the query tracking system(s) that showed how queries were recorded and tracked.

DM06.05 Actions in response to queries

Query resolution is tracked, and appropriate actions taken and documented.

Once a query responses has been received a decision is made as to whether it is fully answered or not, and a supplementary query sent if necessary. If the issue has been resolved values in the CDMA may need to be changed.

For most eRDC systems with integrated query management the link between the query, its response and the value in the database, whether or not it has been changed, will be obvious and visible on screen. For pCRF based trials with separate query management, many centres use a comment or 'reason for change' field to link the data value to the query or queries associated with it (for instance storing a query ID number).

Either way the record of the query and its resolution should be linked to the data item, either in the CDMS or in a separate query management system, effectively making the query part of the audit trail.

The standard would be met if this is shown to be the case.

DM07 Delivery and Coding of Data for Analysis

This standards in this section deal with the ways in which trial data is prepared, checked, fixed in some way, and then extracted in the format required for analysis.

The specific processes used for generating analysis datasets will vary, depending on the longevity and type of trial as well as the purpose of the analysis. For example, for a self-contained study where there will be no further data collection, the database is often locked down (or 'frozen', though the exact definition of 'locked' and 'frozen' varies between systems) so that no further data entry or amendment is possible. For a longer term study where data collection may continue for many years after the primary analysis, or where various interim analyses are necessary, it would be more usual to export a 'snapshot' of the data state.

Note that there is no requirement relating to the format of the extracted data. That will normally be as agreed with the statisticians that carry out the analyses - examples include CSV, XML, and SAS, R and SPSS native formats.

DM07.01 Policies for database locking

Controlled documents should be in place dealing with taking a snapshot of the trial data, and / or 'locking' and 'unlocking' that data

All process(es) by which data is prepared and extracted for analysis should be governed by clear procedures, documented within controlled documents.

The relevant evidence would be the controlled documents themselves.

DM07.02 Data completion

All relevant data (or all except for a pre-defined / pre-agreed fraction) should be received prior to data extraction for analysis

Extracted data need to be as complete as possible. In some cases database lock is dependent upon completion of data entry, in others a snapshot is taken once all data expected by a certain point is in, or at least - e.g. for an interim analysis - all that can be reasonably expected in a given trial at a given time.

The evidence that this standard was being met would be:

a) the relevant controlled documents;

b) examples of communication and / or a checklist relating to database lock / snapshot and the levels of data required.

DM07.03 Query resolution completion

All queries (or all except for a pre-defined / pre-agreed fraction) have been resolved prior to data extraction for analysis

Queries will also need to be resolved before database lock or snapshot. In some cases this will mean all queries, while in others some exceptions may be allowed. The rules governing any exceptions should be explicitly defined and agreed.

Data consistency checks will also often generate additional queries during the final phase of preparation for analysis, leading to an upsurge in query generation with, very often, faster timelines for their resolution (see DM07.05).

The evidence that this standard was being met would be:

a) the relevant controlled documents;

b) examples of communication and / or a checklist relating to database lock / snapshot and the query resolution required.

DM07.04 Data reconciliation

All external data (e.g. safety database, lab data) has been reconciled prior to data extraction for analysis (or all except for a pre-defined / pre-agreed fraction)

Data preparation may also involve reconciliation of the data input through the CDMA with that received from elsewhere - for example between expedited SAE reports and the more routine adverse event reporting , or between sample and laboratory result data. This should be brought up to date before the database is locked or a snapshot is taken. If exceptions to data reconciliation are allowed, they should be defined, agreed and documented.

Where data coding has been used (see DM07.08, DM07.09) it would be normal for that coding to be reviewed as part of the data preparation. In some instances a data quality check may also be done, especially if one has not yet been performed on this data. Whatever the detailed arrangements specified by the relevant controlled documents, a check list dealing with the different aspects of data preparation can be a convenient way of ensuring all the aspects are covered and recorded.

The evidence that this standard was being met would be:

a) the relevant controlled documents;

b) examples of communication and / or a checklist relating to database lock / snapshot and the need for data reconciliation.

DM07.05 Post lock data amendment

Controlled documents should be in place detailing procedures to be followed if data needs to be altered after the snapshot or DB lock

Despite the best planning and preparations, there may be occasions when amendments are required to the data after the database has been locked, or to snapshots after the extraction has actually taken place - perhaps to correct errors that come to light at the last moment, or to incorporate late returned query data. In such cases it is essential that the unlocking / amendment process is tightly controlled and documented in any given instance, as demanded by this standard.

The evidence that this standard was being met would be:

a) the relevant controlled documents;

b) documented examples of post lock data amendment.

DM07.06 Read only retention of analysis data

The data provided for analysis is retained within a read only regime, and is available as a reference data set for any future re-analysis or audit

There will be a need to arrange the long term retention of any extracted data, partly for audit or inspection purposes and partly to allow - if necessary - the reconstruction of any analysis using the same extracted data. This would normally be done by placing the relevant files within an area of the centre's storage capacity that is read only (except for the IT staff that do the transfer).

The evidence that this standard was being met would be:

a) the relevant controlled documents;

b) demonstration of read only retention for a range of extracted data sets.

DM07.07 Extracted data validation

The data generated for analysis should be validated against the data in the clinical database, or the extraction process itself is validated

The processes used to extract and, if necessary, transform data for analysis will need validating (see IT08.03 and IT08.04). If the extraction method is part of the normal functionality of the CDMS the validation will probably already have been done, as part of the OQ / PQ of that system. If it involves additional, locally constructed processing of some kind then that processing will need validation, and/or the data in the extracted set will need to be compared with the original data in the CDMA to check that they match.

Evidence that this standard was met would come from the detailed records and summary statement(s) relating to the validation of the extraction process(es).

DM07.08 Policies for coding

If data coding is carried out, controlled documents are in place detailing the procedures to be used

In many data centres some data is coded using international standard systems, usually as an aid to reconciliation, classification and analysis of data. The best known example is MedDRA for adverse events (and in some case medical history) coding, but other coding systems include the WHO ICD system for mortality and morbidity data and the WHO Drug Dictionary sometimes used for concomitant medications.

Using such systems involves more than the simple application of codes to matching terms. Code allocation may be ambiguous, and the standards exist in different versions, so policies and procedures must be developed to support consistency in coding and to stipulate the versions to be used, or at least how decisions about version should be reached.

Autocoding mechanisms generate much discussion. While they may make the coding process quicker many staff feel they can too easily blur the distinctions that often have to be made between coding in one trial and in another. For that reason some staff prefer to use autocoding only within one trial at a time, and others are suspicious of them in general. Clear policies should therefore also exist to govern the use of autocoding mechanisms, if any are used.

The relevant evidence would be the controlled documents themselves.

DM07.09 Coding training

If data coding is carried out, it is carried out only by personnel trained on the relevant systems with access to authorised trial specific support material

Because applying codes is not straightforward the staff that do it need to be properly trained to carry out that task. In addition it is often necessary to supply such staff with support material - e.g.in MedDRA coding, a list of commonly linked symptoms that should be coded as a single entity, and a list of such symptom pairs that should be coded separately.

Common adverse events which can be classified in different ways (i.e. in MedDRA terms allocated to different system organ classes) may need to be listed against the classification that should be used - usually on a trial by trial basis. The responsibility for authorising such support material would normally fall to the sponsor / investigator, but the centre needs to ensure that such material is prepared and that the staff know how to use it.

Evidence that this standard had been met would be

a) relevant training records for the staff involved;

b) examples of authorised trial specific material to support coding.

GE01 Centre Staff training and support

The standards in this section are concerned with the initial and ongoing training and support for the data management and IT staff that directly support the data centre. In most cases such staff will be based in the centre, though some IT staff may be based in IT host organisations. The standards do *not* apply to site based staff - training and support for these is dealt with in Section GE02.

During an audit the focus will be on the IT / DM staff and the documentation (e.g. training records) associated with them. The expectation would be, however, that the controlled documents and processes concerned with training and support would apply to *all* centre staff. There is no requirement for IT / data management specific policies or procedures.

GE01.01 Policies for training

Controlled documents are in place describing initial and continuing training requirements, policies and procedures

Having properly trained and competent staff managing trials and related systems is a GCP requirement. While it is not possible or appropriate for auditors to assess the competence of staff in the course of a short audit, it is possible for them to check that a centre has the proper mechanisms in place to promote and monitor staff competence.

Appropriate controlled documents should therefore exist that cover this area, detailing how initial induction as well as ongoing training should be identified, organised, signed off and recorded.

The expectation would be that induction and training was tailored to the individual's role as well as their previous experience, and which SOPs and other controlled documents any particular individual should be familiar with would be identified, so that they and their manager could ensure that they had familiarised themselves with them.

The evidence that the standard had been met would be the controlled documents themselves.

GE01.02 Documentation of training

Records of initial and continuing education are kept for all IT / DM staff.

All training should be documented. This would include, as a minimum, the dates and titles of training, but other details such as duration and training provider would also be useful. Individual folders can often include attendance certificates and programme details as well, and are usually combined with job description(s), CVs, records of publications etc. to create a comprehensive training and development portfolio.

The evidence that this standard had been met would be the training records themselves.

GE01.03 Identifying training requirements

A mechanism exists to identify training / development requirements, and document these and subsequent actions.

Training requirements will change, as a function of both general or organisational change (e.g. revised regulations or new systems) and individual development. Training must therefore be kept under review, and a mechanism to identify further needs and requests should exist and be documented on an individual basis, for instance within training folders, or as part of an annual appraisal mechanism.

Identifying desired or required training is only part of the story- for a variety of reasons training may not always be possible when first identified. There should therefore be some evidence that training requests are followed up, implemented, delayed or dropped, or dealt with by some other mechanism (e.g. temporary change in job responsibilities).

The evidence the standard had been met would come from interviewing staff and by inspection of the relevant records.

GE01.04 Problem resolution

Staff know who to go to within the organisation to seek advice and resolution of problems.

For time to time relatively serious problems or uncertainties may occur that cannot be resolved by normal informal discussion, for instance if there is disagreement about the ethics or legality of a proposed action (at the centre or one of the sites). In such cases staff should always know how and who they should approach for advice and guidance. This may be a different person (or group or committee) dependent on the nature or context of the problem, but clear 'escalation pathway' should exists.

The evidence that this standard is met would largely come from interviewing staff, discussing the organisation of the centre and its governance, and clarifying the escalation pathways available and how staff are made aware of them. Controlled documents covering this would make the standard much easier to confirm.

GE02 Site Management, Training & Support

These standards apply to the preparation and support of site staff by the staff of the data centre, with regard to data management and IT systems, and data entry and query management in particular. They are not directly concerned with overall site management issues such as site regulatory or ethical approval (though this is an indirect issue in GE02.04).

GE02.01 Policies for site opening and support

Controlled documents for opening and supporting a site for data collection are in place

Preparing and supporting site staff is a key function of any data centre and must be covered by relevant controlled documents. These would need to deal with (for instance) the training and preparation of site staff, the triggers that allowed access to production systems, the provision of documentation and ongoing support for sites.

The evidence would be the controlled documents themselves.

GE02.02 User training for data entry

User training with data entry instructions or guidelines, for pCRFs and / or eCRFs, is provided for site staff

Site research staff will need adequate preparation to correctly use pCRFs and / or eCRFs, delivered by preparatory training sessions, and / or self-study training material, written guidance, onscreen prompts and help documentation. The amount of preparation will vary with the experience of the site staff and the complexity and / or novelty of the study

The evidence that this standard is met will come from the records of training sessions and the distribution of training materials, and discussion with staff to clarify how the training is applied in practice.

GE02.03 Test or production environment

There is a clear and consistent on-screen indication to the user if they are working on a test or training eCRF.

For eRDC systems users should have the opportunity to familiarise themselves with a particular trial's CDMA within a test or training environment. It is vital, however, that the test and production environments are clearly distinguished, so that any staff member will not mistake one system for the other, and carry on putting test data into the real system:

In particular test or training eCRFs should be consistently and clearly marked, annotated or coloured to make this distinction clear. In some systems using a

graphic able to reference a different image in the test and production systems might work, and make the deployment of the two systems easier.

Though included in the standards for site staff, the same consideration also applies to internal centre staff who input data for pCRF based trials, and who need initial familiarisation with the trial's CDMA.

Evidence that the standard had been met would come from demonstration of the differentiation between production and test / training eCRFs.

GE02.04 Site access to production system

A site is given access to a production CDMA only once the sponsor, or the sponsor's representative, has confirmed that all relevant preparation, permissions and agreements have been completed

For eRDC trials the production CDMA should not be available to a site until that site has been fully prepared and approved. That normally means that all contractual agreements should have been signed, normally by both the site and the sponsor (or the data centre acting on the sponsor's behalf) and the relevant organisational and ethical approvals are in place. Only once this is the case can individuals be given access to the production system , when properly prepared (see GE02.05).

It is the sponsor's responsibility to make the decision about a site's preparedness. The data centre may be part of the same organisation, or be acting for sponsor in this respect, but in general the sponsor needs to inform the centre when a site is 'ready to go', and policies and procedures should reflect this.

For paper based trials the 'production CDMA' at the site is effectively the set of pCRFs, which may be delivered during the preparatory phase. pCRFs should not be accepted from the site, however, until it has been officially opened.

The evidence that the standard has been met would come from the relevant controlled documents, and demonstration by centre staff of how and when actual sites have been opened.

GE02.05 Individual access to production system

Individuals have access to production data only when they have been trained with the CDMS and the specific CDMA.

Individual staff should not have access to the production system until they have demonstrated competence in using the system in general, and the trial's specific CDMA in particular. The preparation required will depend on prior familiarity with the system. The recording of when competence is achieved and access enabled should be at the level of the individual, not the site.

The evidence that the standard has been met would come from the relevant controlled documents, and demonstration by centre staff of how and when individuals have been given access.

GE02.06 Site documentation

Processes exist to update and redistribute site documentation when this is required as part of change management

A site will need to store documentation relevant to the trial - particularly the protocol and guidance material related to completing the pCRFs / eCRFs. Should the protocol and / or CDMA change those documents will need revision and redistribution to sites, and mechanisms need to be in place to support this.

Evidence would come from demonstration of the mechanisms in action, usually within the CDMA change management process (see DM03.05).

GE02.07 Responsibility list

Processes exist to assure that up to date information of who can do what at each site, including entering data and / or signing off CRFs, is available to data centre staff

Centres need to know not only which staff at each site should have access to the production system, but also what the responsibilities of those staff are within the trial, allowing them to check that only properly authorised staff carry out tasks - for instance completing CRFs, carrying out the treatment allocation procedure, or completing a SAE form. If staff leave or are away for a reason (particularly the site's principle investigator) the centre needs to know to whom his or her duties have been delegated.

In short the centre needs to keep what is often known as a 'delegate log' covering the staff for each site in the trial. How that log is maintained will differ from centre to centre - some may use monitoring or other staff visiting the centres to keep the centre informed of changes, others may ask site staff to send the details in directly to trial managers. Either way the requirement is that a list is available to data entry and trial management staff.

Evidence that the standard had been met will be

a) the presence of lists of staff and responsibilities for sites

b) controlled documents that describe how such lists are obtained and kept up to date as much as possible

GE02.08 User Support - prompt response

The centre is able to provide Help Desk support and / or web based support (details as agreed with sponsors) to provide a rapid initial response to site requests

User support needs to be maintained during the course of the trial, and that includes the prompt response to queries or requests for help from site staff. Such support might involve a telephone hot line or it may be a web based system.

The precise nature of this support will depend on the centre's and trials sponsor's judgement about what is required, and the resources that have been made available to provide it. The requirement is that the centre is able to provide some form of prompt user support when resourced to do so.

As evidence that this is the case the centre staff would normally be expected to provide examples of current support agreements and mechanisms.

GE02.09 User Support - in English

Help desk / web support can be provided in English as well as the data centre's native language

With multinational trials user queries and requests may arrive in a variety of languages. No centre can be expected to support all the potential languages staff might use in a cross European trial, but there is a requirement that they can provide such support in English at least.

Evidence would come from direct observation.

GE03 Treatment Allocation

These standards deal with all forms of treatment allocation, i.e. both traditional randomisation, normally using permuted-block allocation, and minimisation and other deterministic methods. They are also concerned with the whole treatment allocation process, not just the parts supported by IT systems or IT and data management staff. Input from statisticians, in particular, is included in the scope of the standards.

If a data centre uses an external agency to provide some or all of its treatment allocation services, then it needs to have the evidence available that the external agency, where necessary, also complies with the relevant standards.

GE03.01 Procedures for treatment allocation

Controlled documents are in place dealing with the set up and management of treatment allocation

Whatever the treatment allocation method(s) used, there should be clear policies and procedures in place governing how treatment allocation should be set up and then managed.

The relevant controlled documents would provide the evidence this standard had been met.

GE03.02 Policies for ensuring blinding

Controlled documents exist covering the preservation of blinding (where used)

Though not all trials can be easily blinded (e.g. surgery and radiotherapy trials, and oncology trials involving chemotherapy) most trials that involve only oral medication will be double blinded.

In such cases it is necessary to have clear policies about how blinding is established and should be maintained (these will often cover distribution of the labelled drug as well).

The relevant controlled documents, together with explanations of how they are applied in practice, would form the evidence that this standard had been met.

GE03.03 Policies for Unblinding

Controlled documents are in place to support rapid and safe unblinding of blinded treatments when required

Clear procedures are required, in the context of blinded trials, that describe how - when the need arises -blinding can be removed. Unblinding policies should normally cover the unblinding sometimes necessary for individuals, e.g. in the

context of a SUSAR, and that sometimes requested for whole treatment groups, e.g. in the context of a data monitoring committee meeting.

The relevant controlled documents, together with explanations of how they are applied in practice, would form the evidence that this standard had been met.

GE03.04 Algorithms and supporting systems

The underlying algorithms and operations of all systems for allocating subjects to treatments must be clearly documented and validated

The systems used for treatment allocation may vary considerably in sophistication, but they should be documented so that the underlying algorithms are clear (or if published are referenced). They should also be validated, whether or not they are IT based, to check that they operate as intended.

If a centre uses an external IT based service for its treatment allocation then it still needs to satisfy itself that the system has been validated, normally by the service providers, and that the evidence of that validation is available.

It is good practice to monitor the allocation decisions made within any trial, and the size and characteristics of the treatment groups, to check that the algorithm is continuing to function correctly.

Evidence that the standard has been met would come from the documentation of the systems, the algorithms used and the relevant validation documents.

GE03.05 Specification documentation

Details of the treatment allocation specification for any specific trial should be documented and recorded

The broad methodology to be used for treatment allocation will normally be included in the protocol, but each trial will also have its own detailed specification, usually determined by the trial statistician (though the sponsor will have the final decision).

This detailed specification, for instance including data on block size, stratification factors, or the random element within a minimisation scheme, needs to be documented.

The evidence is provided by examples of detailed trial specific treatment allocation specifications.

GE03.06 Problem Management in Treatment Allocation

Any problems or errors that arise in the treatment allocation process are logged and the subsequent actions recorded

Occasionally errors can arise in the treatment allocation process - subjects being allocated twice, or, if stratification or minimisation criteria were not collected accurately, being allocated to the wrong treatment group. Such cases , and the actions taken as a consequence of them, should be recorded.

The documentation of the allocation errors and the subsequent actions, together with relevant controlled documents, provide the evidence that the standard has been met.

GE03.07 Treatment Allocation Training

All staff who handle allocation requests are adequately trained for each specific trial randomisation process

Treatment allocation is often complex and cannot always be completely automated. Where staff are involved, even if it is just noting down stratification criteria, they must be adequately trained so that errors do not occur.

Evidence that the standard had been met would come from records of training and explanation about how treatment allocation is distributed amongst staff within the centre.

GE03.08 Record of Allocation

Records of all allocation material generated and all allocation decisions made must be maintained

The treatment allocations made during a trial are a vital part of that trial's history and must be retained, for as long as the trial data is retained.

This means keeping the original randomisation lists, and the minimisation decisions in their correct order (i.e. context),and not just the resulting treatment allocations. Controlled documents would normally specify the process by which this data was stored, as well as the access control required.

These controlled documents, together with examples of the lists themselves, would provide the evidence that the standard had been met.

GE03.09 Failover to Manual

System(s) must be in place, supported by training, to deal with a loss of IT based treatment allocation (if used)

When treatment allocation uses IT there is always the problem of what to do when for some reason that IT system is unavailable. Treatment allocation should still be able to continue if subjects are presented for inclusion. A centre must

therefore have systems in place to cope with this situation, for all trials being allocated at any one time, with the staff involved suitably trained to use whatever methods have been identified as suitable.

Manually allocating treatments from permuted block lists is usually fairly straightforward, but manually applying minimisation algorithms can be complex, and may demand specialist expertise. In either case there will be the need to ensure that once restored the IT based systems are brought up to date with any allocations that may have occurred when they were down.

The relevant controlled documents, training records and discussions with staff would form the evidence that the standard had been met.

GE04 Transferring Data

Transferring data refers to sending the data out of the centre, not just removing it from the CDMS (which is data extraction or export).

Transferred data will leave the centre's IT network completely and be sent to another institution. It may occur in the context of a collaboration or meta-analysis, or sending data to a statistician or investigator based elsewhere for analysis or review. For an industry sponsored trial it may include sending data to that sponsor.

GE04.01 Data Transfer Procedures

Controlled documents dealing with the transfer of data from the data centre should be in place

This standard requires that there are controlled documents that describe the principles to be followed when transferring data, including the documentation required.

The evidence that the standard had been met would come from the controlled documents themselves.

GE04.02 Encryption of Individual Data

Any file(s) transferred out of the data centre that include data relating to individuals should be encrypted

If transferred data includes data relating to individuals it must be encrypted, to the level considered as good practice by the national regulatory authority (currently 128 or 256 bit AES encryption). This reflects the difficulty in distinguishing patient identifying data from other data relating to individuals (see IT02.03).

Encryption may occur before transfer, which would be required if the medium is a portable device like a USB stick or CD. In some cases encryption may take place *during* electronic transfer, when using a secured system, as is sometimes the case when sending data to industrial sponsors.

Because transferred data may be commercially sensitive even when it does not include individual level data, it may be safer and easier to routinely encrypt *all* such data. Sending encrypted data electronically as an attachment is now very difficult because recipient systems will normally remove it as an unknown and therefore potentially malicious file. Encrypting all data allows the development of a single procedure for the physical transfer of all data.

Evidence that the standard had been met would include the relevant controlled documents and explanation of how encryption of transferred data was carried out in practice.

GE04.03 Purpose Recorded

The purpose of the planned data transfers should be known and documented

Final decisions about who to send data to and when will rest with the sponsor or a trial management group acting on the sponsor's behalf. A centre should still, however, have procedures in place that ask how the data will be used, partly to support the transfer process for the sponsor and partly to help protect its own reputation and that of its parent organisation.

Evidence that the standard had been met would come from the documentation associated with data transfers.

GE04.04 Assuring Security

The centre sending the data must have a written agreement / declaration from the recipient that the receiving organisation will maintain appropriate security of data (whilst it remains in their direct care)

The data centre should assure itself that the data will be kept securely at the recipient organisation, normally asking for a summary of how the data will be stored and who will have access to it, to help protect its own reputation and that of its parent organisation.

(This is one reason that transferred data should never be sent to personal web based email accounts, like Gmail or Hotmail, as their security cannot be guaranteed).

Evidence that the standard had been met would come from the documentation associated with data transfers.

GE04.05 Format of Data Transfers

Procedures should be in place for agreeing, specifying and documenting the format of the transferred data

The format of the transfer will depend on whatever is agreed between the centre and the recipient. The centre only needs a mechanism to agree the best format(s) to use.

A data transfer proforma that can be sent to the recipient for completion allows this and the other data required to be captured in a structured way , making discussion around and documentation of the whole process easier.

Evidence that the standard has been met would come from the relevant controlled documents and from the documentation associated with data transfers.

GE04.06 Records of Transfers

Details of any specific data transfer should be logged, and include a summary description of the data, sender, recipient and transfer method, and the date sent

Once the transfer takes place it needs to be recorded, and include the data listed in the standard.

Evidence that the standard had been met would come from the documentation associated with data transfers.

GE04.07 Retention of Copies

Copies of the data sent should be retained within a read only regime and be available as a reference data set for audit / reconstruction purposes.

After the transfer takes place the centre should keep copies of all data sent, for audit purposes and in case it needs to be sent again, in a read only section of its storage capacity (i.e. read only apart for the IT staff who need to put the data in that location).

Evidence that the standard had been met would come from demonstration of transferred data in an appropriate read only environment.

GE04.08 Retention of post-processed data

If data is processed before being transferred, copies of the data as extracted before post processing should be retained as well as copies of the data actually sent

It is not uncommon for data to undergo some form of pre-processing before it is transferred outside of the data centre, for instance to the format agreed by the sender and recipient if that is not the same as that generated natively by the CDMS (e.g. conversion to CDISC ODM).

In these circumstances it is important that the data as originally extracted (as well as that which is finally transferred) is retained in a read only environment, so that a complete history of the transfer process is available and, if necessary, the post processing can be checked and / or repeated.

Evidence that the standard had been met would come from demonstration of both extracted and transferred data in an appropriate read only environment.

GE05 Receiving and Uploading Bulk Data

Centres often need to upload and import bulk data from a variety of external sources: laboratories (e.g. biomarker data), instrumentation (e.g. the settings from a radiotherapy machine), collaborators (e.g. data from another set of sites), or even the sponsor (e.g. SAE reports).

There are usually two stages to the process - firstly data receipt into the centre, i.e. of the data files as sent from the source, and secondly data upload or import into the data centre's own systems. In many cases the data will need to be processed in some way before the second upload stage can begin.

The data uploads may be directly to the trial's CDMA, or they may be to a data repository system that itself receives data from the CDMA, allowing aggregation of all the data, whatever its source, before a combined extraction for analysis.

GE05.01 Import Procedures

Controlled documents dealing with receiving and uploading bulk data should be in place

The receipt / upload process should be governed by pre-specified generic procedures and processes, as required by this standard.

In practice each upload process will also probably need its own more detailed procedural guidance if consistency is to be maintained, especially if - as is often the case - the data needs transforming in some way before it is imported.

The relevant controlled documents would provide the evidence that this standard had been met.

GE05.02 File Retention

The original files received should be retained within a read only regime, and be available as a reference data set for audit / reconstruction purposes.

To ensure a full audit record, and in case the import needs to be repeated for any reason, it is important to keep copies of the data as originally received.

Evidence that the standard had been met would come from demonstration of the original data in an appropriate read only environment.

GE05.03 Retention of post-processed data

If imported data has to be pre-processed before upload to the CDMS, copies of the data actually uploaded should be kept within a read only regime

When data must be processed before it can be imported then, unless that processing is trivial, predictable and can be quickly repeated, the data as it stands

after processing should *also* be kept, i.e. the data that is actually imported into the system.

Processing in this context may not just take the form of a consistent transformation, e.g. of format or data type. Especially when data arrives in a relatively unstructured form such as a spreadsheet, it may also be necessary for the data to be scanned for any values that appear to be errors or out of normal range, and make the necessary corrections in a more ad hoc way.

Evidence that the standard had been met would come from demonstration of both received and imported data, i.e. each side of the pre-processing, in an appropriate read only environment.

GE05.04 Logging of receipts and uploads

Each receipt and upload process should be documented and logged

The receipt and upload process itself should be logged (though recording the actual import may be an automatic function in some CDMSs). Logging should normally include the source organisation, a summary of the contents, the location of the copies of data, and the date, as well as any problems that arose during the import.

If importing into a CDMS, it is very useful if the system can apply the normal validation logic that is used during manual data entry to the incoming data, generating warnings etc., and allowing a review of any problematic data items (though this is not a formal requirement).

Evidence that this standard had been met would come from the receipt / upload logs themselves.

GE05.05 Format of received data

Procedures should be in place for agreeing, specifying and documenting the format of the received data

As with data transfer out of the centre (see GE04.05) the format of the data received should be agreed between the centre and the source organisation. The centre therefore needs a mechanism to agree the best format(s) to use.

Evidence that the standard has been met comes from the relevant controlled documents and from the documentation associated with arranging data imports.

GE05.06 Requests for direct amendment

Procedures should exist to deal with requests for direct changes of data in the database

This standard deals with a relatively unusual situation, and one that some centres may never experience. It involves the need to directly change data in the back end database or file store, rather than going through the normal CDMA user interface.

Such situations can arise if data is imported to a CDMA in bulk (so there may not be an eCRF corresponding to it in the system) and then needs correcting. If the data import is a regular event and designed to over-write existing values there is little problem - the data can just be re-imported with the corrected values.

If the original input was intended as a one-off, however, then any amendments required will need to be done manually on an ad hoc basis. An example might be an imported treatment allocation list (i.e. subject trial ID against treatment received, A or B) that had to be amended because one or two subjects were found to have received the wrong treatment.

A centre should be prepared for such a situation, or prohibit it entirely and insist on another method of editing the data (e.g. by repeated bulk upload according to a specific procedure). If the centre does allow direct data amendment, then because each change request will be different there is little a centre can do other than have a very generic procedure, for instance that identifies how the change request would be considered and by whom, who would carry out the action decided upon and how the whole process should be fully documented.

The evidence for compliance would be the procedure itself.

GE05.07 Recording direct amendments

Any direct amendments made must be logged and the details noted, including the justification for the change

If direct amendment of data does take place (see GE05.06) then it needs to be recorded, with all details noted and communications (emails etc.) retained, and this should be made clear in the associated controlled documents.

The evidence that the standard had been met comes from the documentation of such changes or, if no such incidents have occurred in recent years, at least the controlled documents that dictate the recording required.

GE06 Long Term Data Storage

Trials eventually reach a point when data is no longer being input, all outstanding queries have been resolved and all the anticipated papers have been written. Direct access to the trial data, in paper or electronic form, is either no longer required or limited to occasional read only access. At this point the trial enters long term data storage.

The trial is not necessarily formally 'archived' or curated at this point. It could be, though very few data centres appear to have mechanisms in place to provide a full digital curation service for electronic data, even if many have separate long term storage facilities (which may or may not be called an 'archive') for paper based data.

The characteristic of long term storage is restricted access and thus protection from change. The trial's electronic documentation and its data become hidden or read only (though some at least of the IT staff need to retain access in order to resurrect the data to active use if necessary). Its paper data records are moved away from the normal storage locations and into a special store reserved for old, no longer active records, which may not be at the same physical location as the rest of the centre.

In the future keeping electronic data over the long term may also mean changing the format of that data, to make it less dependent on proprietary systems that may disappear in the future. Possible target formats are CSV files or XML, e.g. using the CDISC ODM format. The latter has the great advantage of being able to include metadata definitions as well as the data. Anonymising the data so that storage can be encryption free (which avoids the difficult issue of long term key management) is another useful technique for long term curation.

At the moment the standards do *not* include such active data transformations, though they may in the future, especially as long term curation becomes a more prominent issue and these techniques become more common.

GE06.01 Policies for long term storage

Controlled documents are in place concerning long term storage of both trial documents and electronic data

Moving a trial's data and documents to long term storage should be the subject of controlled documents that describe the overall approach and procedures, roles and responsibilities.

Evidence that the standard had been met would be the controlled documents themselves.

GE06.02 Access to long term storage

Access to physical and electronic long term storage is controlled and removal or re-activation of any documents or data is recorded

Access to the data in long term storage is controlled, usually with designated staff acting as the 'gatekeepers' to the stored material. This allows access or re-activation to be recorded.

This may take the form of logging the extraction of documents out of long term physical store, e.g. by whom and when (partly because there will often be a need to check that the material is returned). When access to electronic data needs to be re-activated this too should be logged, and restrictions re-applied if and when necessary.

The evidence that the standard had been met would come from the records of access and / or re-activation.

GE06.03 Protection of long term storage

Measures are in place to guarantee secure long term storage (e.g. locked rooms and fire-proof cupboards)

Long term storage must be secure to be useful. For paper based records this means environmental protection for documents (against fire, damp etc.) and the ability to lock individual cabinets or shelving so that access to one group of documents does not mean access to all.

For electronic data it means mechanisms to ensure copies in multiple places. (Note that backup systems are usually configured to provide relatively short term redundancy and security and are not normally intended to cope with long term storage. Other mechanisms may therefore need to be used to provide redundancy in the long term). In many cases data in electronic long term storage stays within the normal storage capacity of the centre, but is just not visible to normal users.

Evidence that the standard had been met would be provided by inspection of long term storage facilities, discussion of the access regime for long term electronic storage, and by the procedures described in the relevant controlled documents.

GE06.04 Length and Content of Storage

Procedures should be in place to agree with the sponsor the length and content of long term storage.

The material (paper and electronic) that is placed in long term storage is there partly so that the data can be consulted when necessary, partly so that the trial itself can be re-examined and its design, implementation and results can be fully understood.

This does not mean that it is necessary to be able to restore the trial's data and systems to exactly their original state - systems will move on, change versions, even whole applications - but it does mean being able to inspect the sequence of the major events within the trial, as well as the data and the generic and trial specific documents that formed the framework within which it operated.

The key to being able to do this is selecting the right material at the beginning of the long term storage process. Ultimately this will be the sponsor's decision but the centre, if it is responsible for carrying out the long term storage, should have procedures in place to agree that content with the sponsor.

Evidence that the standard had been met would be the relevant procedures, as described in controlled documents, and examples of their use in practice.

GE06.05 Data Destruction

Procedures should be in place to support the final destruction of physical and electronic data, as required by regulations and/or sponsor

Eventually, data and documents will usually be destroyed. When will again be determined by the sponsor, as well as by national regulations. The centre's procedures should therefore include mechanisms to identify with the sponsor the retention periods of the data in storage. Those dates should then be available to current and future staff, perhaps integrated into the storage content records.

Evidence that the standard had been met would be the relevant procedures, as described in controlled documents, and examples of their use in practice.

Requirements for Certification of ECRIN Data Centres
with
Explanation and Elaboration of Standards
Version 2.2, July 2012
